# Supplementary material for: TDP-43 proteinopathy in ALS is triggered by loss of ASRGL1 and associated with HML-2 expression
Source: Nat Commun. 2024 May 16;15:4163. doi: 10.1038/s41467-024-48488-7 (PMC11099023; doi:10.1038/s41467-024-48488-7)
Supplement: Supplementary file 1 — Supplementary Information [file 41467_2024_48488_MOESM1_ESM.pdf]

SUPPLEMENTARY INFORMATION

TDP-43 proteinopathy in ALS is triggered by loss of ASRGL1 and associated with HML-2 expression.

Marta Garcia-Montojo[1], Saeed Fathi[1], Cyrus Rastegar[1], Elena Rita Simula[1,2], Tara Doucet-O'Hare[1], Y-H. Hank Cheng[1], Rachel P.M Abrams[1], Nicholas Pasternack[1], Nasir Malik[3], Muzna Bachani[3], Brianna Disanza[1], Dragan Maric[4], Myoung-Hwa Lee[1], Herui Wang[5], Ulisses Santamaria[1], Wenxue Li[1], Kevon Sampson[1], Juan Ramiro Lorenzo[6], Ignacio E. Sanchez[7], Alexandre Mezghrani[1], Yan Li[8], Leonardo Antonio Sechi[2], Sebastian Pineda[9], Myriam Heiman[9], Manolis Kellis[9], Joseph Steiner[3] and Avindra Nath[1]*.

Correspondence to: natha@ninds.nih.gov

This file includes:

- Supplementary Figures 1 to 14
- Supplementary Tables 1 to 5

Supplementary Figures

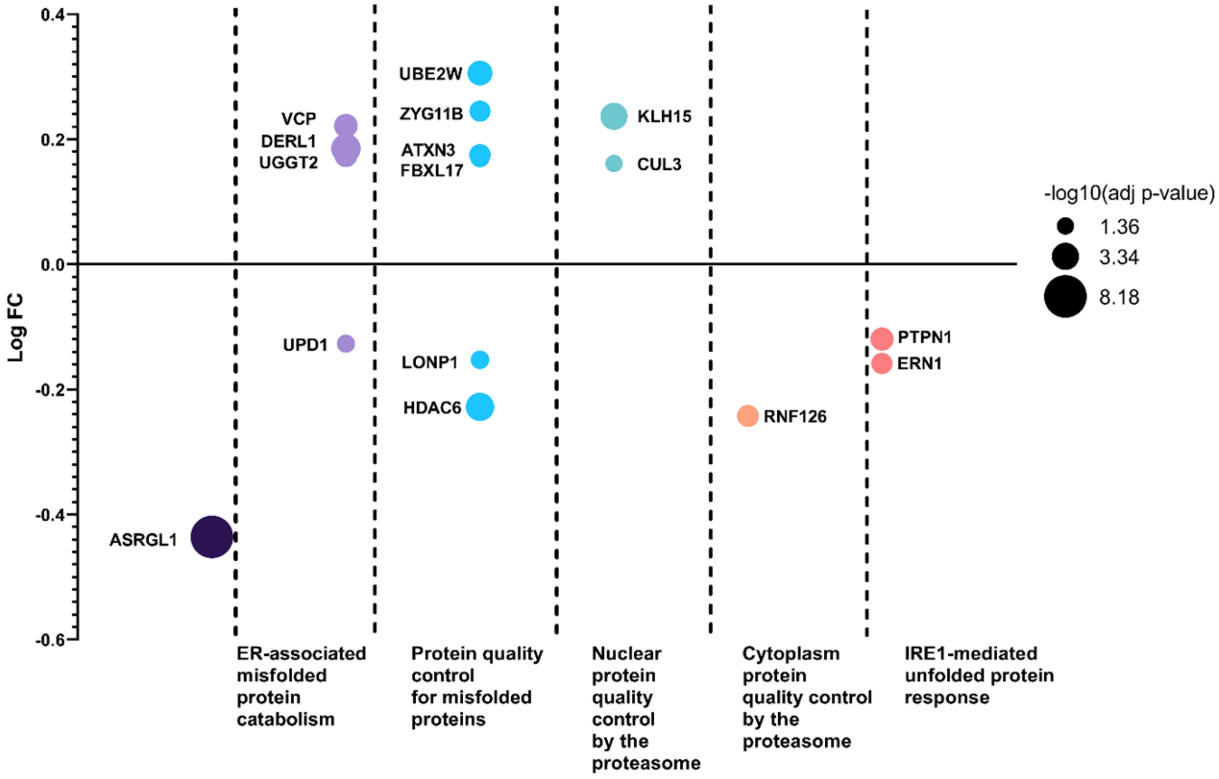

Supplementary Fig. 1: Differentially expressed genes in ALS and controls by RNAseq. Bubble-plot showing differentially expressed genes in ALS (n=323) and control (n=68) frontal cortex samples involved in biological pathways associated with ASRGL1.

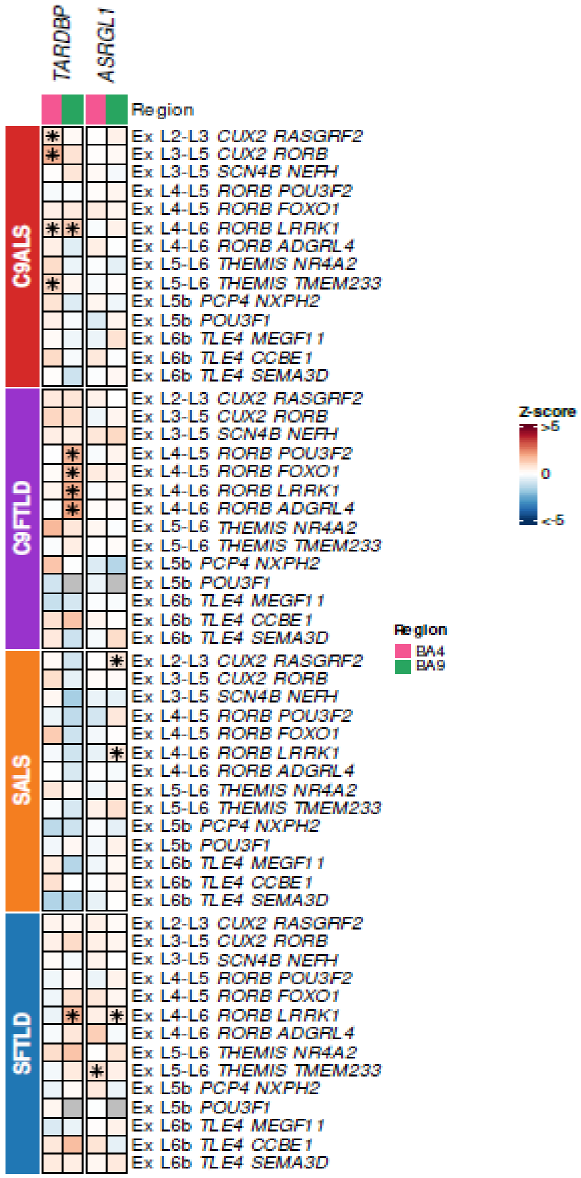

Supplementary Fig. 2: Single cell sequencing analysis of ASRGL1 and TARDBP RNAs in excitatory neurons. Single cell sequencing data of RNA expression in Motor cortex (BA4) of a cohort of 73 individuals (sALS; n=17, C9orf72-associated ALS; n=16, sporadic FTLD; n=13, C9orf72-associated FTLD; n=11 and non-neurological controls; n=16). BA9 was analyzed as a control.

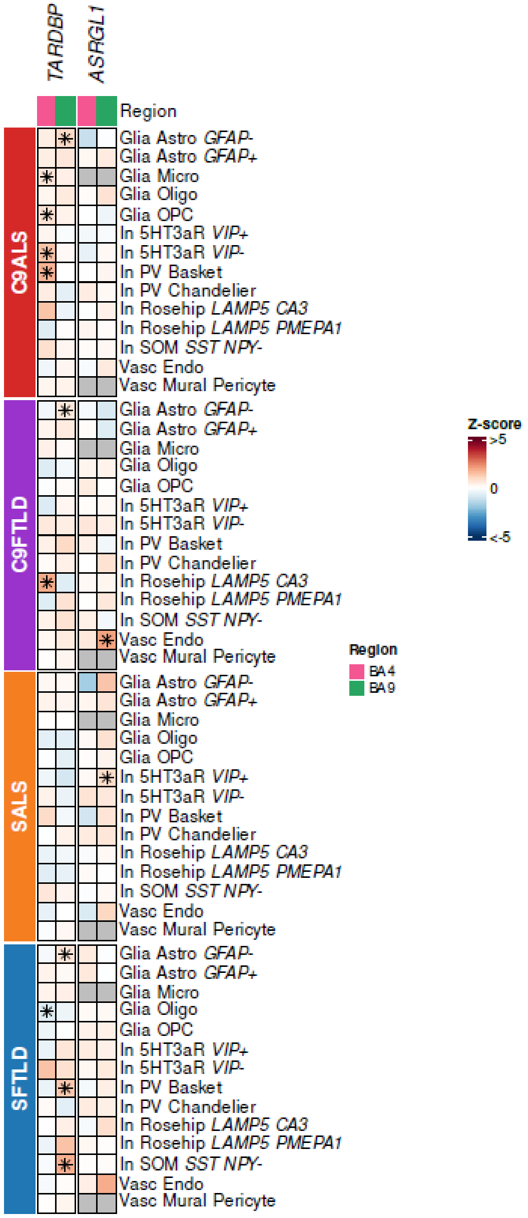

Supplementary Fig. 3: Single cell sequencing analysis of ASRGL1 and TARDBP RNAs in inhibitory neurons and glia. Single cell sequencing data of RNA expression in Motor cortex (BA4) of a cohort of 73 individuals (sALS; n=17, C9orf72-associated ALS; n=16, sporadic FTLD; n=13, C9orf72-associated FTLD; n=11 and non-neurological controls; n=16). BA9 was analyzed as a control.

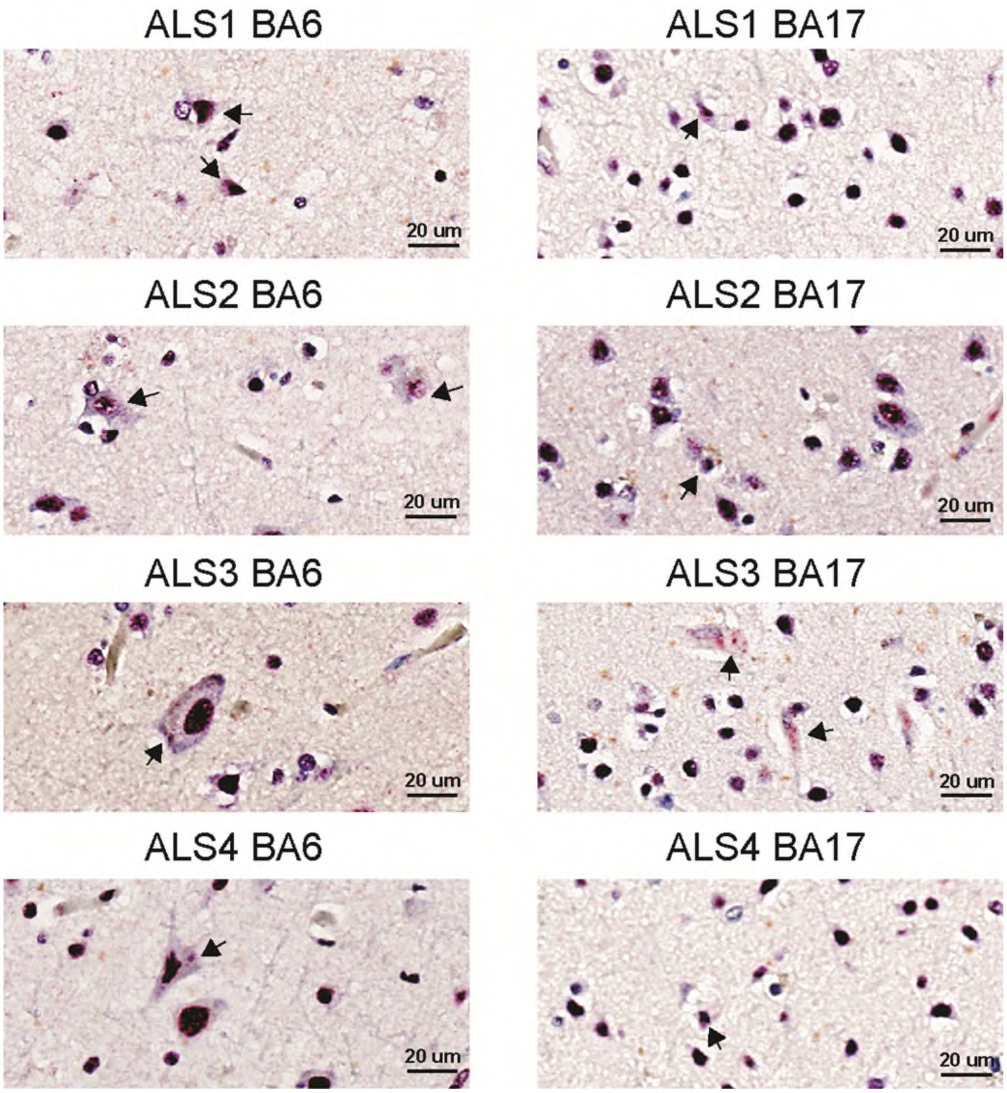

Supplementary Fig. 4: Immunostaining of TDP-43 on cortex samples of ALS individuals. Pre-motor cortex (BA6) and visual cortex (BA17) of the same individuals (n=4) were stained. Images show deposits of cytoplasmic TDP-43 (black arrows).

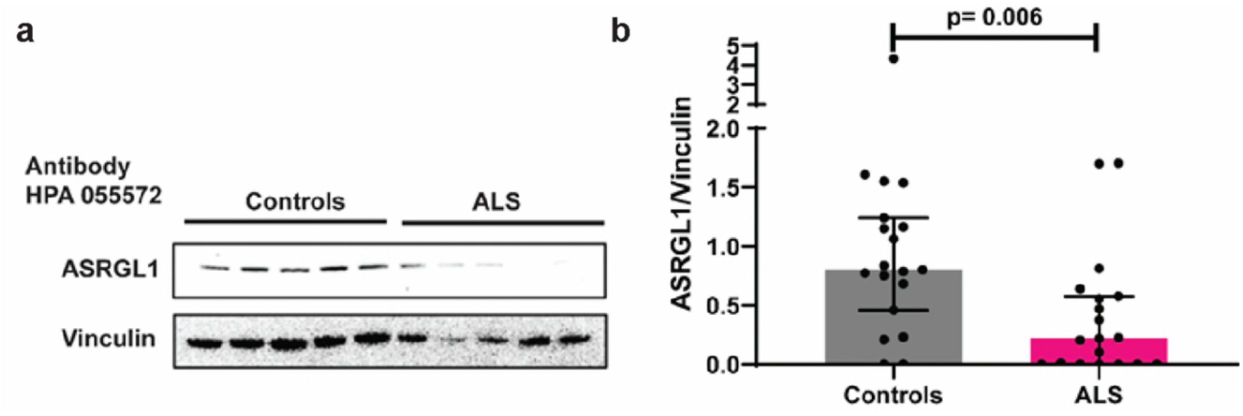

Supplementary Fig. 5: Lower expression of ASRGL1 in the cortex of patients with ALS than in controls, analyzed by western blotting. Pre-motor cortex (Brodmann's area 6) samples from 20 ALS patients and 20 normal controls were lysed and analyzed by western blotting to measure ASRGL1 protein with one additional antibody. a) Representative image of a western blot showing the expression of ASRGL1 and the loading control vinculin in controls and ALS patients stained with anti-ASRGL1 antibody (Atlas antibodies; HPA055572). b) Graphic representation of the levels of ASRGL1 as measured with anti-ASRGL1 antibody (Atlas antibodies; HPA055572) (**p<0.01, Mann-Whitney test).

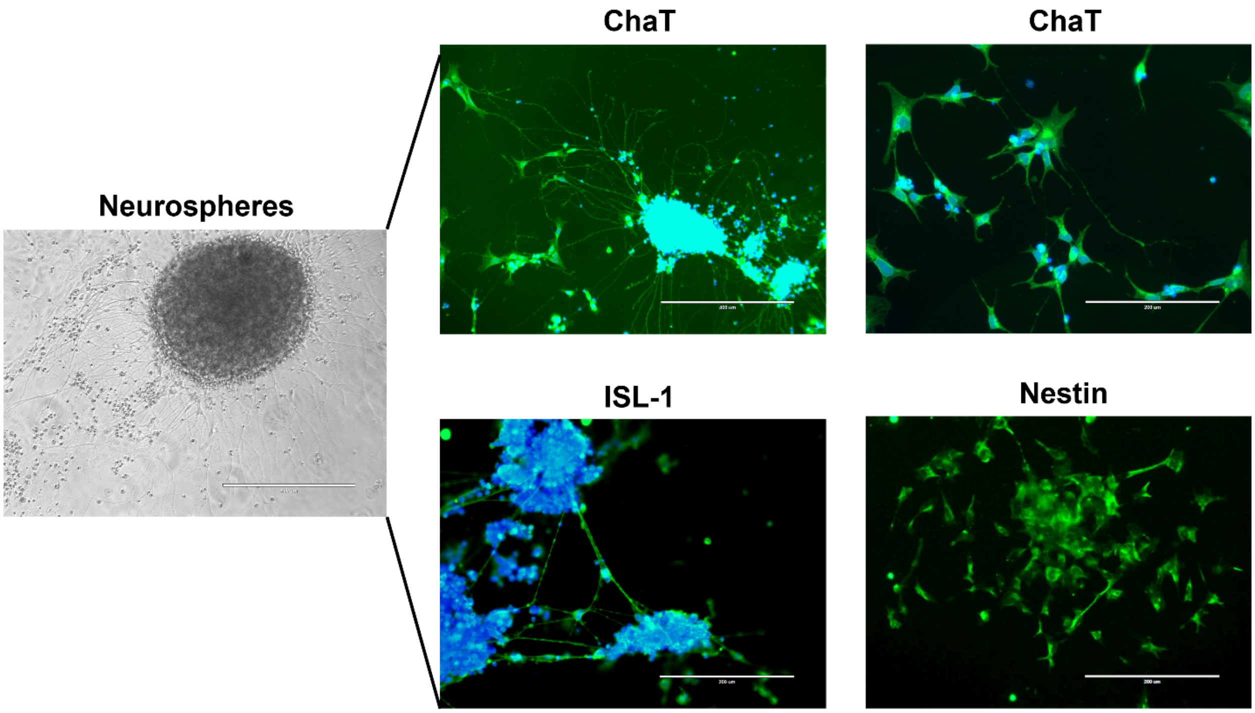

Supplementary figure 6: Characterization of IPSc-derived motor neurons. The cells were seeded as aggregated spheres to get better motor neuronal differentiation. 95% of the differentiated neurons (with long neurites, grew out from the neurospheres) were ChAT positive, indicating the lower motor identity. These cells were also ISL-1 positive, to a lesser degree. The other 5% of cells could not be identified, but based on their morphologies, they were likely glial cells, though at this stage they were still GFAP negative. Although it was difficult to clarify the cell types in the center of the aggregated neutrospheres precisely, they were mostly undifferentiated neural stem/progenitor cells with positive nestin staining.

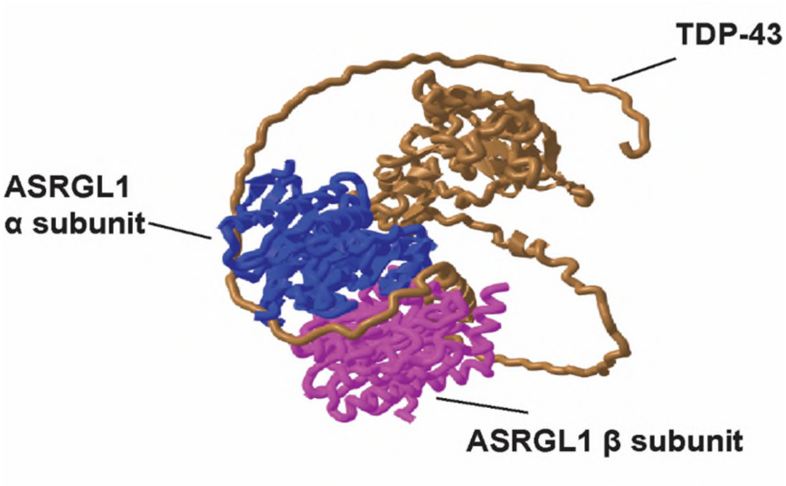

Supplementary Fig. 7: Docking of TDP-43 and ASRGL1 by ClusPro 2.0.

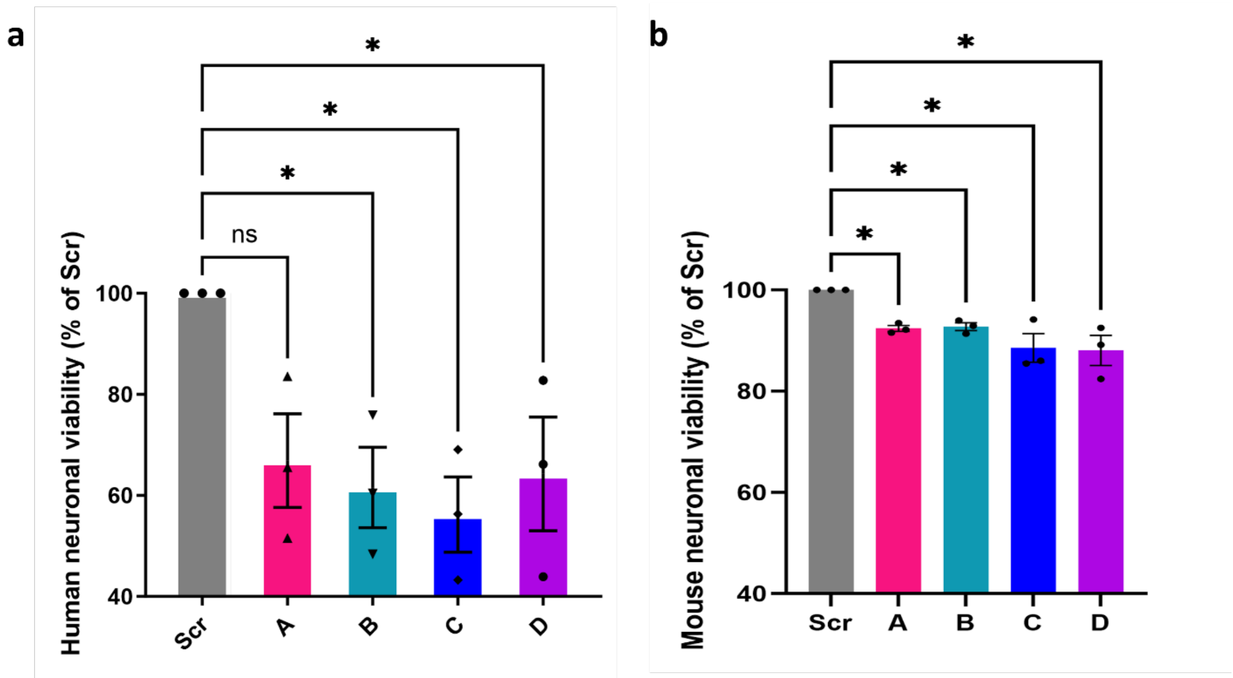

Supplementary Fig. 8: Neurotoxicity of individual shRNAs against ASRGL1 measured by cell count. Comparison of cell viability, as measured by cell count in an automated flourescence microscope in **a)** IPSc-derived tdtomato-expressing human neurons and **b)** Stably GFP- expressing mouse neurons derived from primary neural stem cells transfected with individual shRNAs against the human and the murine sequences of ASRGL1 (Brown-Forsythe ANOVA test with Bonferroni correction; Mean ± SEM; number of experimental replicates=3).

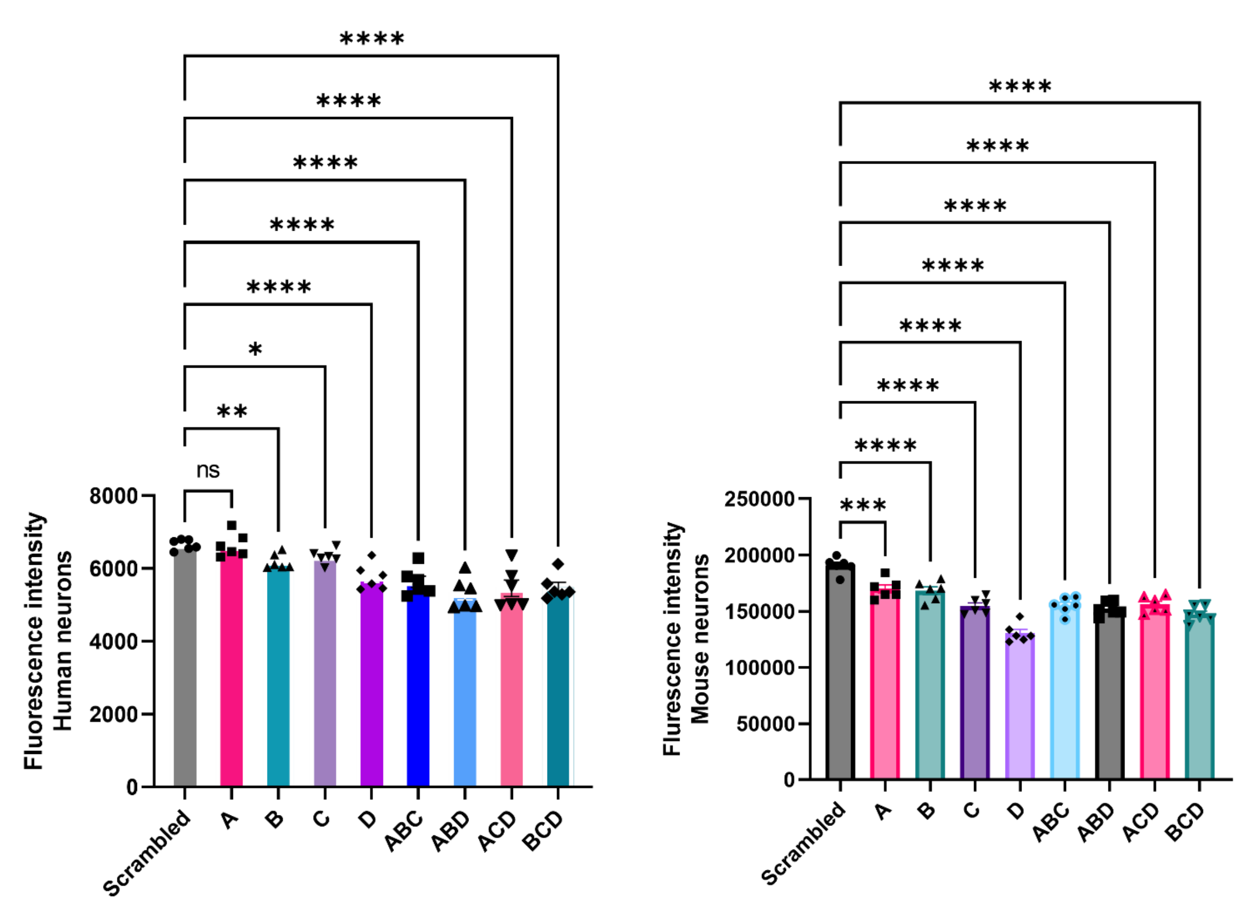

Supplementary Fig 9: c. IPSc-derived human neurons and Mouse neurons derived from primary neural stem cells transfected with individual shRNAs against the human and the murine sequences of ASRGL1. Comparison of cell viability as measured by Alamarblue in **a)** human neurons and **b)** mouse neurons transfected with individual shRNAs against the human and the murine sequences of ASRGL1, as measured by fluor spectrometry (number of experimental replicates=6; One-way ANOVA).

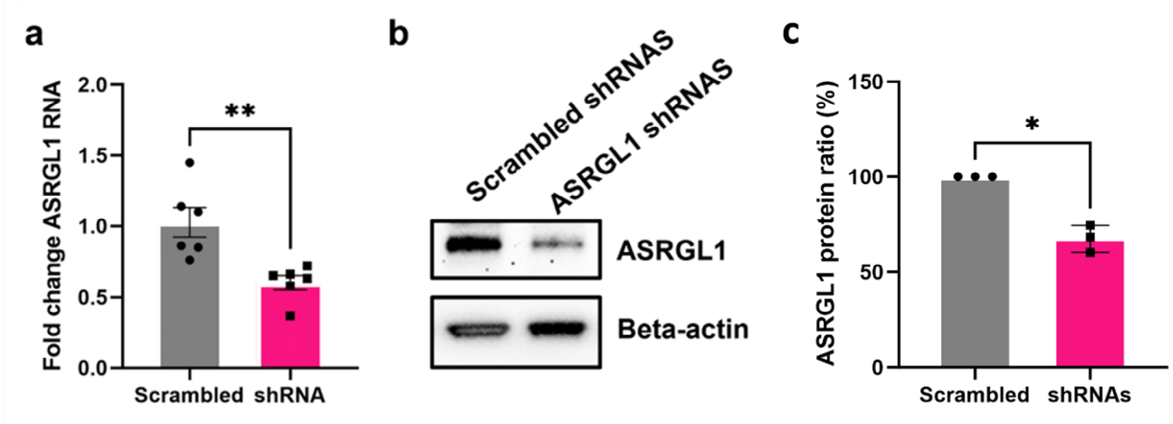

Supplementary figure 10: Validation of ASRGL1 silencing effect of the combination of shRNAs BCD. a) by PCR (Unpaired T-Student test; Mean ± SEM; number of experimental replicates=6). and b, c) western-blotting (Mann-Whitney test; Median (IQR); number of experimental replicates=3).

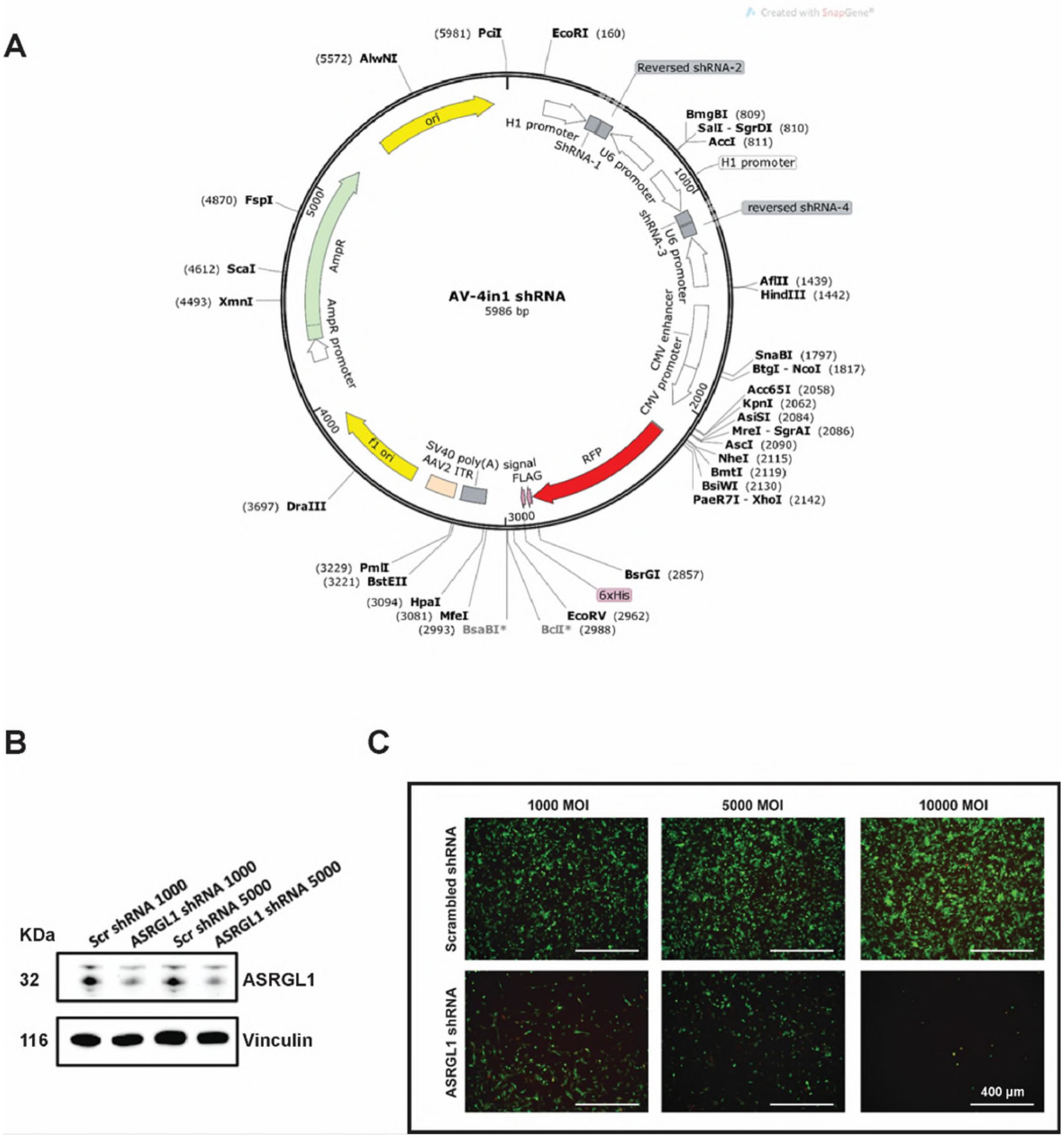

Supplementary Fig. 11: Transduction of mice neural cultures with AAV9 viral particles packing shRNAs causes cell death in dose responsive manner. (A) Map of the DNA construct encoding ASRGL1 shRNAs packed in AAV9 viral particles. (B) Representative image of a western blot showing the knocking down of ASRGL1 in neurons after transduction. (C) Fluorescence microscope images showing the change in cell viability in GFP-expressing mouse neurons transduced with AAV9 viral particles at increasing multiplicity of infection (MOI).

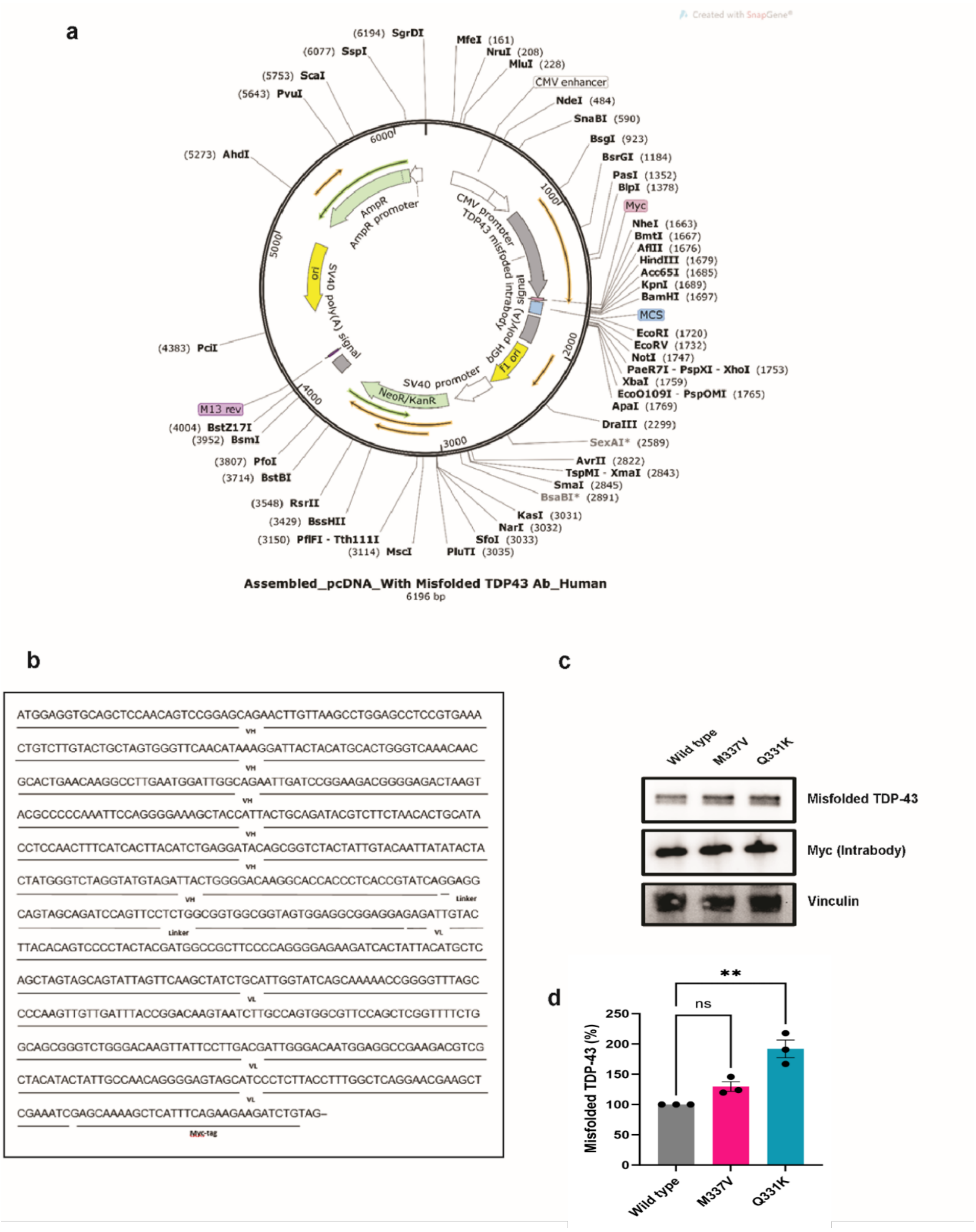

Supplementary Fig. 12: Cloning and validation of an intrabody against misfolded TDP-43. a) Map of a DNA construct encoding an intrabody against misfolded TDP-43. b) Sequence of TDP43 intrabody-Myc. VH: Variable Heavy Chain; VL: Variable Light chain. **c, d)** HEK 293 cells were co-transfected with 1) the plasmid encoding the intrabody against misfolded TDP-43 (Myc-

tagged) and 2) Wild-type TDP-43 or ALS-associated TDP-43 mutants M337V or Q331K that are prone to misfolding. **c)** Representative blot the experiment. **d)** Comparison of the levels of the misfolded TDP-43 pulled down with the intrabody in cells transfected with the aberrant forms and the wild-type TDP-43 (Brown-Forsythe ANOVA test with Bonferroni correction; Mean ± SEM; number of experimental replicates=3).

.

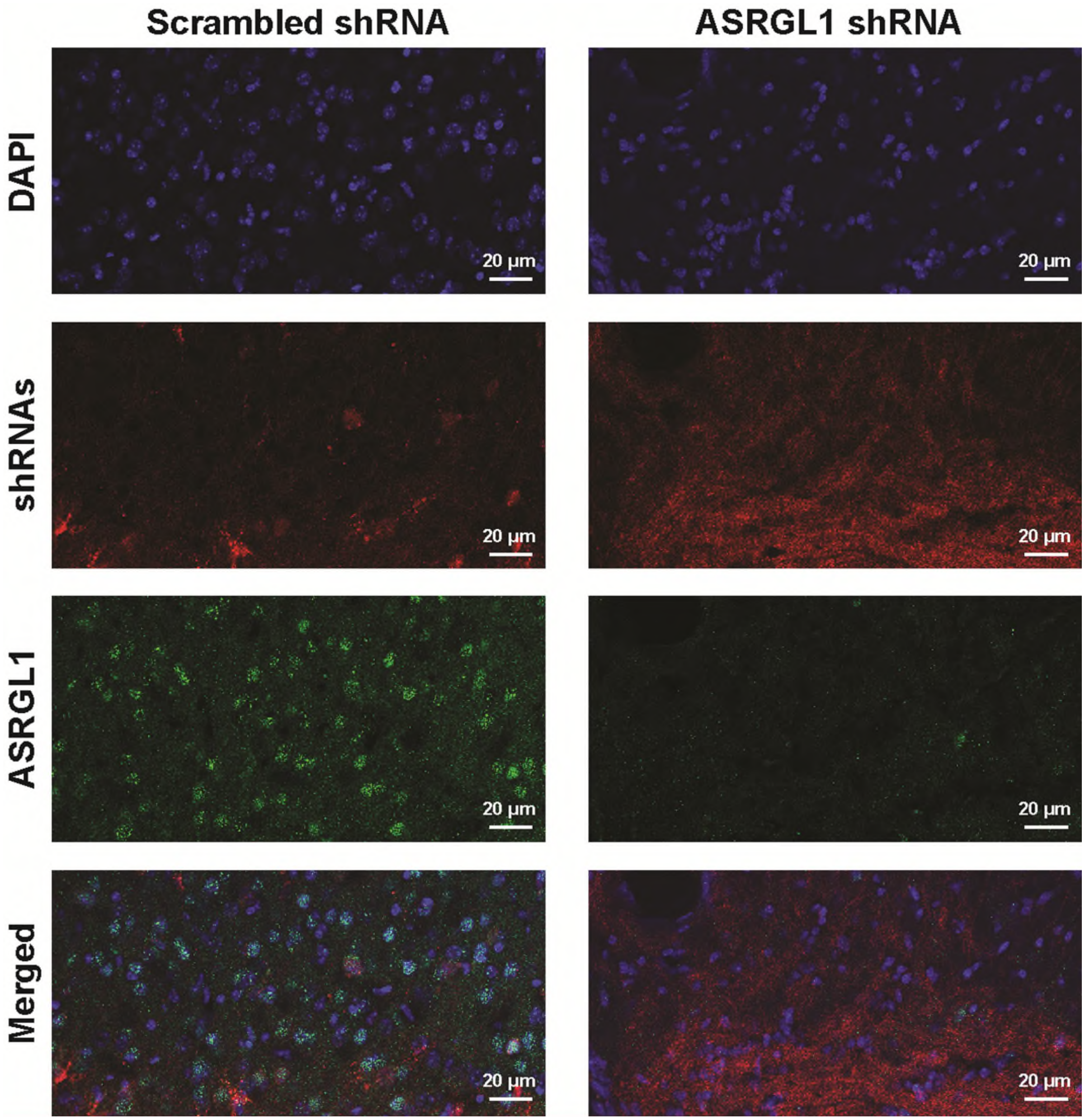

Supplementary Fig. 13: ASRGL1 silencing by injection of AAV particles carrying a construct with 4 shRNAs to ASRGL1 or scrambled shRNAs in mouse motor cortex. Images of brain sections stained with an ASRGL1 antibody to determine silencing efficiency. The shRNAs co-express RFP for tracking. RFF fluorescence appears more diffuse in ASRGL1-silenced brains probably due to the death of neurons caused by the treatment.

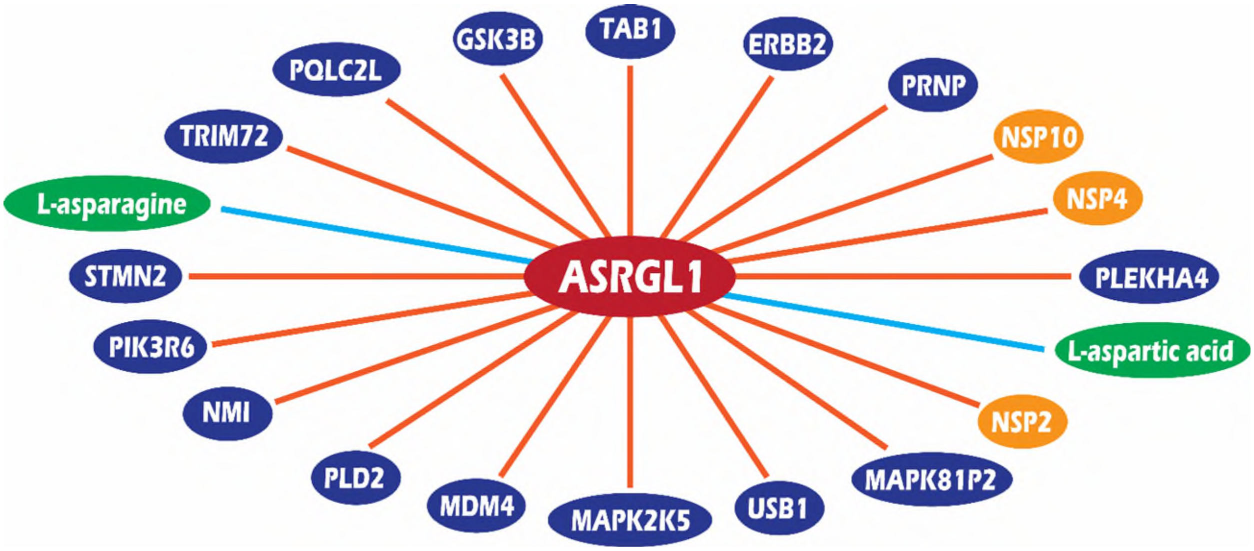

Supplementary Fig. 14: ASRGL1 interacts with other proteins associated with ALS. Curated bibliographic search through BioGRID (https://thebiogrid.org/) produced the previously published ASRGL1 interactome. Blue ellipses represent human proteins, orange ellipses represent viral proteins and green ellipses represent amino acids.

Supplementary Table 1: Demographic data of motor cortex samples analyzed by RNAseq (New York Genome Center (NYGC) ALS Consortium Database).

	**Controls**	**ALS**
**n**	68	323
**Males**	45	173
**Females**	23	150
**Age (years)**	63 ± 16	64 ± 10

Supplementary Table 2: Characteristics of brain samples

	**Frozen tissue**		**Formalin-fixed paraffin-embedded tissue**			
	**Controls**	**Sporadic ALS**	**Controls**	**Multiple sclerosis**	**Alzheimer's disease**	**Sporadic ALS**
**Sex**	10 M/10 F	10 M/10 F	2 M/2 F	2 M/2 F	2 M/2 F	2 M/2 F
**Age (years)**	63 (42-83)	63 (44-83)	59 (35-76)	74 (64-82)	75 (59-91)	63 (43-70)
**Region**	BA6 (pre-motor cortex)	BA6 (pre-motor cortex)	BA6 (pre-motor cortex)	BA6 (pre-motor cortex)	BA6 (pre-motor cortex)	BA6 (pre-motor cortex) and BA17 (visual cortex)
**RIN**	7.58 (7-8.8)	7.73 (7.1-9.6)	-	-		-
**PMI (hrs)**	15 (12-23)	16 (11-25)	26 (18-35)	18 (8-33)	17 (7-21)	24 (15-33)
**Institution**	NIH Neurobiobank	NIH Neurobiobank	NIH Neurobiobank	NIH Neurobiobank	NIH Neurobiobank	NIH Neuobiobank
	University of Maryland Brain and Tissue Bank			University of Maryland Brain and Tissue Bank		Harvard Brain Tissue Resource CenterUniversity of Pittsburg
	Harvard Brain Tissue Resource Center	University of Maryland Brain and Tissue Bank	Harvard Brain Tissue Resource	Harvard Brain Tissue Resource Center	Harvard Brain Tissue Resource Center	University of Pittsburg
	Brain Tissue Donation Program at the University of PittsburghNIH Neurobiobank	University of Miami Brain Endowment Bank				

M=Male; F=Female; BA= Brodmann's Area; RIN=RNA integrity number; PMI= postmortem interval

Supplementary Table 3: Demographic data of the donors of cells used for generating induced pluripotent stem cell lines to differentiate into motor neurons.

	Controls	Sporadic ALS
**n**	6	6
**Males**	4	4
**Females**	2	2
**Age (years)**	50.25 ± 7.5	53.14 ± 3

Supplementary Table 4: Antibodies used throughout the study.

Antibody	Vendor Source	Product #	Host Ig class	Clone	Assay
**ASRGL1**	Atlas	HPA055572	Rabbit IgG	Polyclonal	WB/ IHC/IF/PLA
**ASRGL1**	Atlas	HPA029725	Rabbit IgG	Polyclonal	WB
**TDP-43**	Encor	MCA-3H8	Mouse IgG1	MCA-3H8	IF mice
**TDP-43 C-terminal**	Cell Signaling	3448	Rabbit IgG	Polyclonal	WB/ IP
**TDP-43**	Proteintech	66734-1-Ig	Mouse IgG1	Polyclonal	PLA
**Phospho-TDP43**	Millipore	MABN14	Rat IgG2a	1D3	WB
**Ubiquitin**	Cell signaling	3933	Rabbit IgG	Polyclonal	WB
**UNC13A**	Proteintech	55053-1-AP	Rabbit IgG	Polyclonal	WB
**XBP1**	Abcam	Ab37152	Rabbit IgG	Polyclonal	WB
**NeuN**	Millipore Sigma	MAB377	Mouse IgG	A60	IF
**CTIP2**	Abcam	ab18465	Rat IgG	25B6	IF mice
**Anti-rat-HRP**	Thermofisher	31470	Goat IgG	Polyclonal	WB
**Anti-mouse-HRP secondary**	Cell Signaling	7076	Horse IgG	Polyclonal	WB
**Anti-rabbit-HRP secondary**	Cell Signaling	7074	Goat IgG	Polyclonal	WB
**Anti-mouse Alexa-fluor-488**	Thermofisher	A28175	Goat IgG	Polyclonal	IF
**Anti-mouse Alexa-fluor-594**	Thermofisher	A-11005	Goat IgG	Polyclonal	IF
**Anti-rabbit Alexa-fluor-488**	Thermofisher	A-11008	Goat IgG	Polyclonal	IF
**Anti-rabbit Alexa-fluor-594**	Thermofisher	A-11012	Goat IgG	Polyclonal	IF
**Anti-rat Alexa-fluor-633**	Thermofisher	A-21094	Goat IgG	Polyclonal	IF

Supplementary Table 5: Sequences of shRNAs against human and murine ASRGL1.

	shRNA	Sequence
Human	A	TACGCAACCTCCACAGGCGGTATCGTTAA
	B	CCTGTTCCACATAGAACAAGGAAAGACGG
	C	CAATGACATCGGAGCCGTCTCAACCACAG
	D	ACGGTAGAAGAGGCTGCGGACCTATCGTT
Mouse	A	TGCAGTGTCTGCCGTGCGCTGTATCGCAA
	B	ATGGTGGCGGAGCAAGCAACATCTCAGCC
	C	CGGCATTGACCTCTGTGAGACCAGGACAA
	D	AGGAAACTTGGCTTACGCAACCTCTACTG
