# Supplementary material for: TDP-43 proteinopathy in ALS is triggered by loss of ASRGL1 and associated with HML-2 expression
Source: Nat Commun. 2024 May 16;15:4163. doi: 10.1038/s41467-024-48488-7 (PMC11099023; doi:10.1038/s41467-024-48488-7)
Supplement: Supplementary file 3 — Description of Additional Supplementary Files [file 41467_2024_48488_MOESM3_ESM.pdf]

Description of Additional Supplementary Files

File Name: Supplementary Data 1
Description: Structural analysis of isoaspartate formation in TDP-43.

File Name: Supplementary Movie 1
Description: Interaction of TDP-43 and ASRGL1 in the perinuclear region.
