# Supplementary material for: TDP-43 proteinopathy in ALS is triggered by loss of ASRGL1 and associated with HML-2 expression
Source: Nat Commun. 2024 May 16;15:4163. doi: 10.1038/s41467-024-48488-7 (PMC11099023; doi:10.1038/s41467-024-48488-7)
Supplement: Supplementary file 4 — Supplementary Data 1 [file 41467_2024_48488_MOESM4_ESM.pdf]

```
>sp|Q13148|TADBP_HUMAN TAR DNA-binding protein 43 OS=Homo sapiens OX=9606
GN=TARDBP PE=1 SV=1
MSEYIRVTEDENDEPIEIPSEDDGTVLLSTVTAQFPGACGLRYRNPVSQCMRGVRLVEGILHAPDAGWGNLVYVVNY
PKDNKRKMDETDASSAVKVKRAVQKTSDLIVLGLPWKTTEQDLKEYFSTFGEVLMVQVKKDLKTGHSKGFGFVRFTE
YETQVKVMSQRHMIDGRWCDCKLPNSKQSQDEPLRSRKVFVGRCTEDMTEDELREFFSQYGDVMDVFIPKPFRAFAF
VTFADDQIAQSLCGEDLIIKGISVHISNAEPKHNSNRQLERSGRFGGNPGGFGNQGGFGNSRGGGAGLGNNQGSNMG
GGMNFGAFSINPAMMAAAQAALQSSWGMMGMLASQQNQSGPSGNNQNQGNMQREPNQAFGSGNNSYSGSNSGAAIGW
GSASNAGSGSGFNGGFGSSMDSKSSGWGM
```

5MRG, 4BS2, 6N3C, 6N3A, 5WIA, 5WIQ

```
MSEYIRVTEDENDEPIEIPSEDDGTVLLSTVTAQFPGACGLRYRNPVSQCMRGVRLVEGILH
APDAGWGNLVYVVNYPKDNKRKMDETDASSAVKVKRAVQKTSDLIVLGLPWKTTEQDLKEYF
STFGEVLMVQVKKDLKTGHSKGFGFVRFTEYETQVKVMSQRHMIDGRWCDCKLPNSKQSQDE
PLRSRKVFVGRCTEDMTEDELREFFSQYGDVMDVFIPKPFRAFAFVTFADDQIAQSLCGEDL
IIKGISVHISNAEPKHNSNRQLERSGRFGGNPGGFGNQGGFGNSRGGGAGLGNNQGSNMGGG
MNFGAFSINPAMMAAAQAALQSSWGMMGMLASQQNQSGPSGNNQNQGNMQREPNQAFGSGNN
SYSGSNSGAAIGWGSASNAGSGSGFNGGFGSSMDSKSSGWGM
```

MSEYIRVTEDE**ND**EPIEIPSEDDGTVLLSTVTAQ
FPGACGLRYRNPVSQCMRGVRLVEGILHAPDAGW
GNLVYVVNYPKDNKRKMDETDASSAVKVKRAVQ

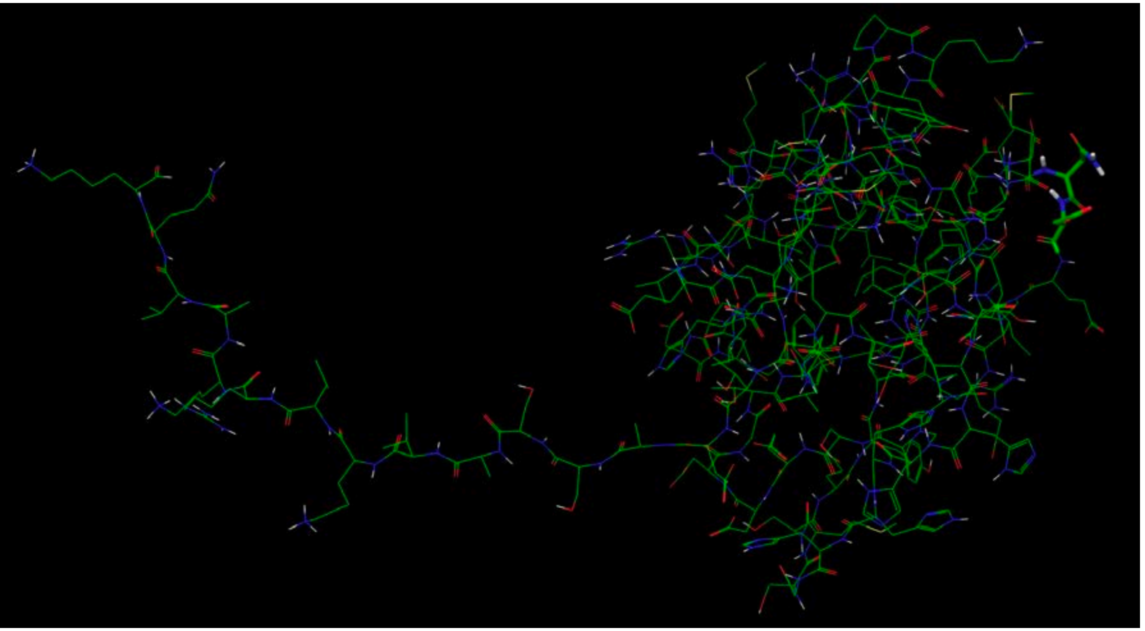

5MRG: -4021.2

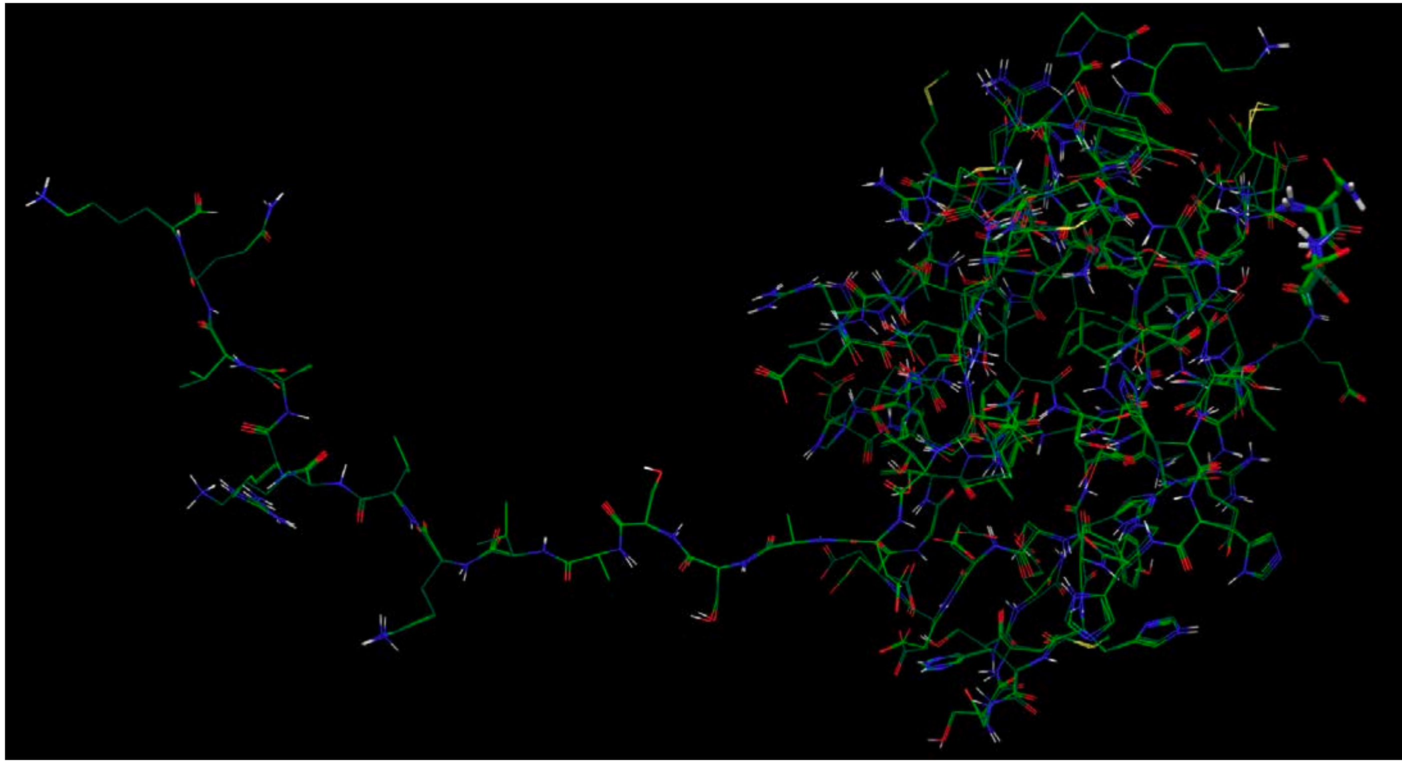

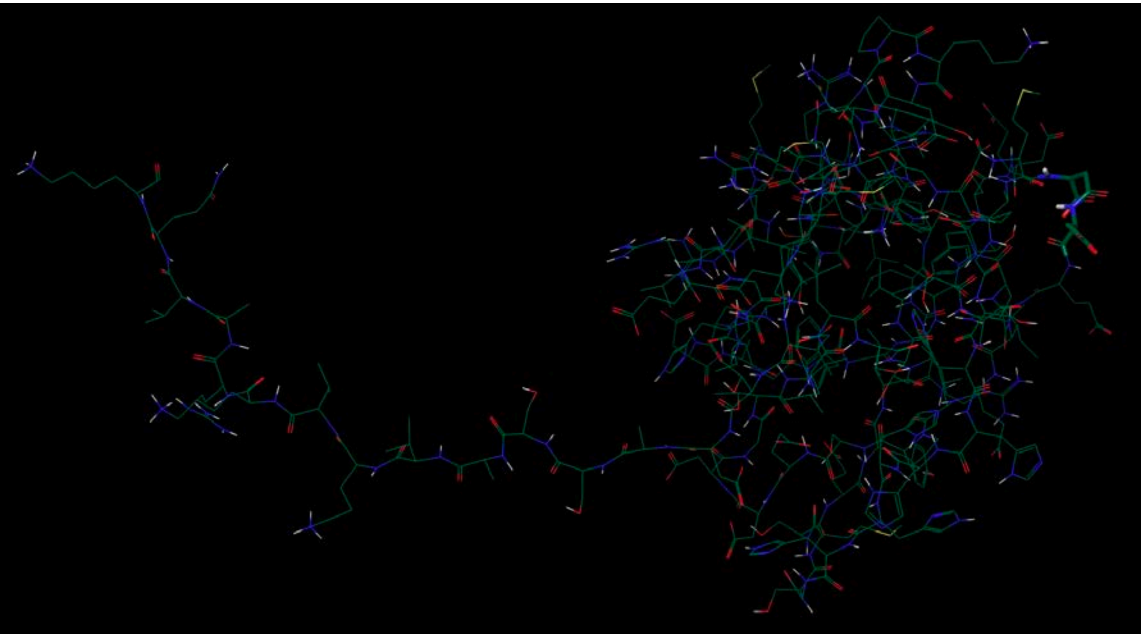

5MRG with isoaspartate24: -3997.5

MSEYIRVTEDE**ND**EPIEIPSEDDGTVLLSTVTAQ
FPGACGLRYRNPVSQCMRGVRLVEGILHAPDAGW
GNLVYYVVNYPKDNKRKMDETDASSAVKVKRAVQ

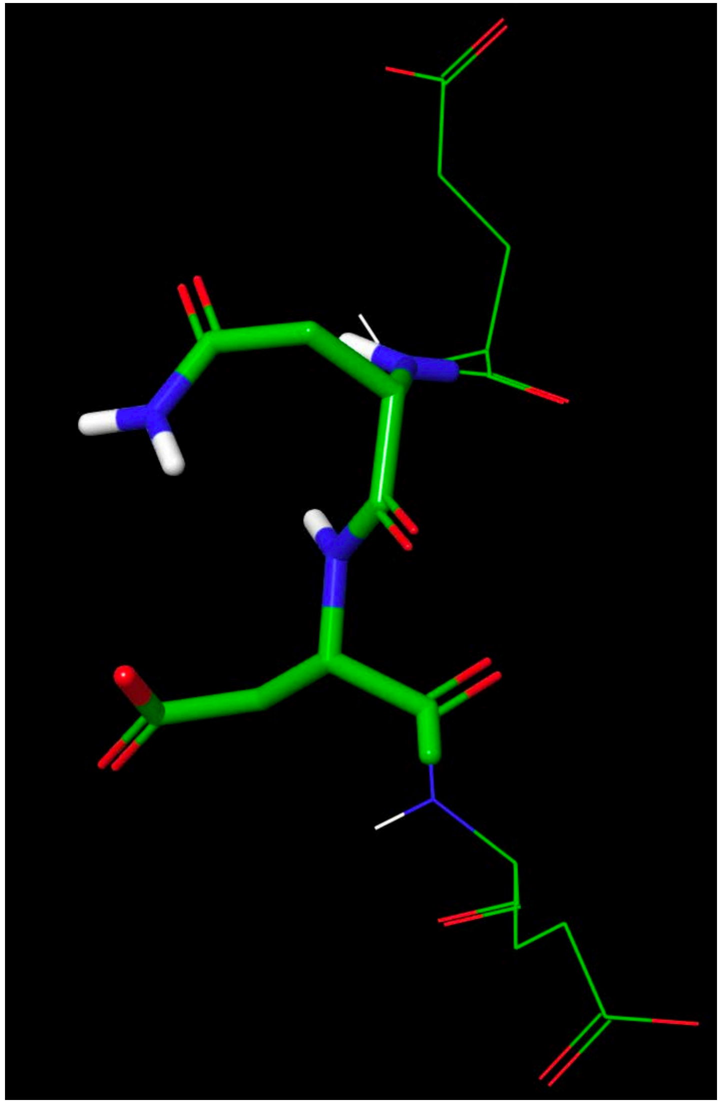

5MRG: -4021.2

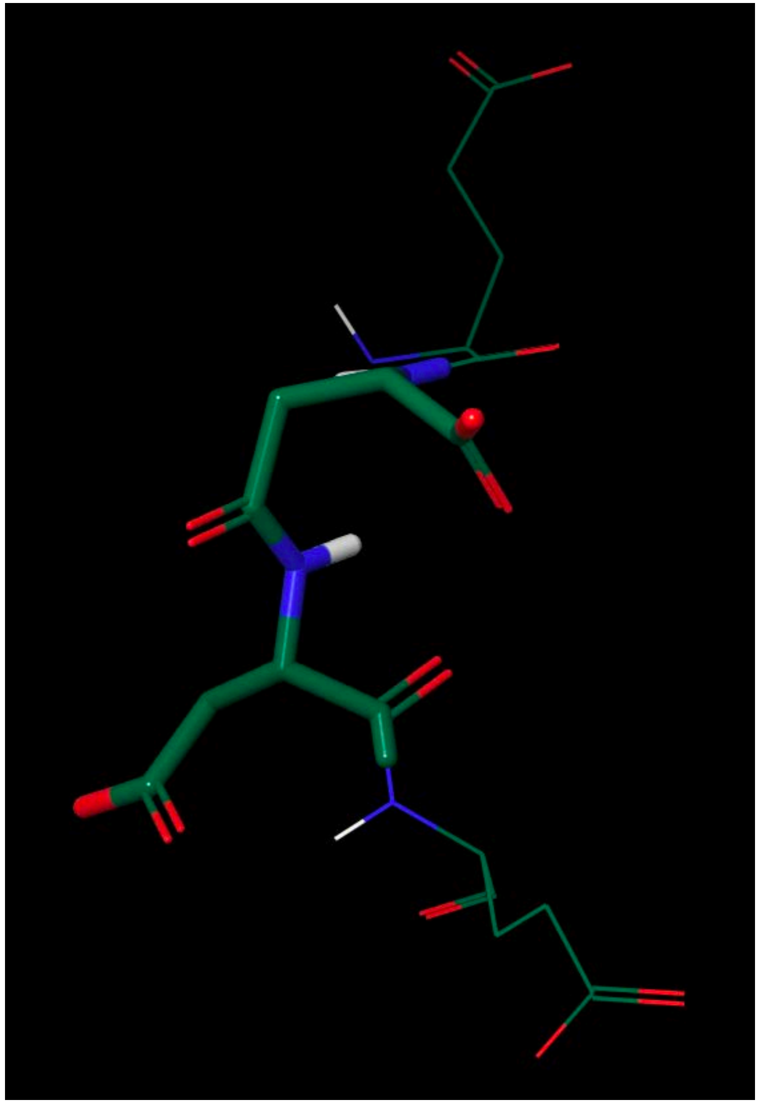

5MRG with isoaspartate24: -3997.5

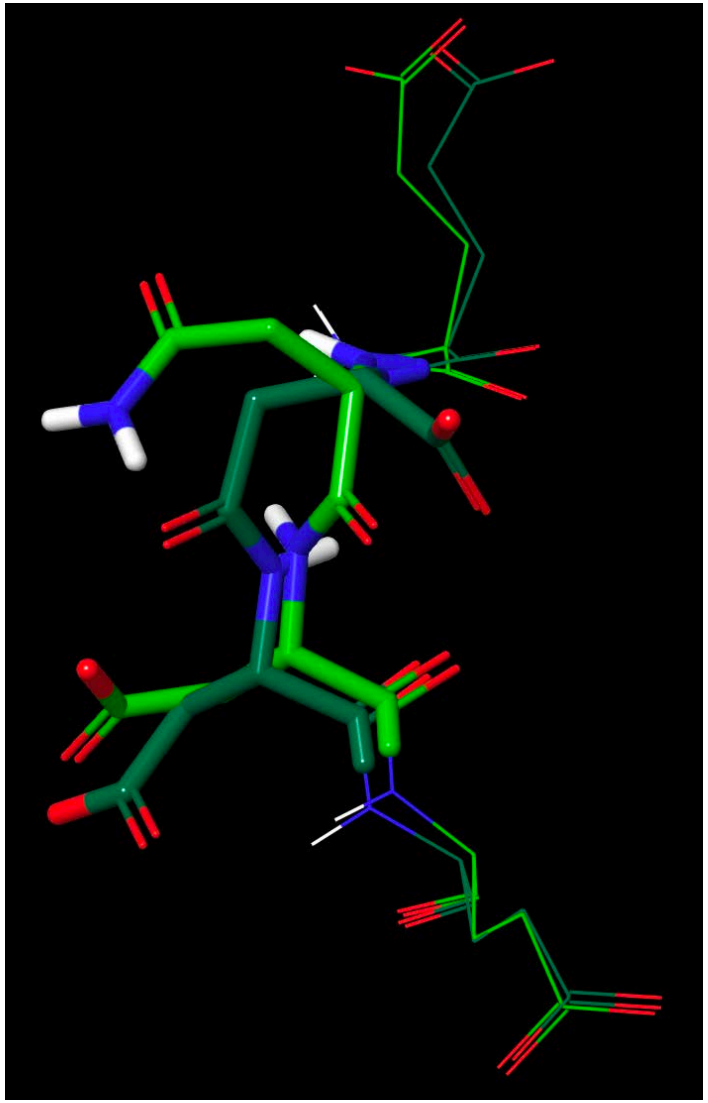

4BS2 all sites highlighted (179and265and259) : -8531.4 (RNA in yellow)

KTSDLIVLGLPWKTTEQDLKEYFSTFGE
VLMVQVKKDLKTGHSKGFGFVRFTEYET
QVKVMSQRHMIDGRWCDCKLP**NS**KQSQD
EPLRSRKVFVGRCTEDMTEDELREFFSQ
YGDVMDVFIPKPFRAFAFVTFADDQIAQ
SLCGEDLIIKGISVHIS**NA**EPKH**NS**NRQ

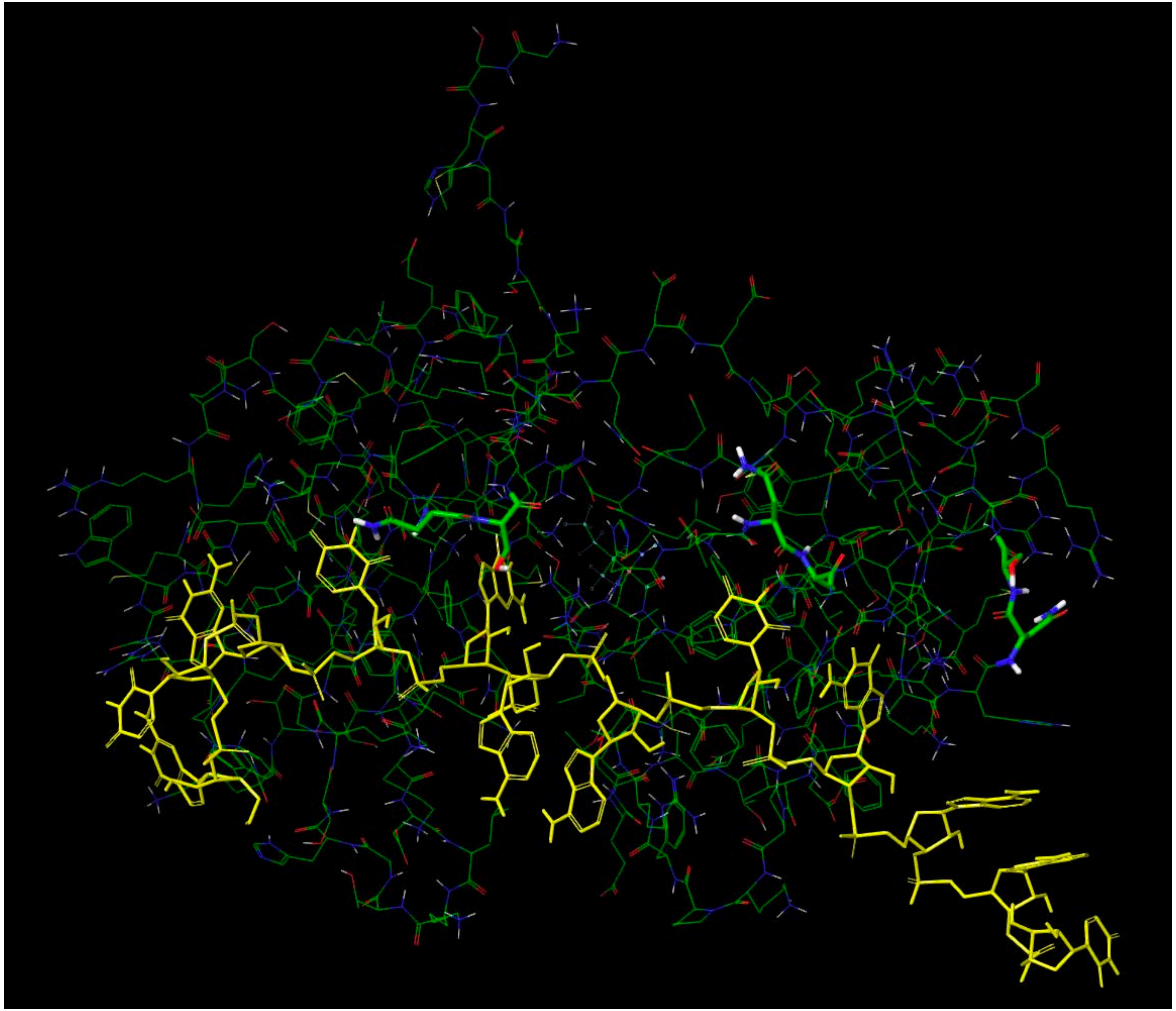

4BS2 Isoaspart_179and265and259: -8273.9

KTSDLIVLGLPWKTTEQDLKEYFSTFGE
VLMVQVKKDLKTGHSKGFGFVRFTEYET
QVKVMSQRHMIDGRWCDCKLPNSKQSQD
EPLRSRKVFVGRCTEDMTEDELREFFSQ
YGDVMDVFIPKPFRAFAFVTFADDQIAQ
SLCGEDLIIKGISVHISNAEPKHNSNRQ

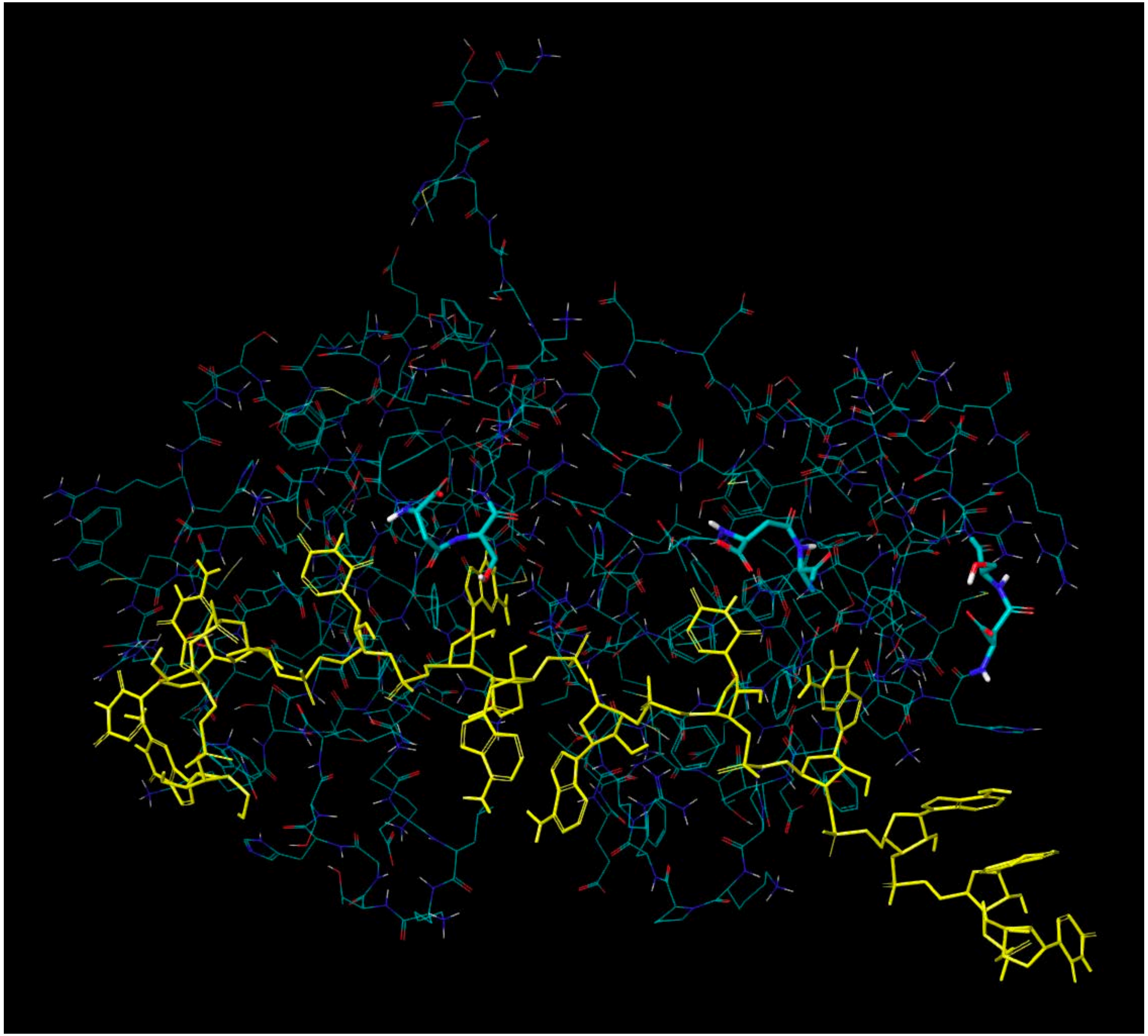

4BS2_179and265and259 : overlay

KTSDLIVLGLPWKTTEQDLKEYFSTFGE
VLMVQVKKDLKTGHSKGFGFVRFTEYET
QVKVMSQRHMIDGRWCDCKLP**NS**KQSQD
EPLRSRKVFVGRCTEDMTEDELREFFSQ
YGDVMDVFIPKPFRAFAFVTFADDQIAQ
SLCGEDLIIKGISVHIS**NA**EPKH**NS**NRQ

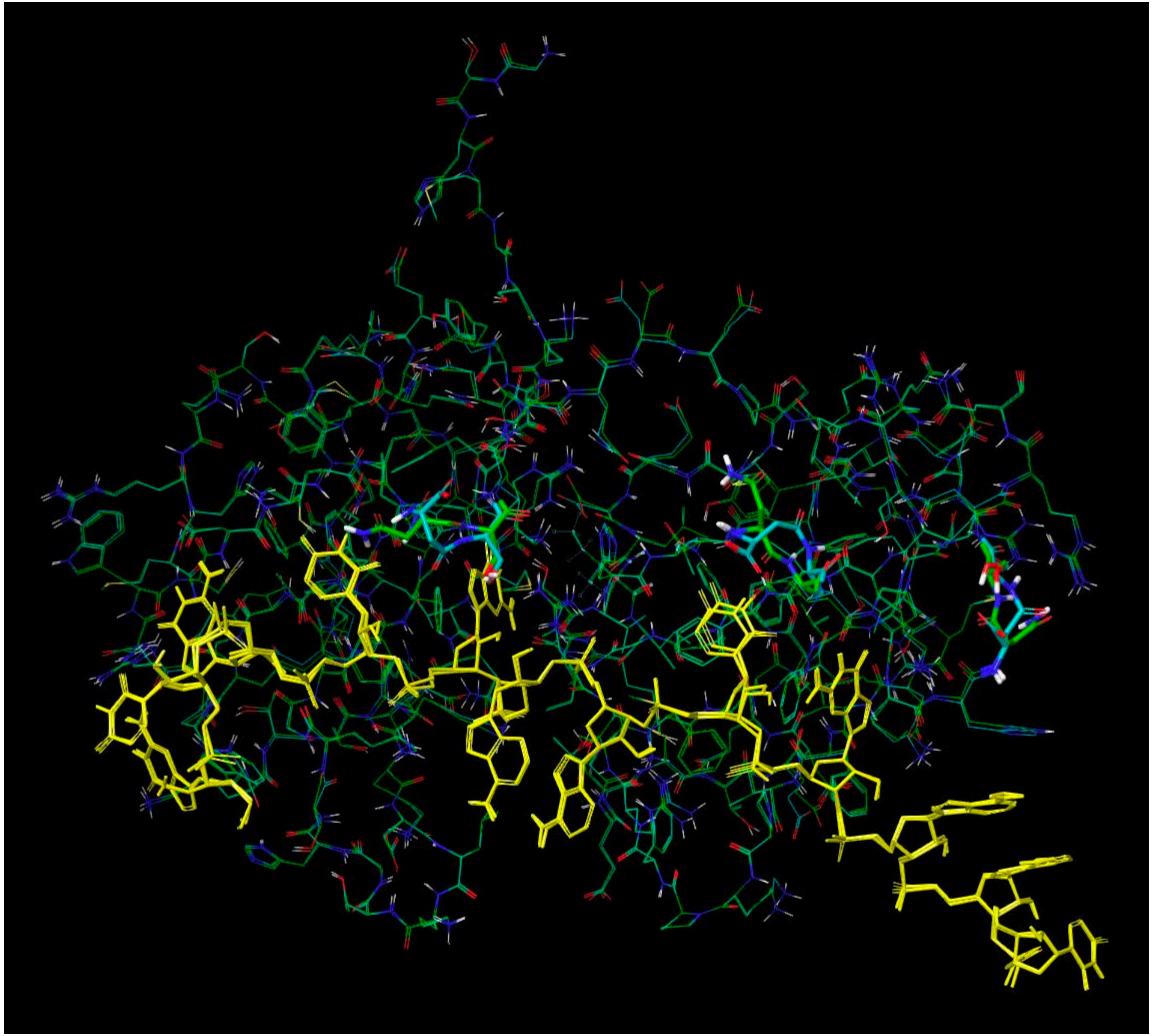

4BS2 Isoaspart_179: -8193.9 overlay

4BS2: -8531.4

KTSDLIVLGLPWKTTEQDLKEYFSTFGE
VLMVQVKKDLKTGHSKGFGFVRFTEYET
QVKVMSQRHMI**DG**RWCDCKLPNSKQSQD
EPLRSRKVFVGRCTEDMTEDELREFFSQ
YGDVMDVFIPKPFRAFAFVTFADDQIAQ
SLCGEDLIIKGISVHISNAEPKH**NS**NRQ

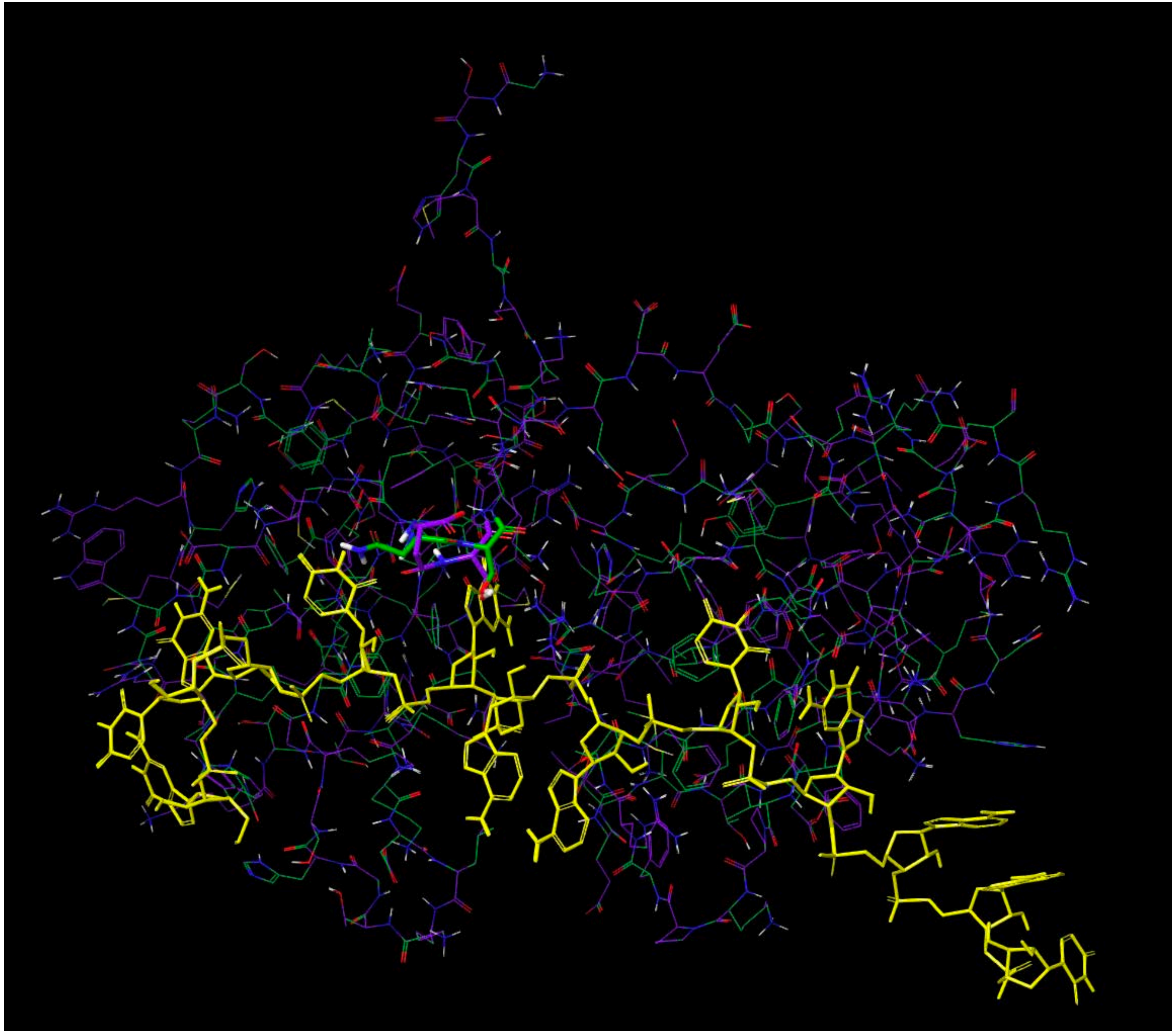

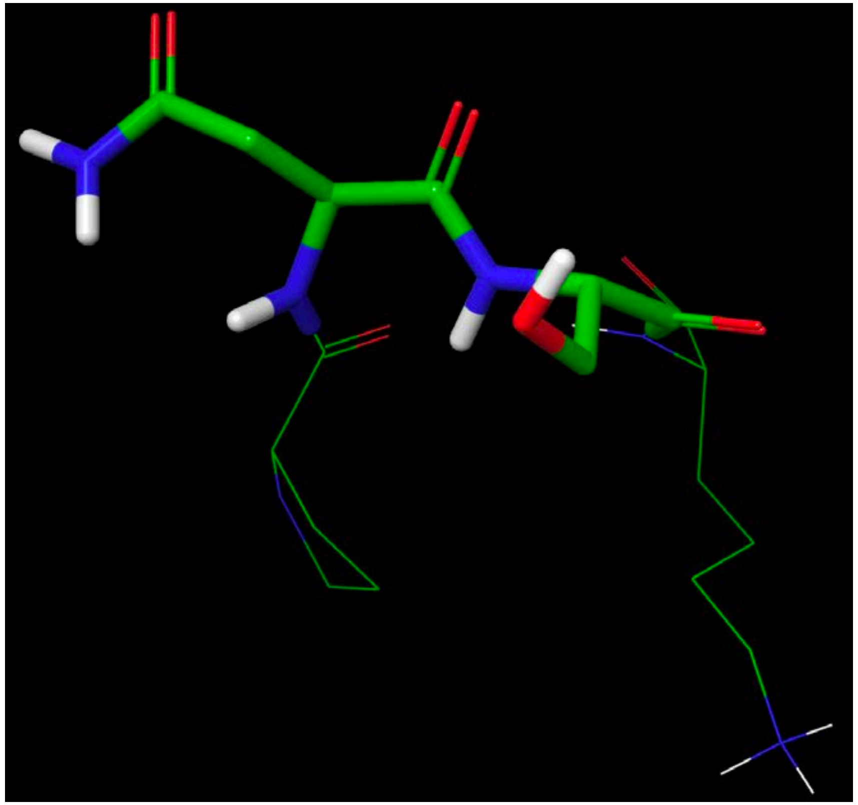

4BS2: -8531.4

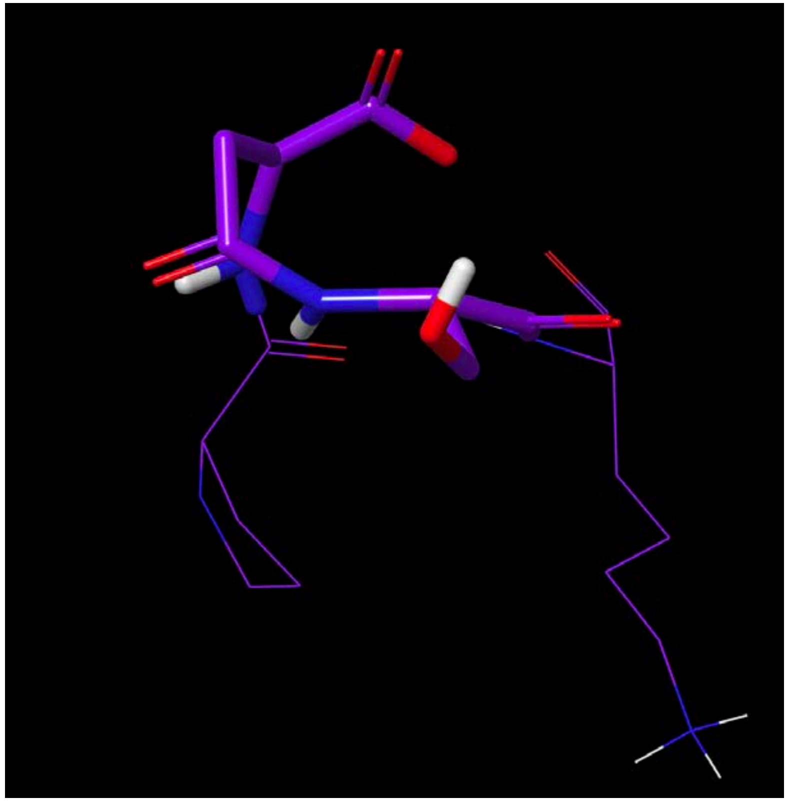

4BS2 Isoaspart_179: -8193.9

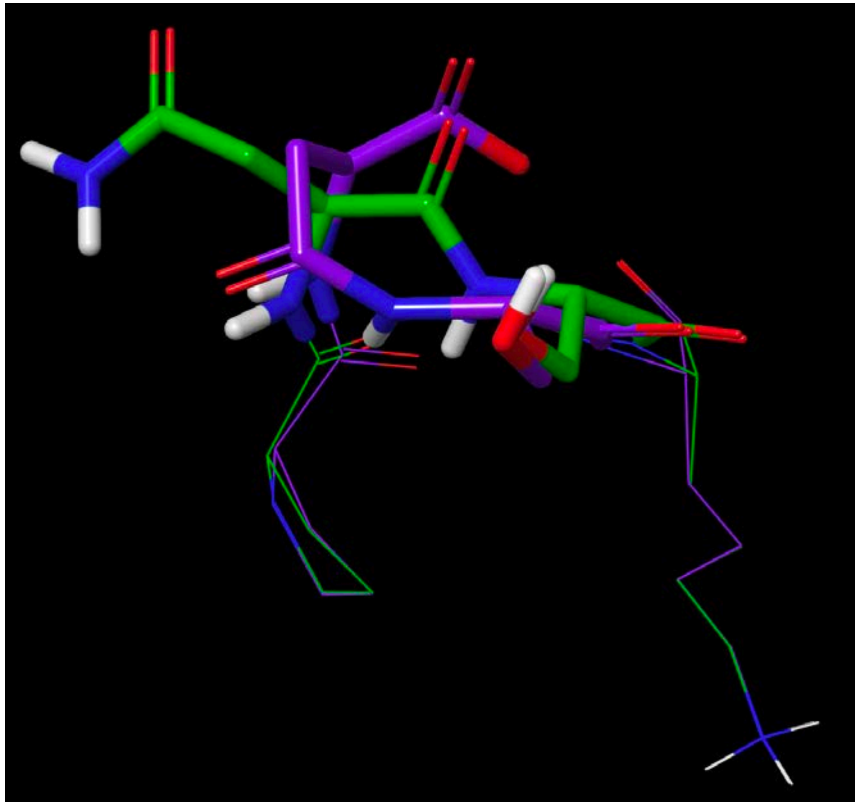

4BS2 isoaspart 259: -8438.4
overlay

4BS2: -8531.4

KTSDLIVLGLPWKTTEQDLKEYFSTFGE
VLMVQVKKDLKTGHSKGFGFVRFTEYET
QVKVMSQRHMIDGRWCDCKLP**NS**KQSQD
EPLRSRKVFVGRCTEDMTEDELREFFSQ
YGDVMDVFIPKPFRAFAFVTFADDQIAQ
SLCGEDLIIKGISVHIS**NA**EPKH**NS**NRQ

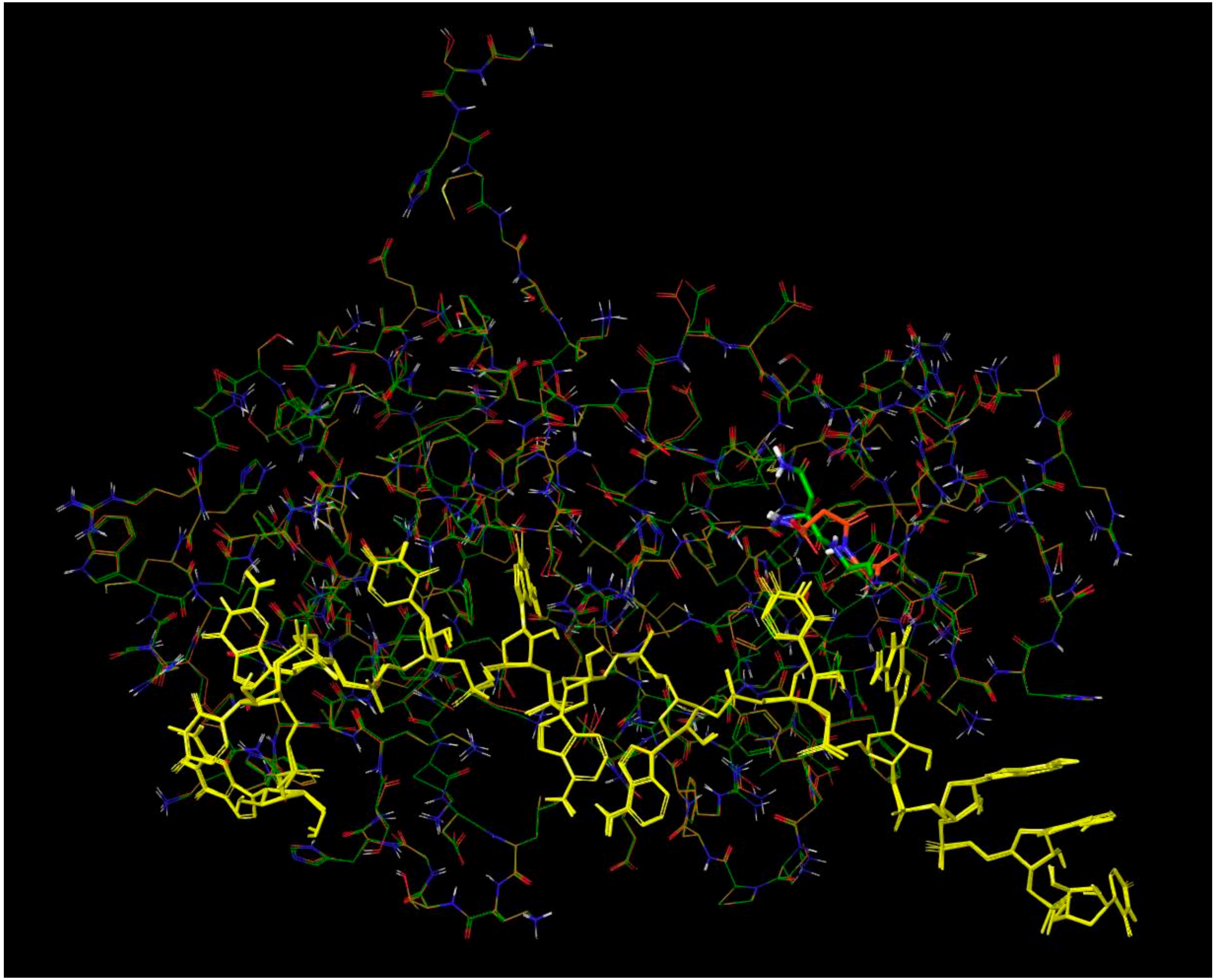

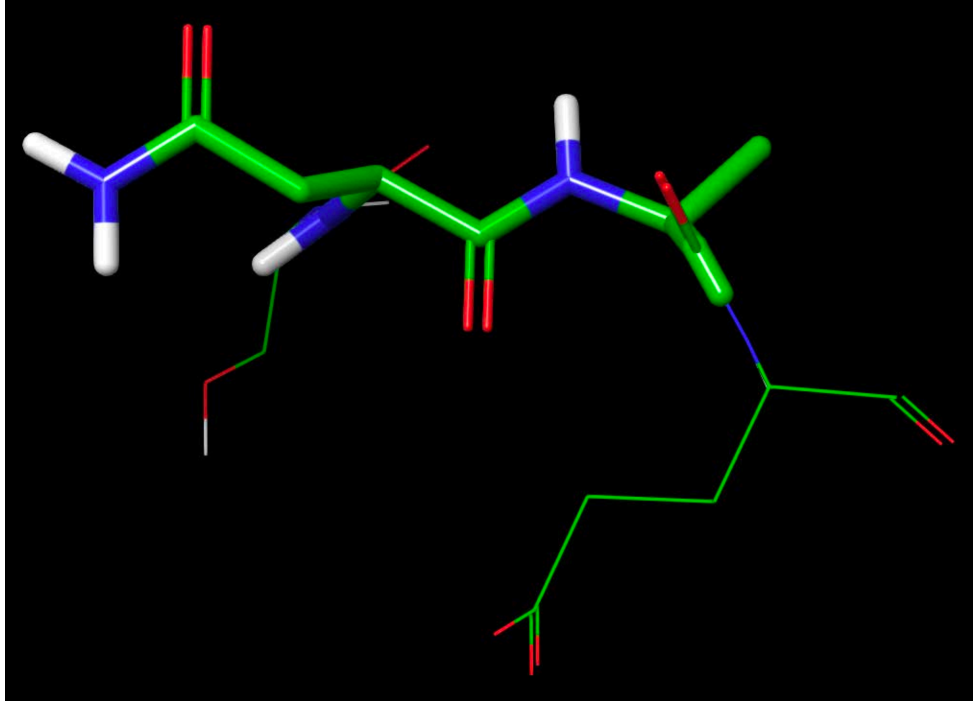

4BS2: -8531.4

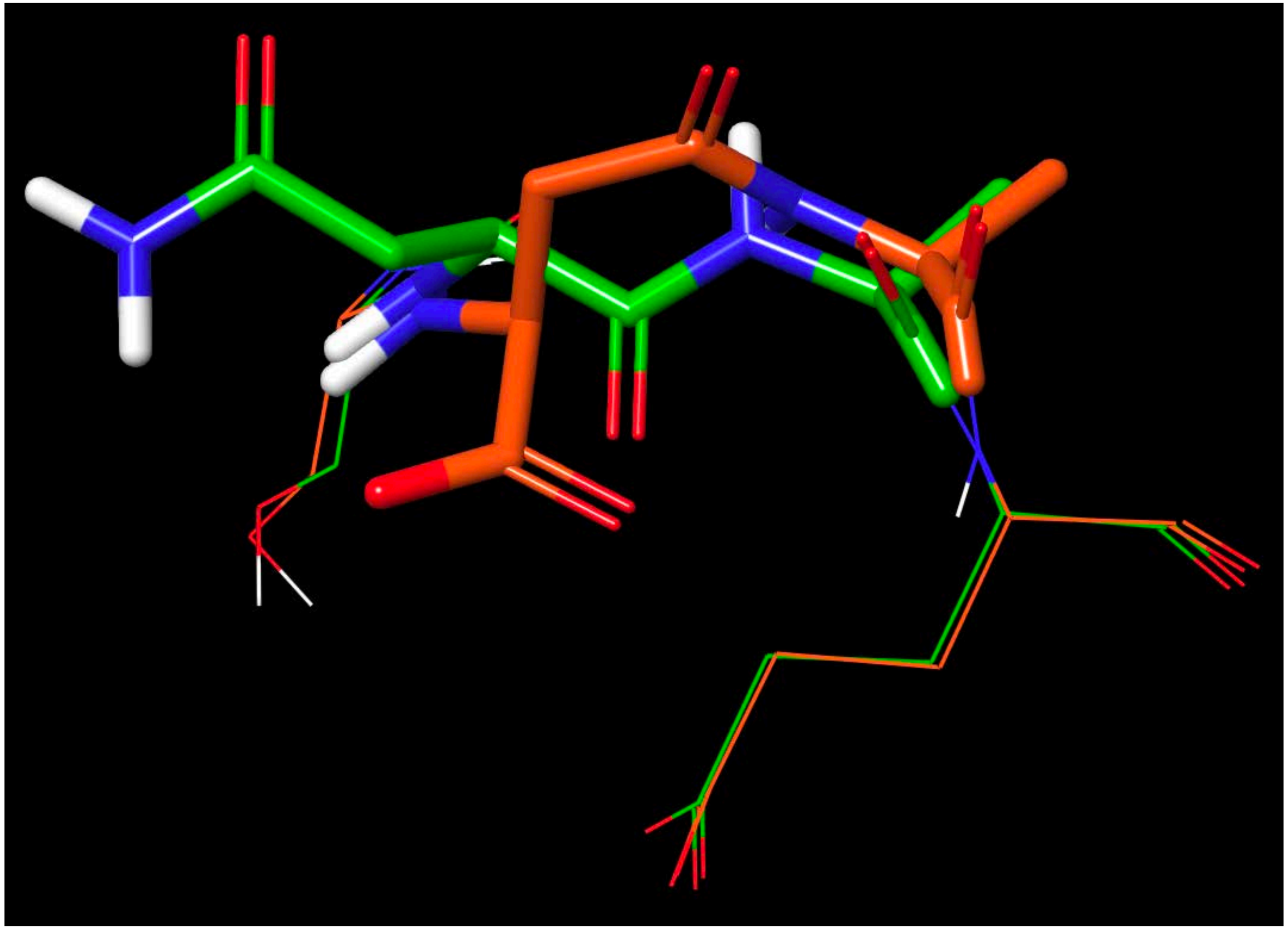

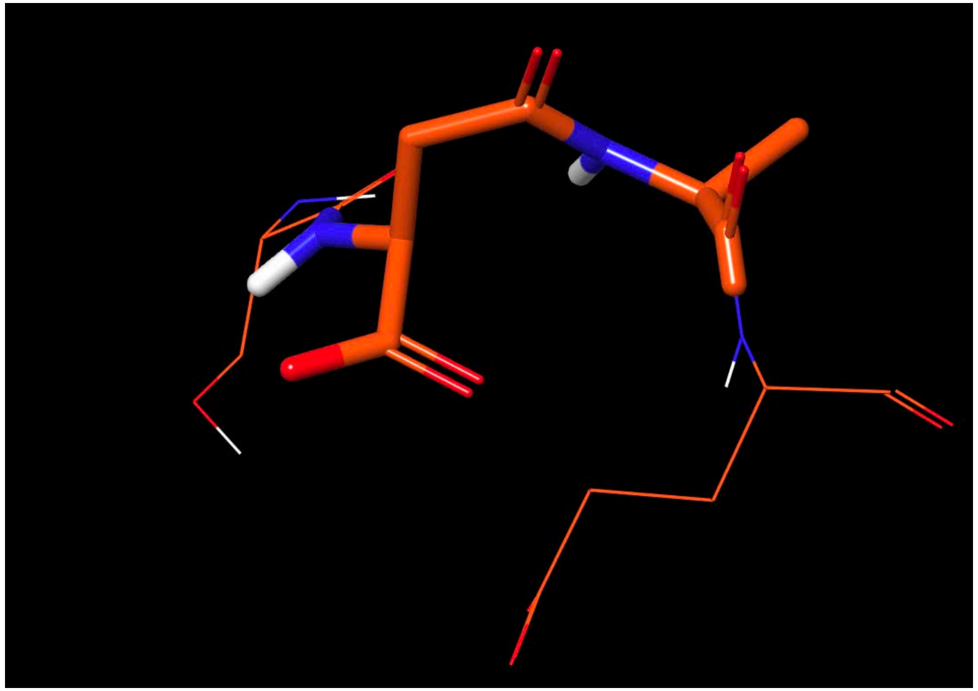

4BS2 isoaspart 259: -8438.4

4BS2 Isoaspart_265: -8439.7

Overlay

4BS2: -8531.4

KTSDLIVLGLPWKTTEQDLKEYFSTFGE
VLMVQVKKDLKTGHSKGFGFVRFTEYET
QVKVMSQRHMIDGRWCDCKLP**NS**KQSQD
EPLRSRKVFVGRCTEDMTEDELREFFSQ
YGDVMDVFIPKPFRAFAFVTFADDQIAQ
SLCGEDLIIKGISVHISNAEPKH**NS**NRQ

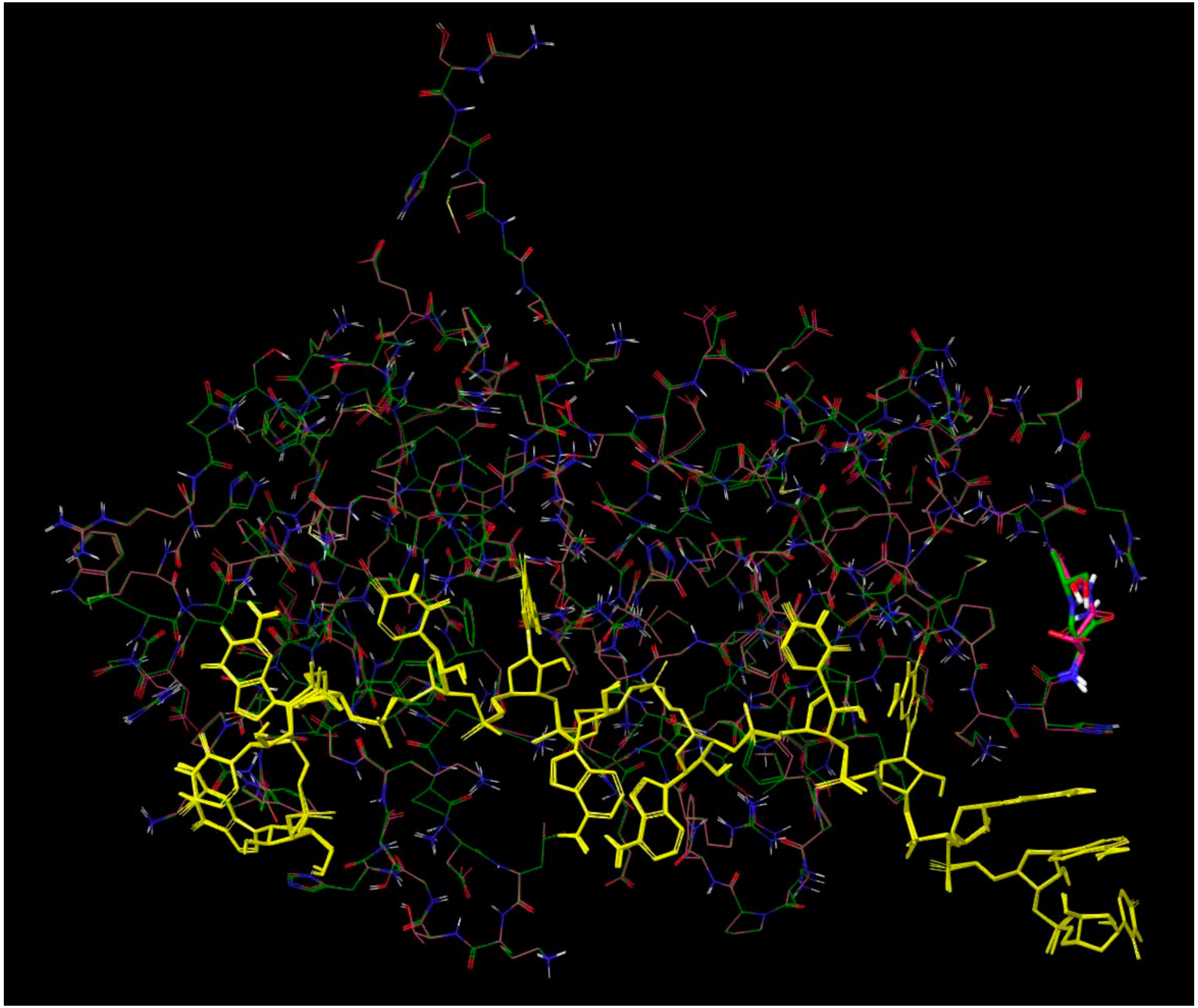

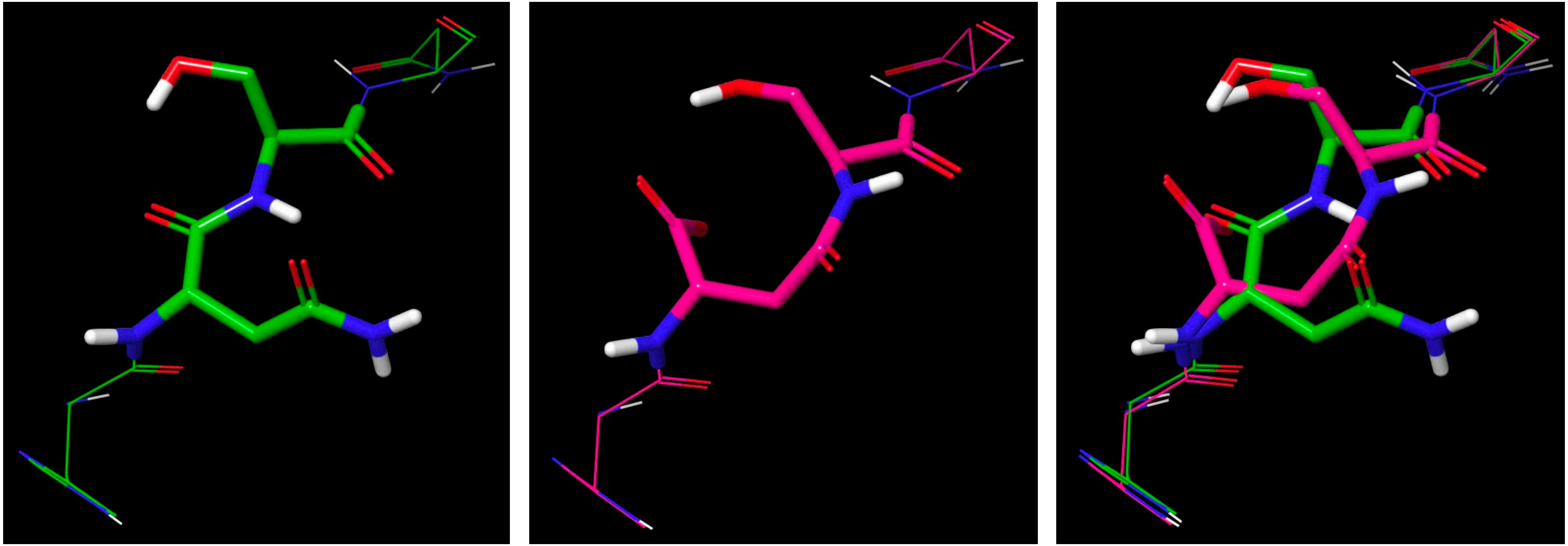

4BS2: -8531.4

4BS2 Isoaspart_265: -8439.7

4BS2 Isoaspart_179and265: -8290.6

KTSDLIVLGLPWKTTEQDLKEYFSTFGE
VLMVQVKKDLKTGHSKGFGFVRFTEYET
QVKVMSQRHMIDGRWCDCKLPNSKQSQD
EPLRSRKVFVGRCTEDMTEDELREFFSQ
YGDVMDVFIPKPFRAFAFVTFADDQIAQ
SLCGEDLIIKGISVHISNAEPKHNSNRQ

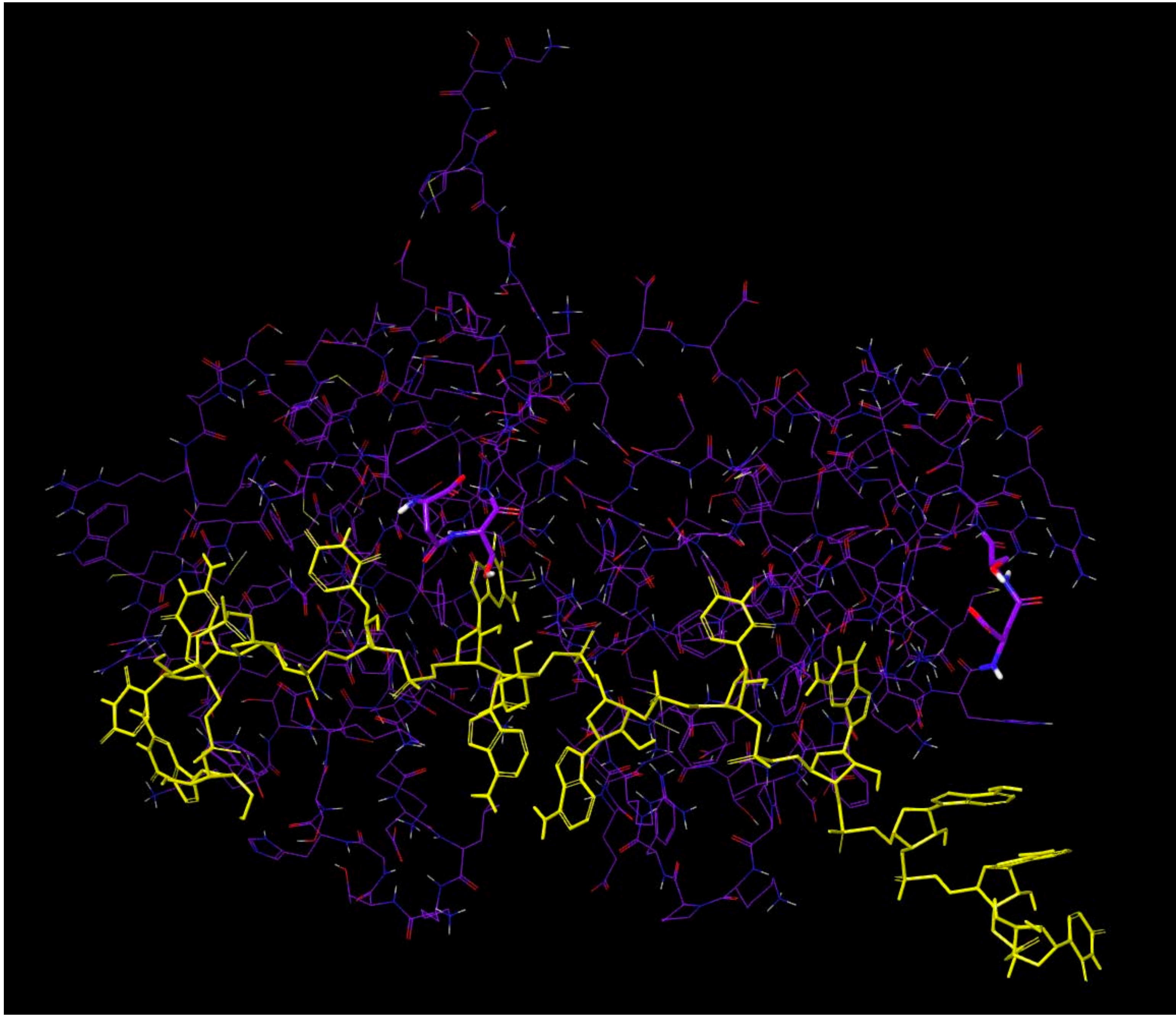

GFG**N̲S**RGGGAGLGN̲N̲QGSN̲MGGGMN̲FGEFSIN̲

6N3C: -1064.5

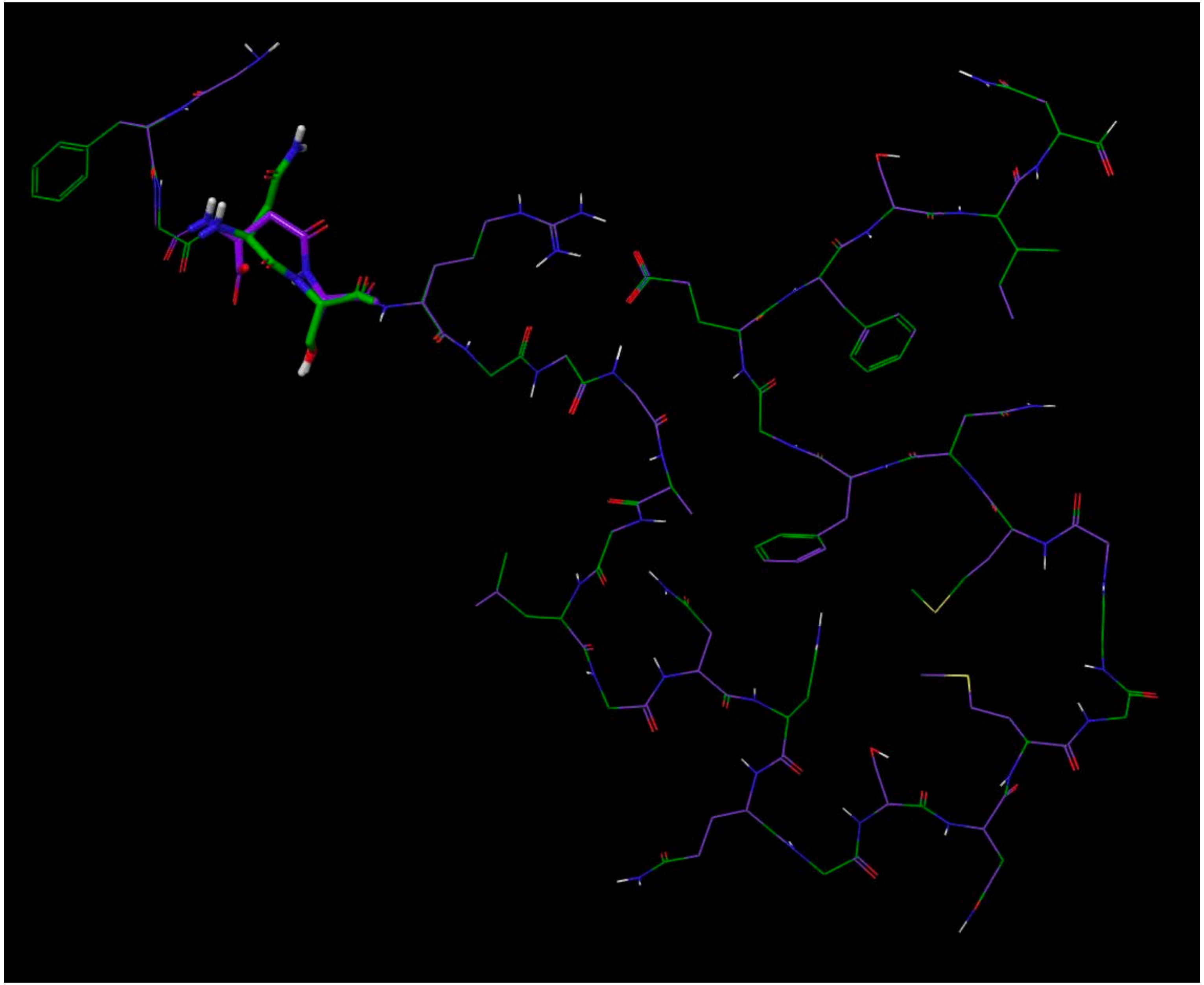

6N3C with isoaspartate 291: -902.6

GFGNSRGGGAGLGNNQGSNMGGGMNFGEFSIN

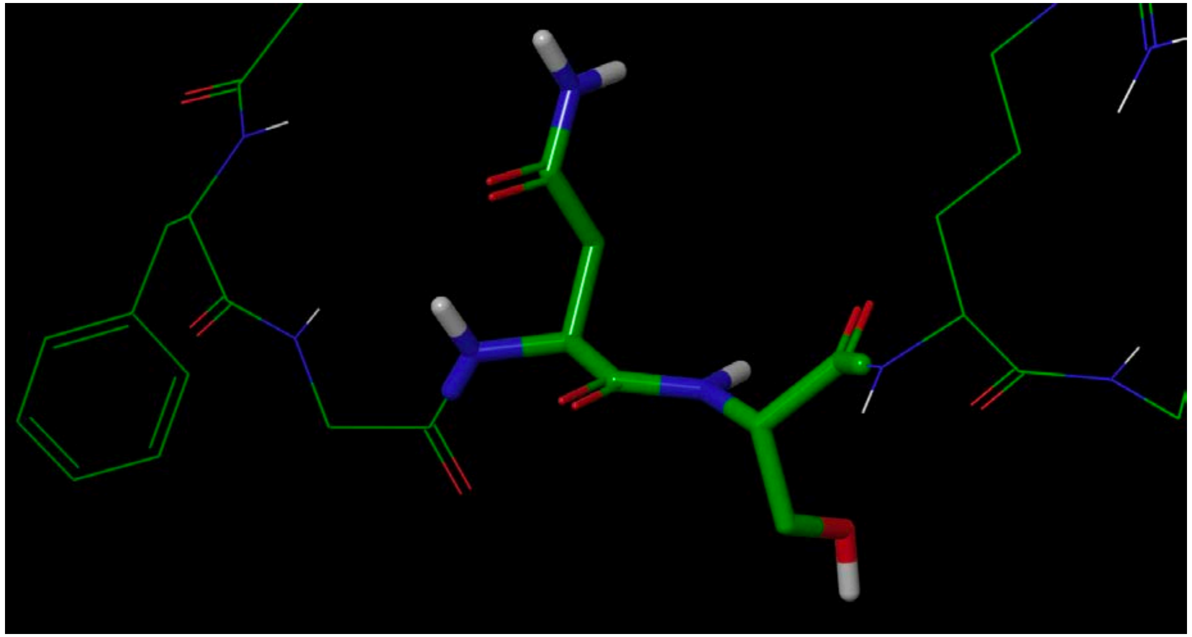

6N3C: -1064.5

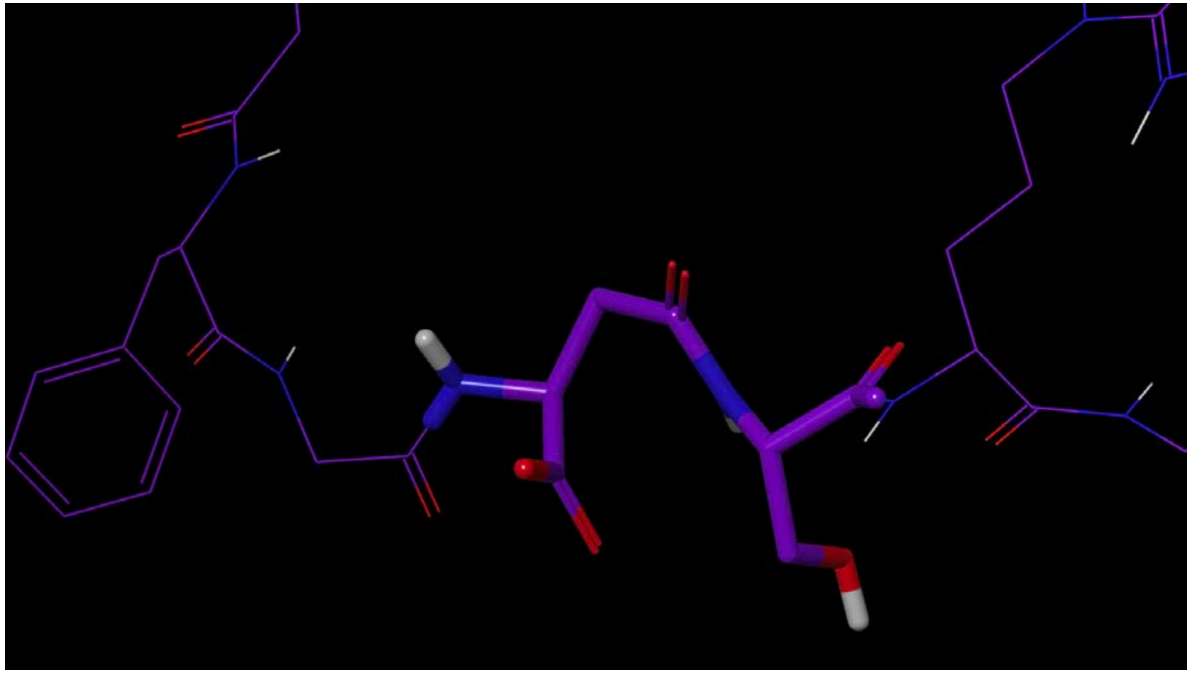

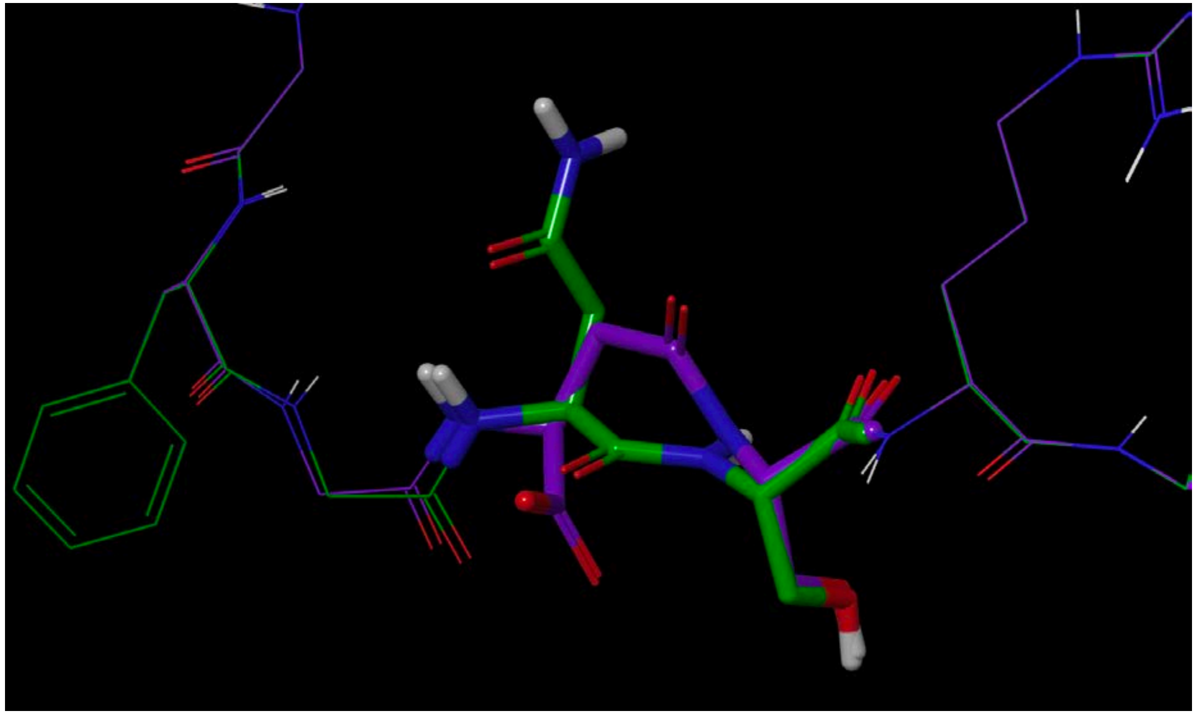

6N3C with isoaspartate 291: -902.6

GFGNSRGGGAGLG**NN**QGSNMGGMNFGEFSIN

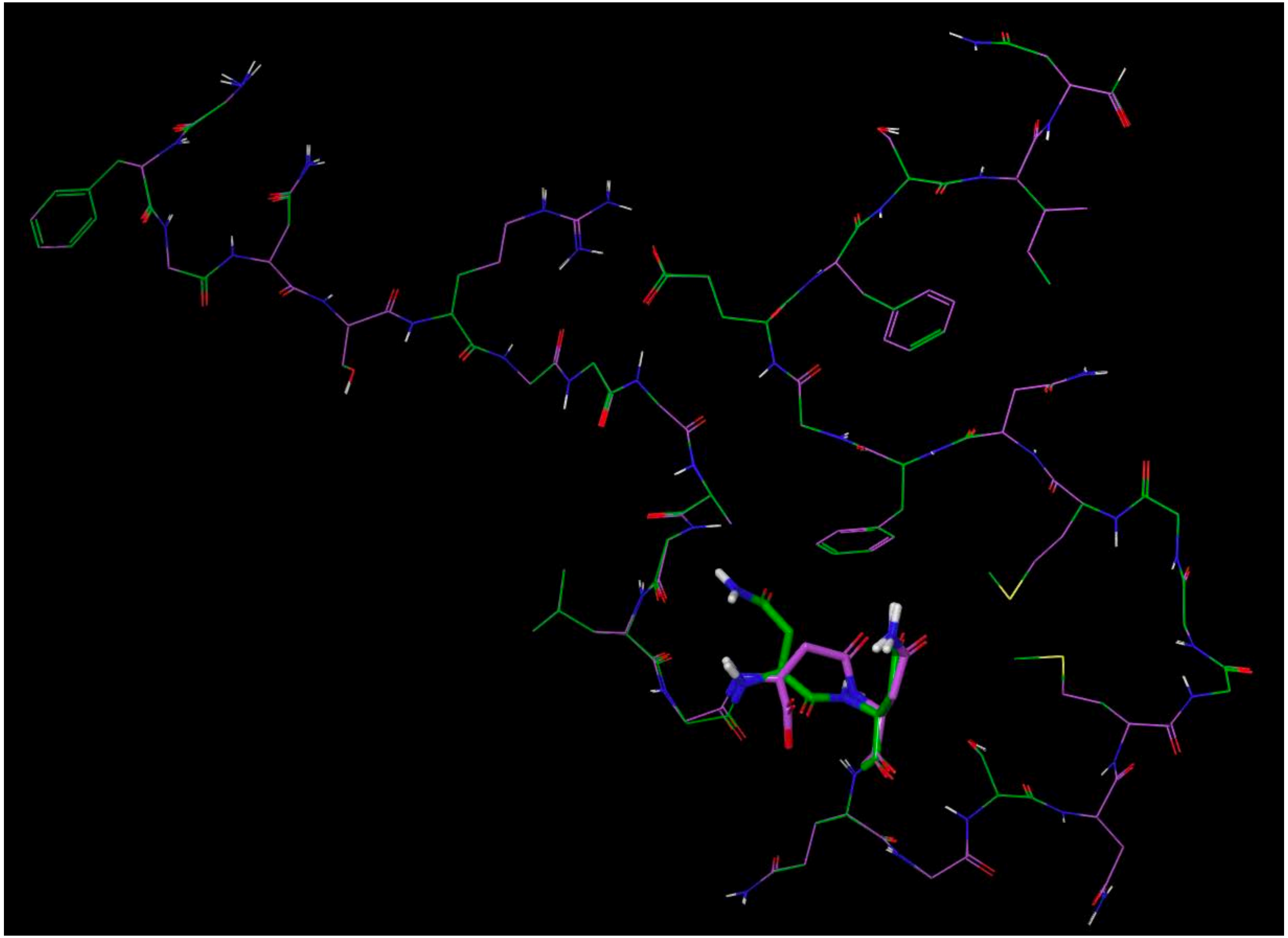

6N3C: -1064.5

6N3C with isoaspartate 301: -894.2

GFGNSRGGGAGLG**NN**QGSNMGGGMNFGEFSIN

6N3C: -1064.5

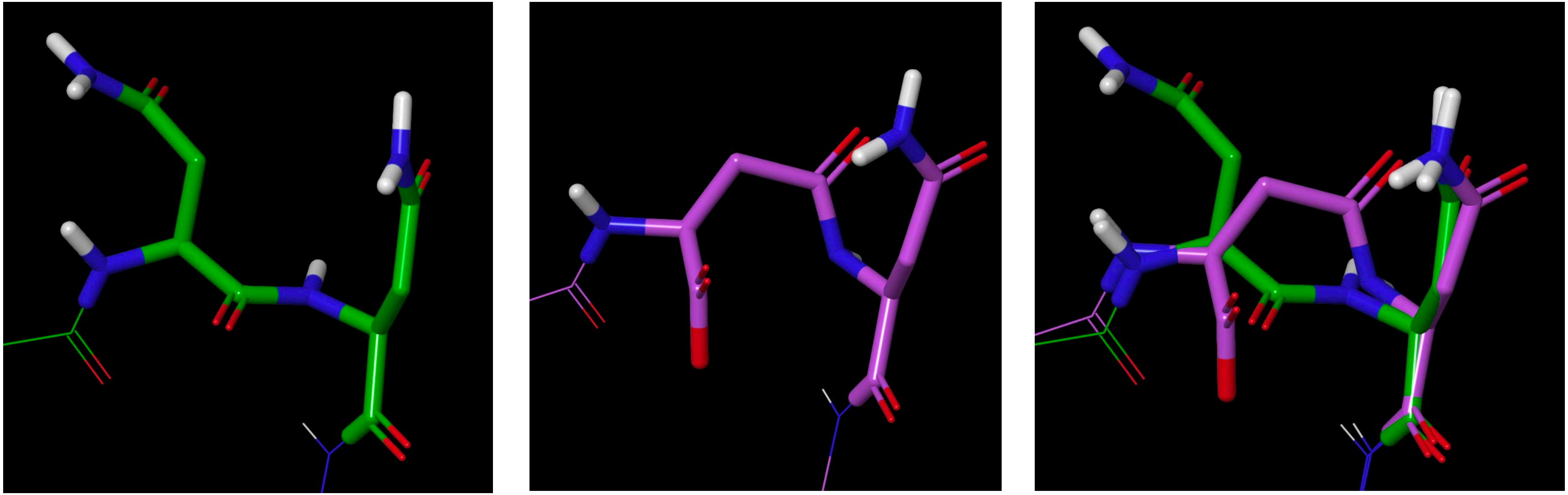

6N3C with isoaspartate 301: -894.2

GFGNSRGGGAGLGNNQGSNMGGGMNFGEFSIN

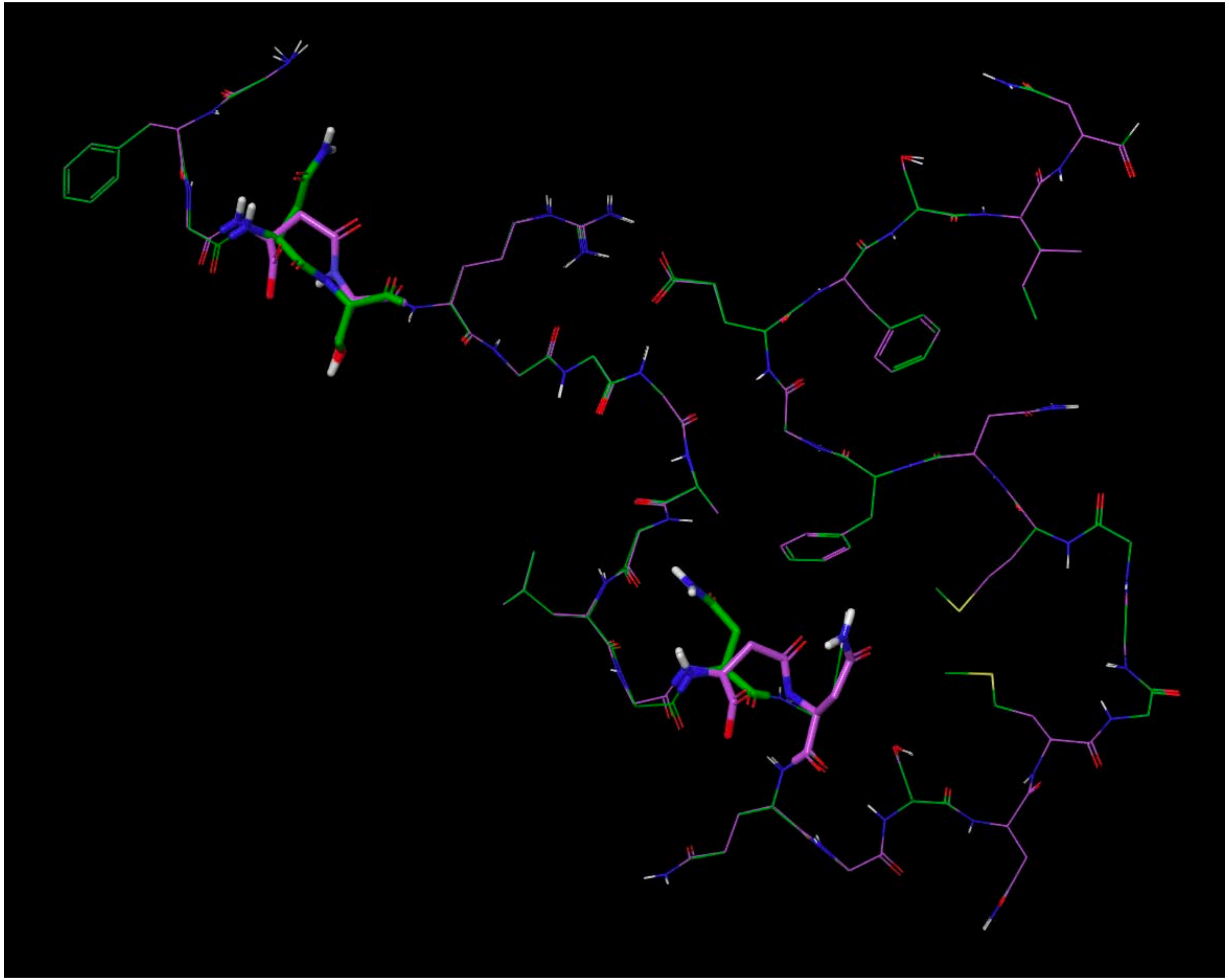

6N3C: -1064.5

6N3C with isoaspartates 291 and 301: -759.7

6N3A: -1778.6; all site highlighted

MNFGAFSINPAMMAAAQAALQSSWGMMGMLASQQNQSGPSGNNQNQGNMQ

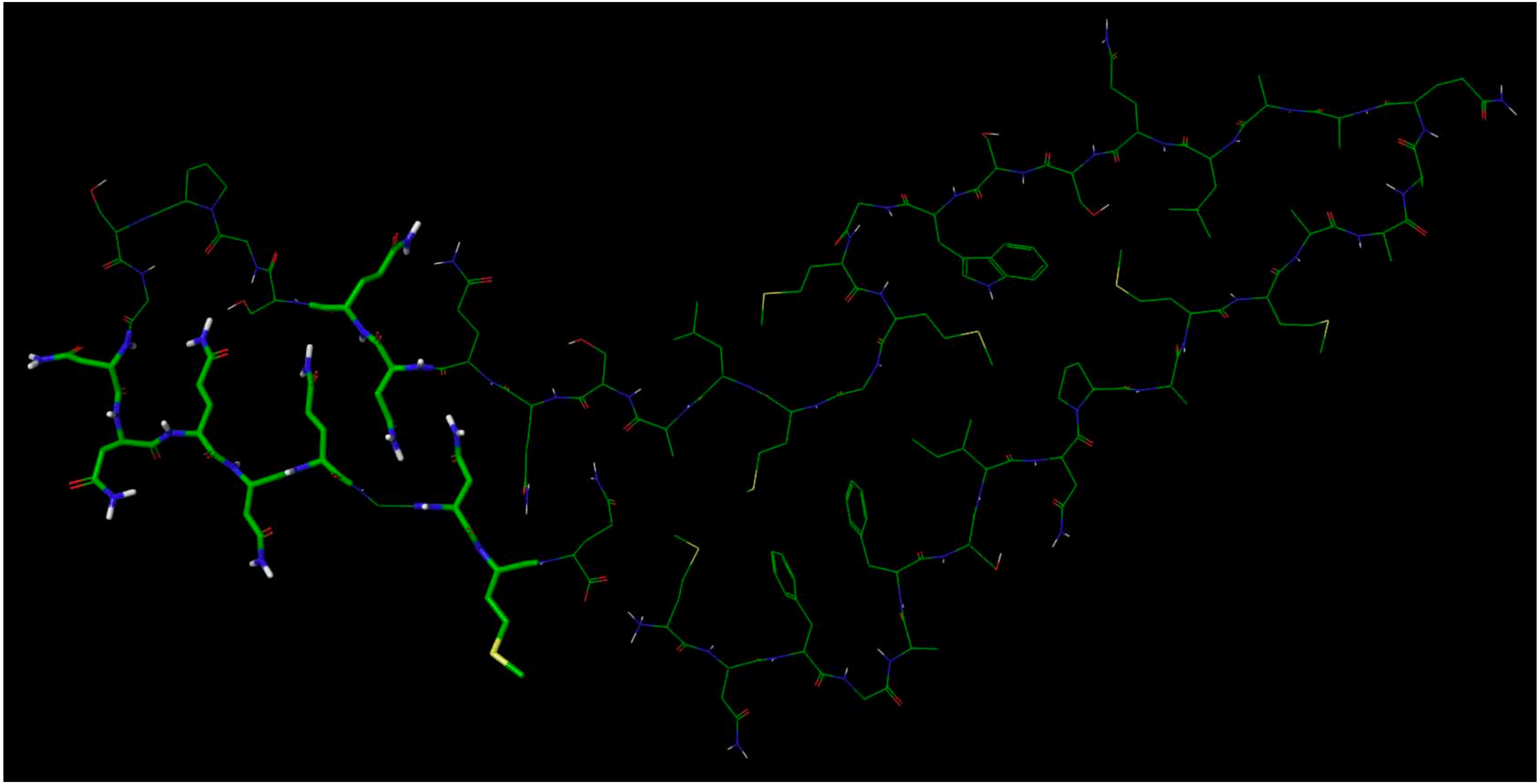

6N3A: -1778.6

MNFGAFSINPAMMAAAQAALQSSWGMMGMLASQQ**NQ**SGPSGNNQNQGNMQ

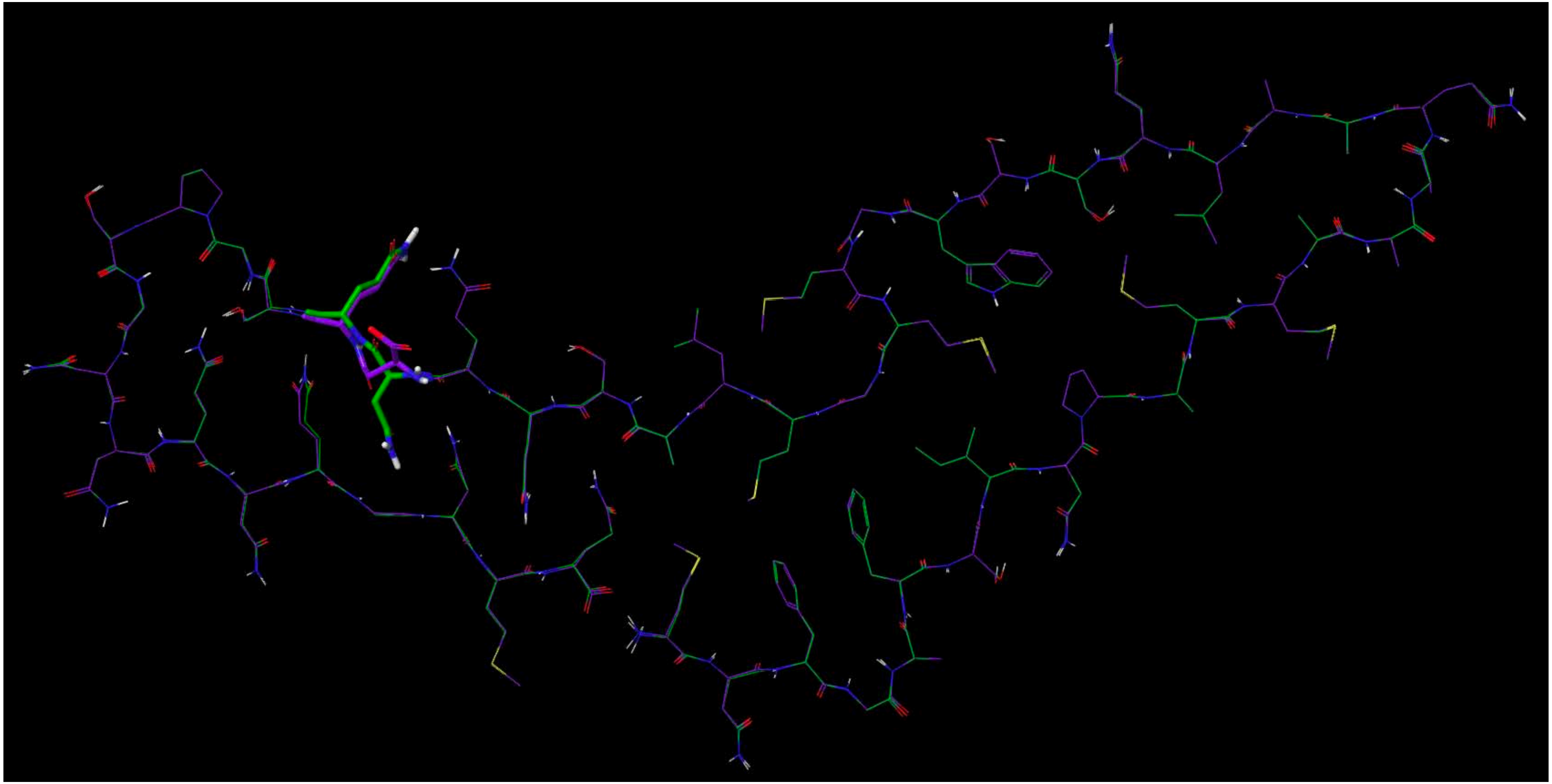

6N3A with isoaspartate 345 (N): -1710.7

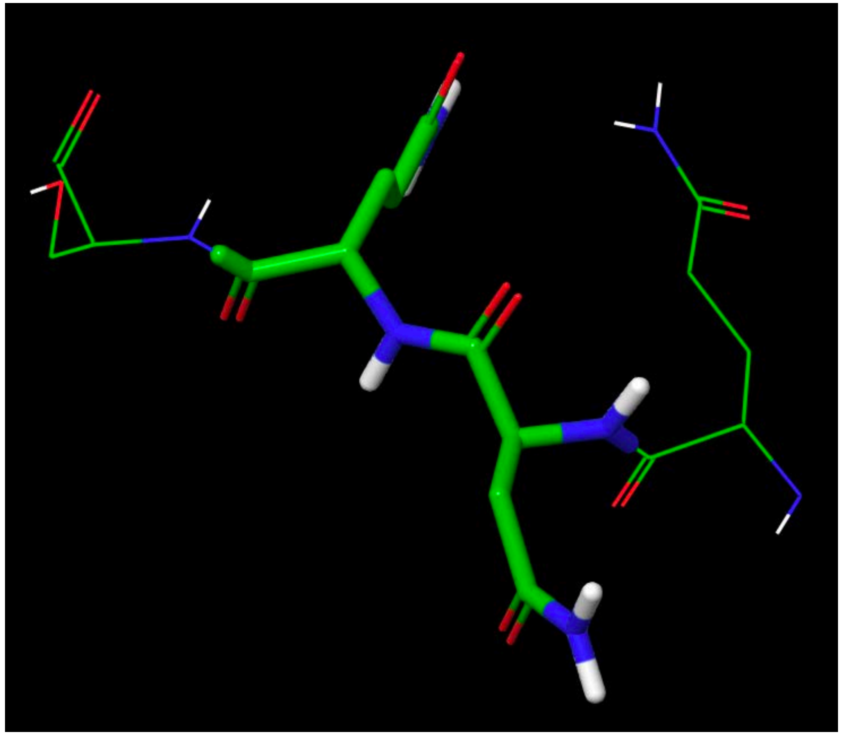

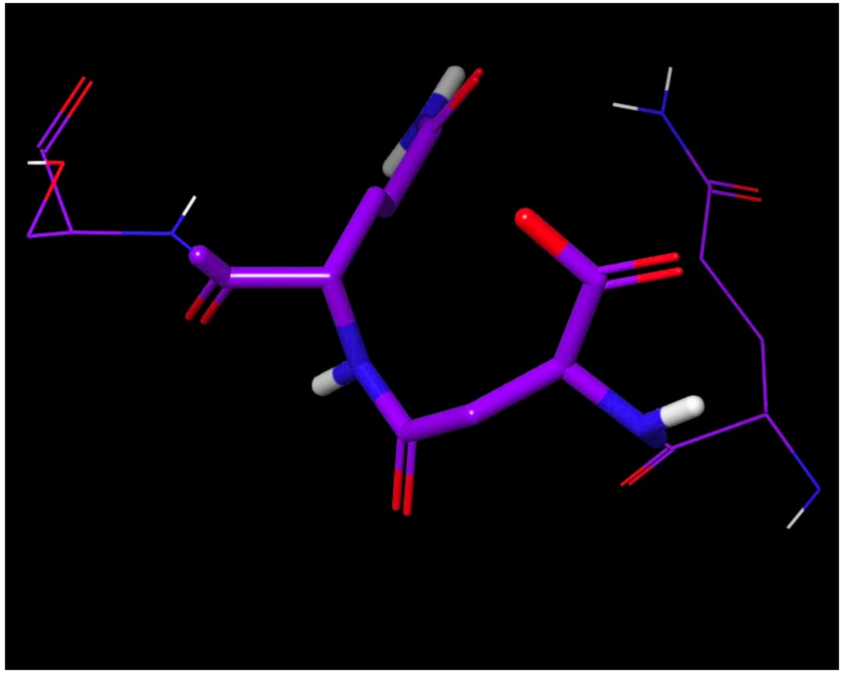

6N3A

MNFGAFSINPAMMAAAQAALQSSWGMMGMLASQQ**N****Q**SGPSGNNQNQGNMQ

6N3A: -1778.6

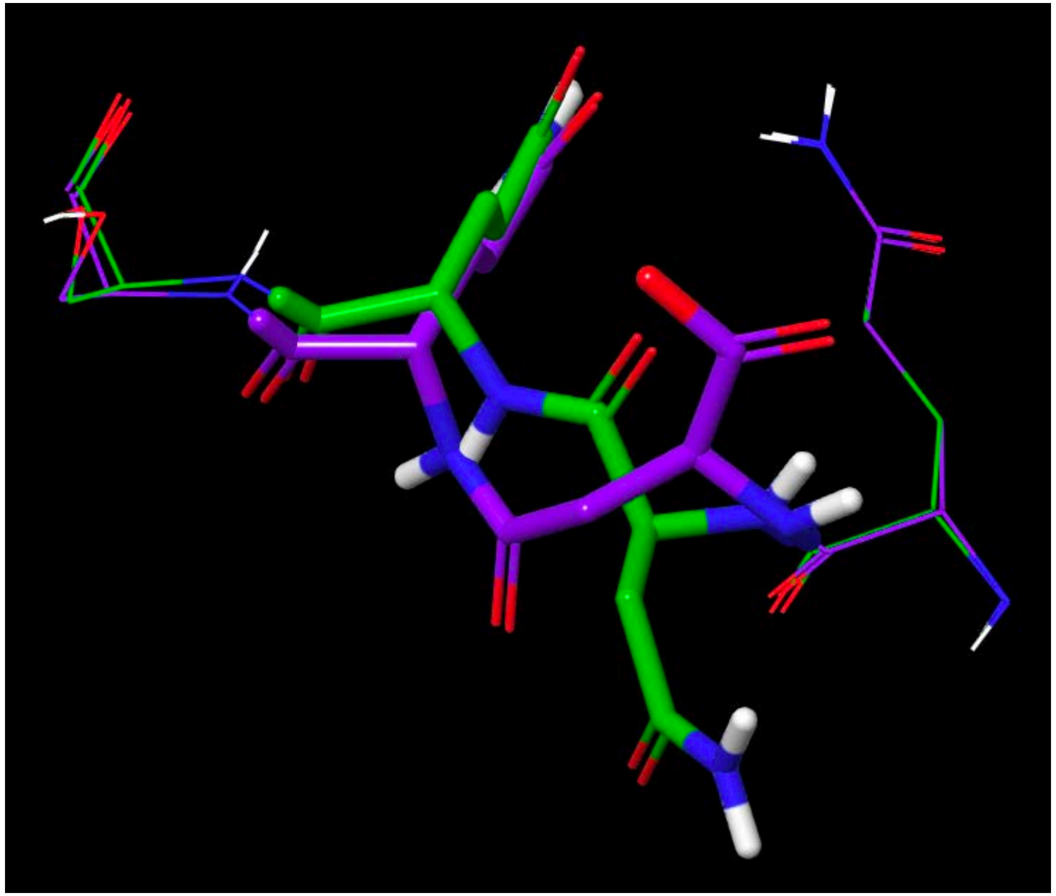

6N3A with isoaspartate 345 (N): -1710.7

6N3A: -1778.6

MNFGAFSINPAMMAAAQAALQSSWGMMGMLASQQNQSGPSG**NN**QNQGNMQ

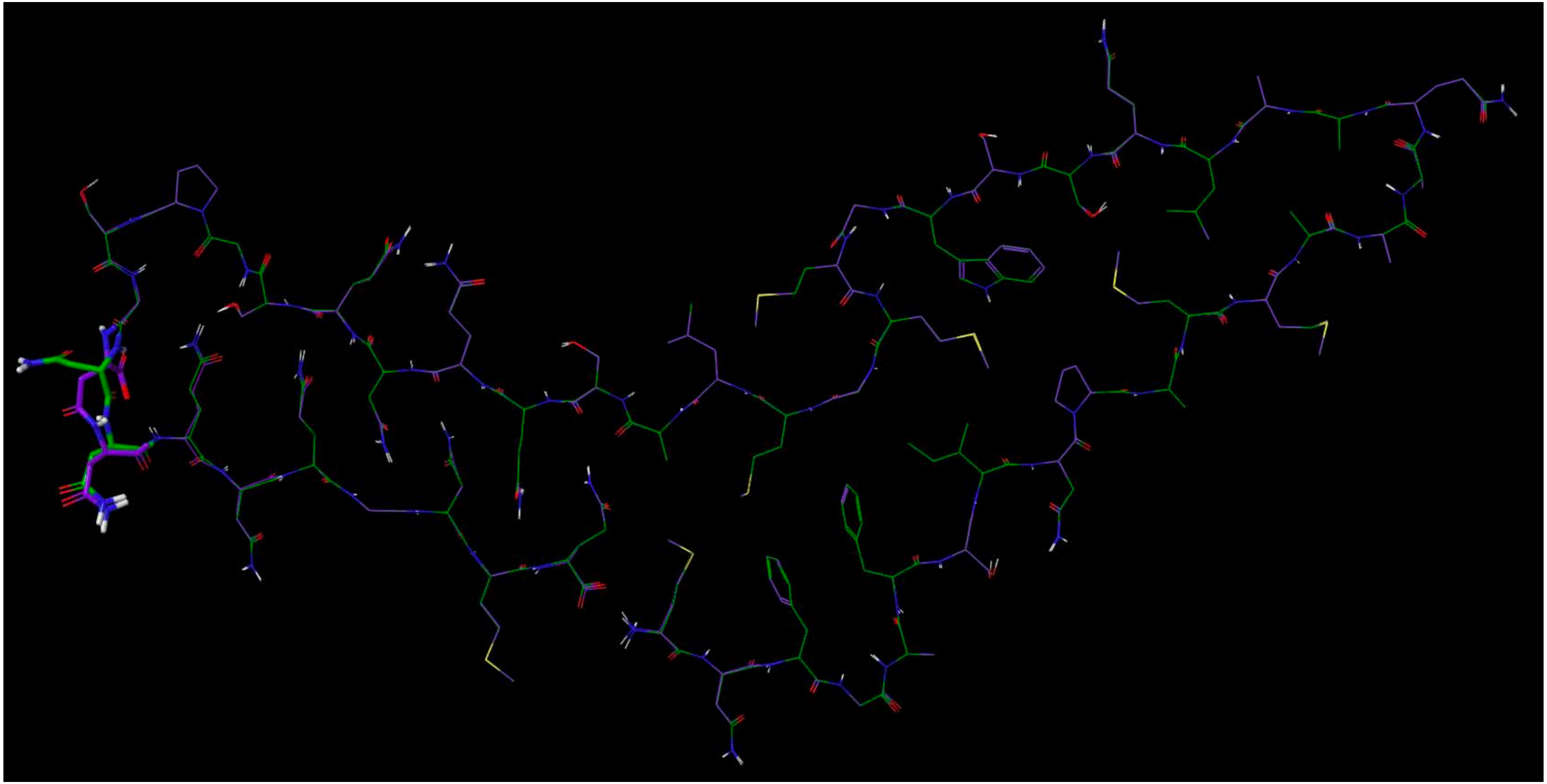

6N3A with isoaspartate 352: -1703.7

6N3A

MNFGAFSINPAMMAAAQAALQSSWGMMGMLASQQNQSGPSGNNQNQGNMQ

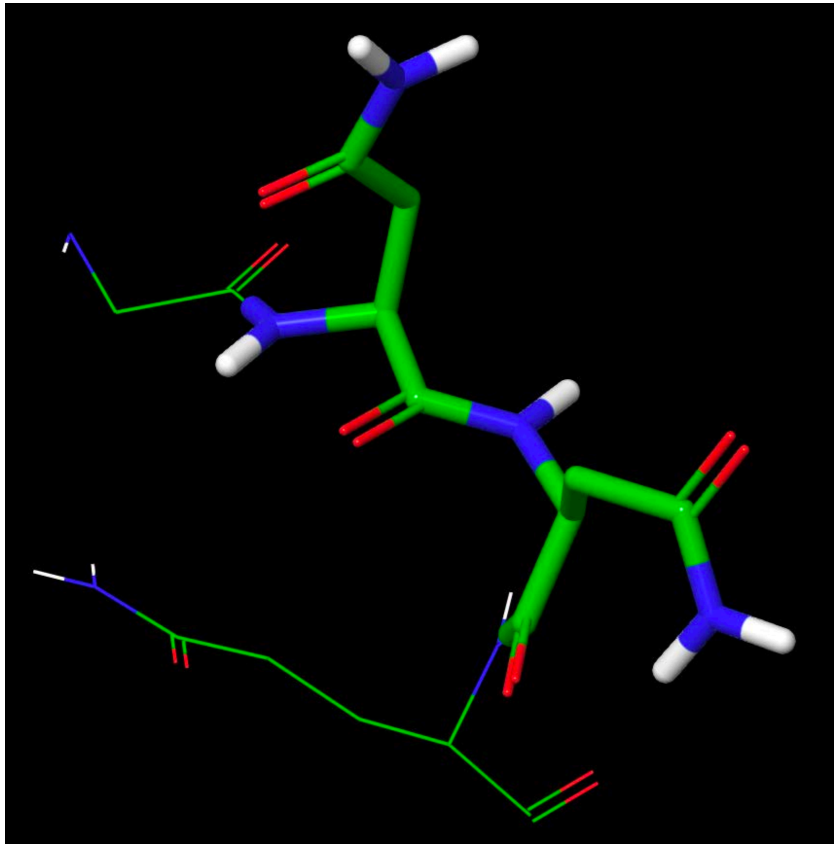

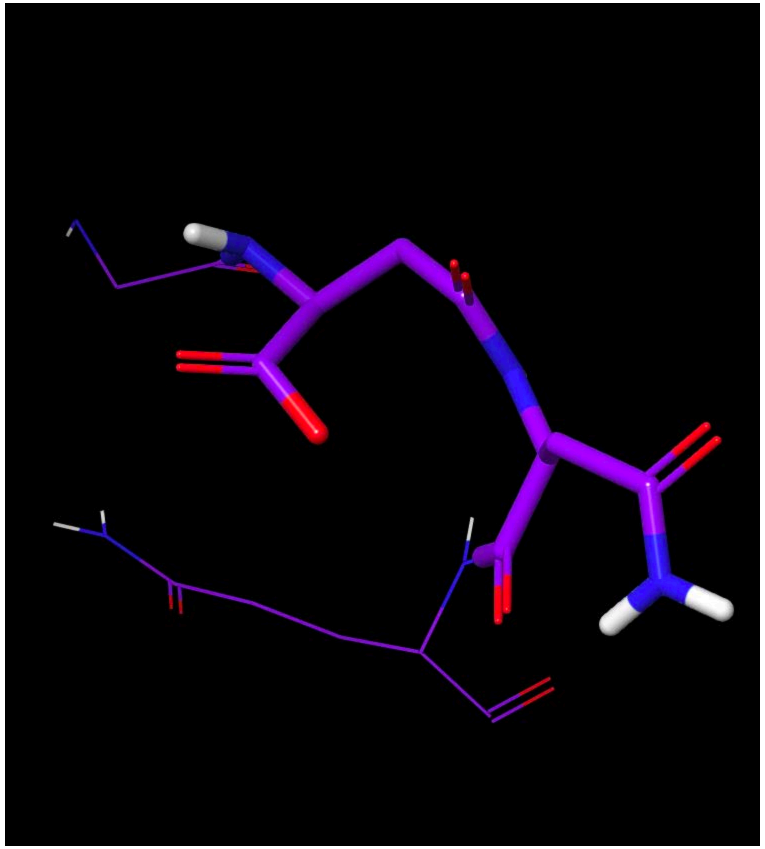

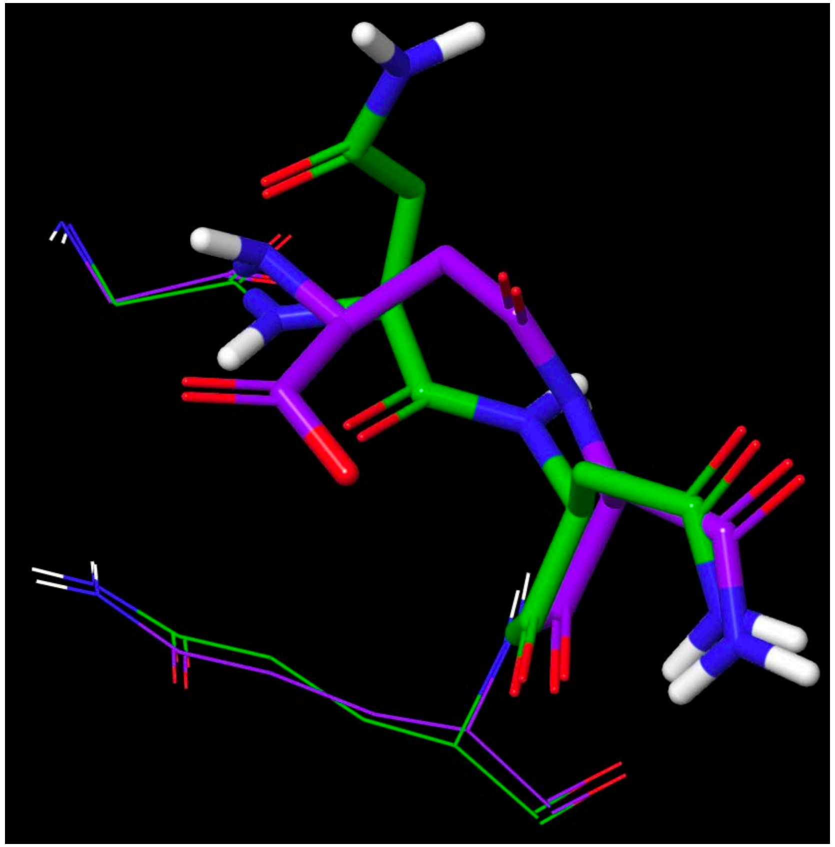

6N3A: -1778.6

6N3A with isoaspartate 352 (N): -1703.7

6N3A: -1778.6

MNFGAFSINPAMMAAAQAALQSSWGMMGMLASQQNQSGPSGNNQNQGNMQ

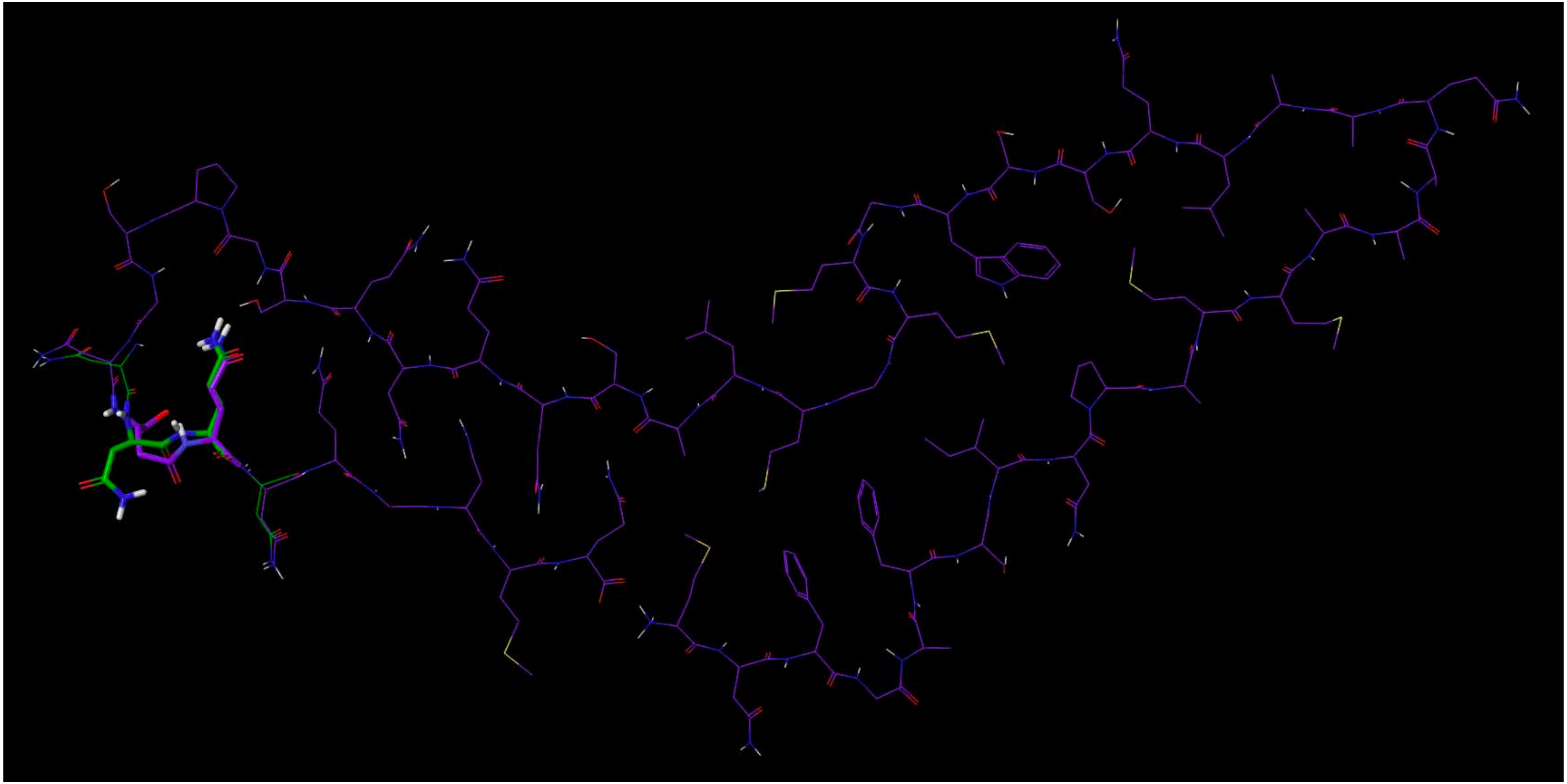

6N3A with isoaspartate 353 (N): -1697.1

MNFGAFSINPAMMAAAQAALQSSWGMMGMLASQQNQSGPSGN**NQ**NQGNMQ

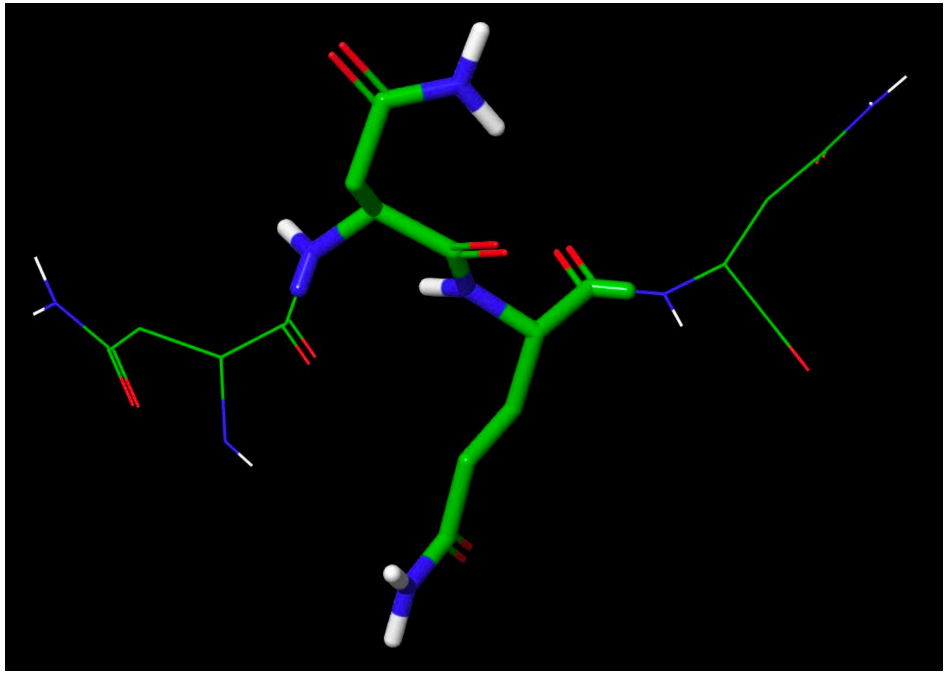

6N3A: -1778.6

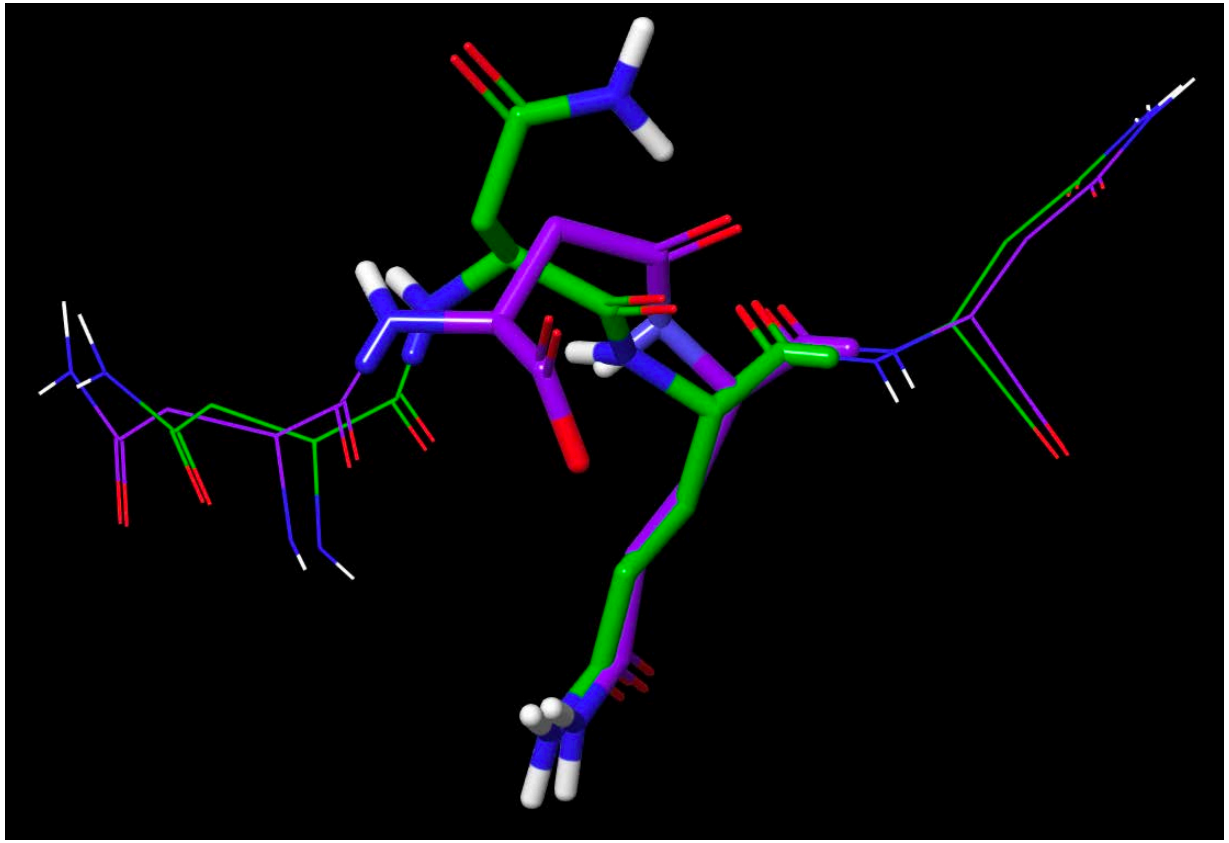

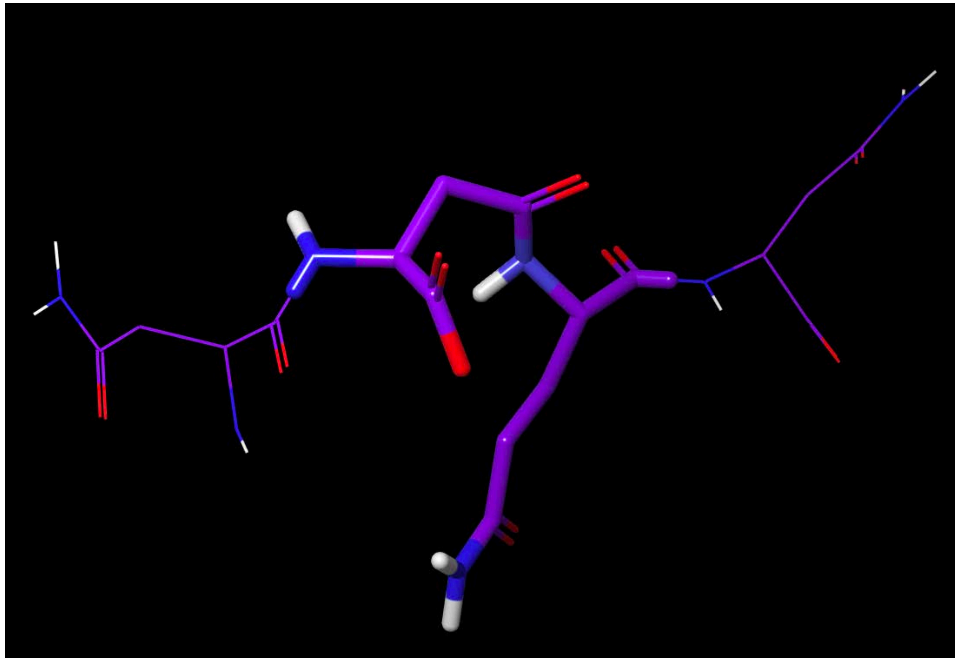

6N3A with isoaspartate 353 (N): -1697.1

6N3A: -1778.6

MNFGAFSINPAMMAAAQAALQSSWGMMGMLASQQNQSGPSGNNQ**NQ**GNMQ

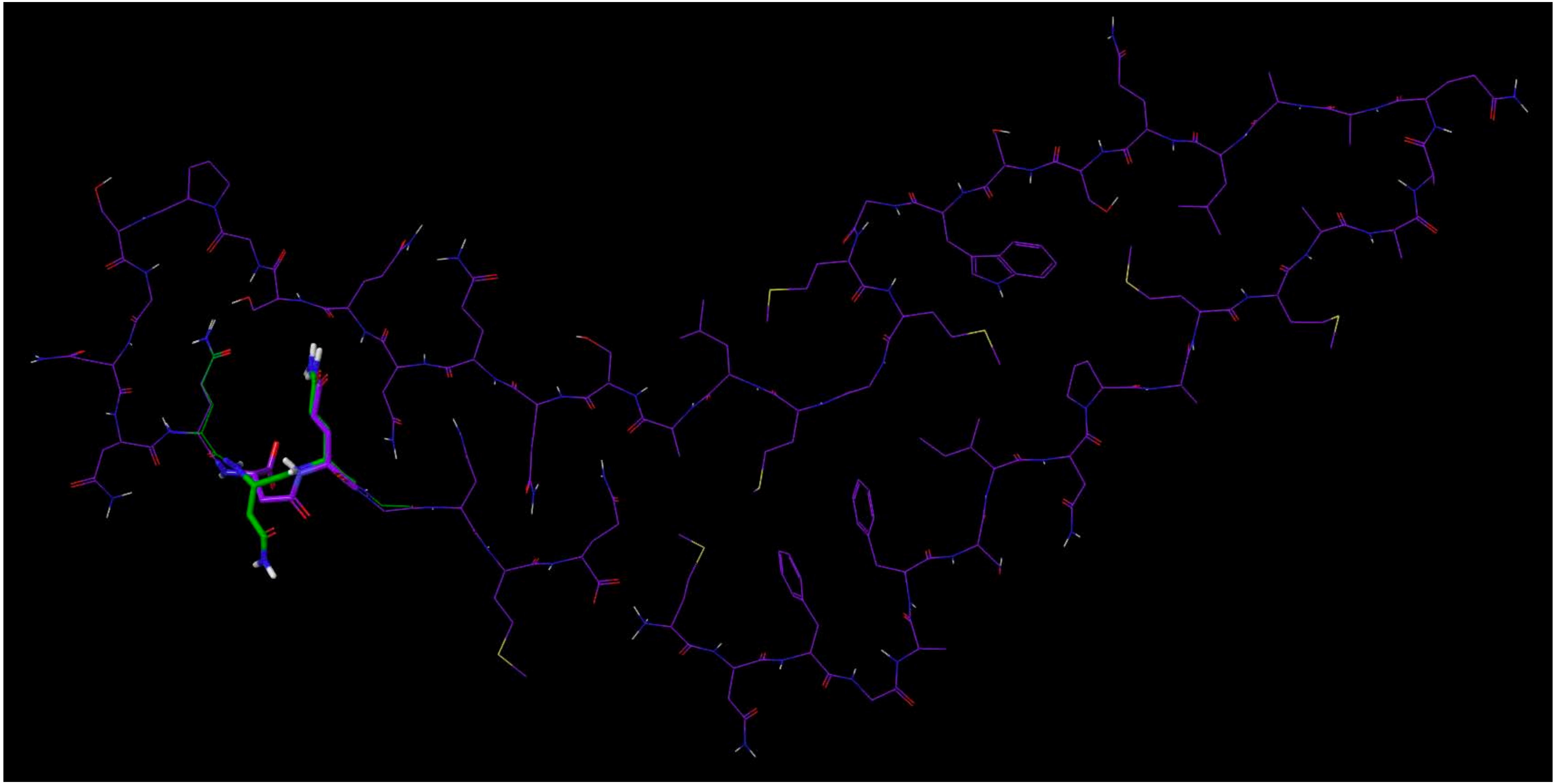

6N3A with isoaspartate 355 (N): -1723.5

6N3A

MNFGAFSINPAMMAAAQAALQSSWGMMGMLASQQNQSGPSGNNQ**NQ**GNMQ

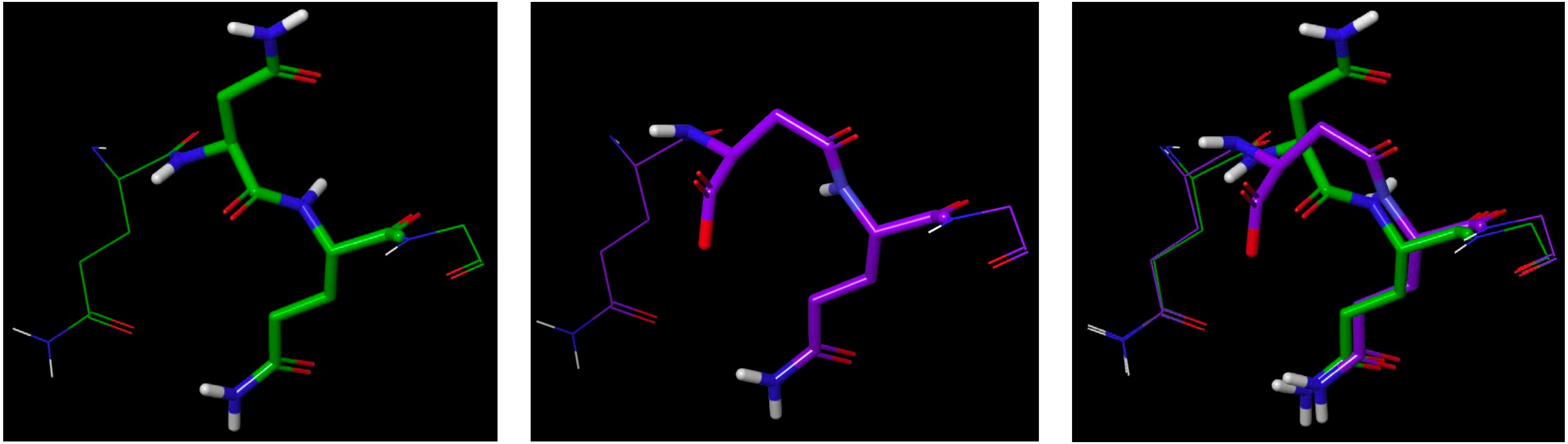

6N3A: -1778.6

6N3A with isoaspartate 355 (N): -1723.5

6N3A: -1778.6

MNFGAFSINPAMMAAAQAALQSSWGMMGMLASQQNQSGPSGNNQNQG**NM**Q

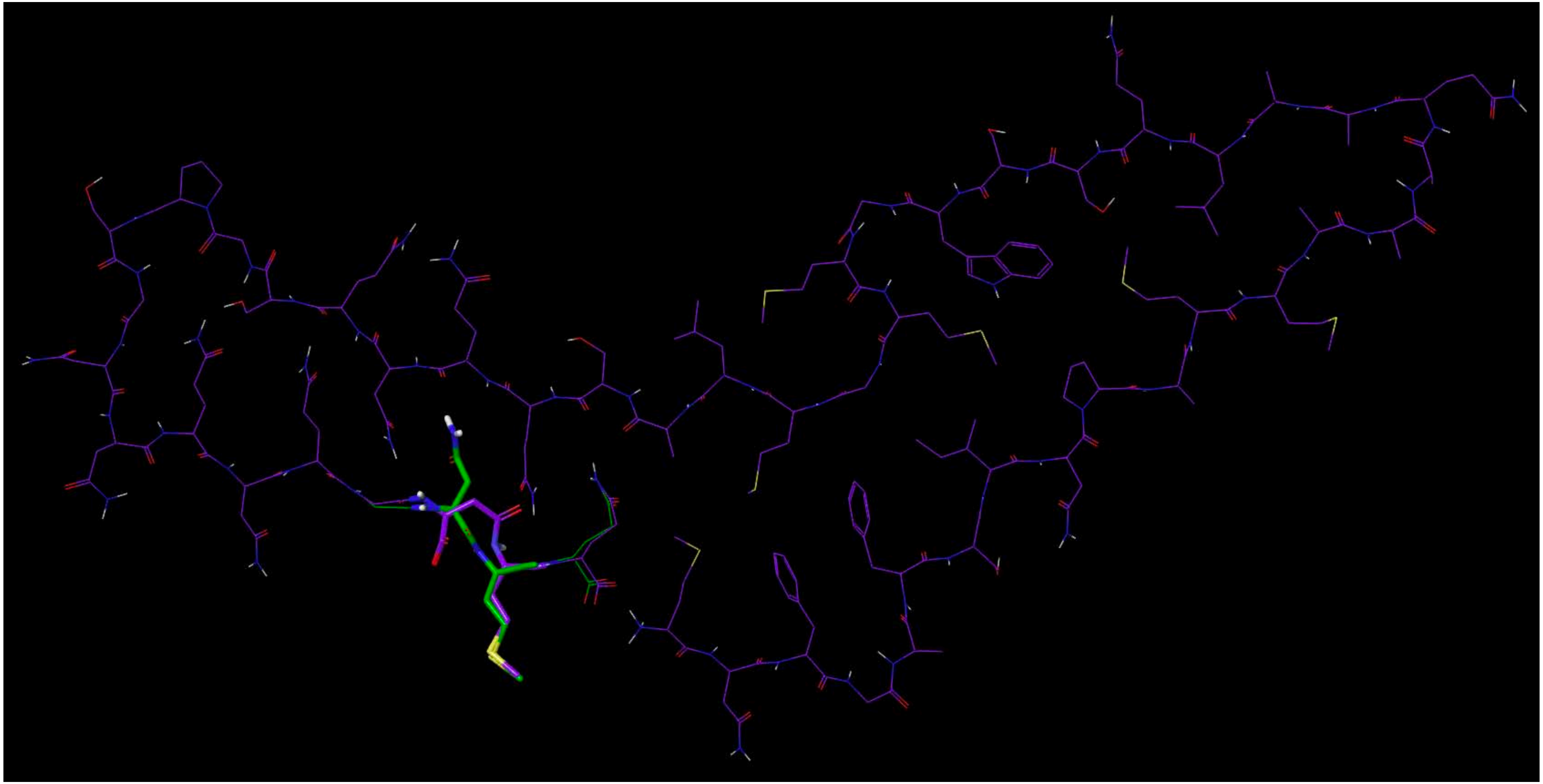

6N3A with isoaspartate 358 (N): -1693.4

MNFGAFSINPAMMAAAQAALQSSWGMMGMLASQQNQSGPSGNNQNQG**NM**Q

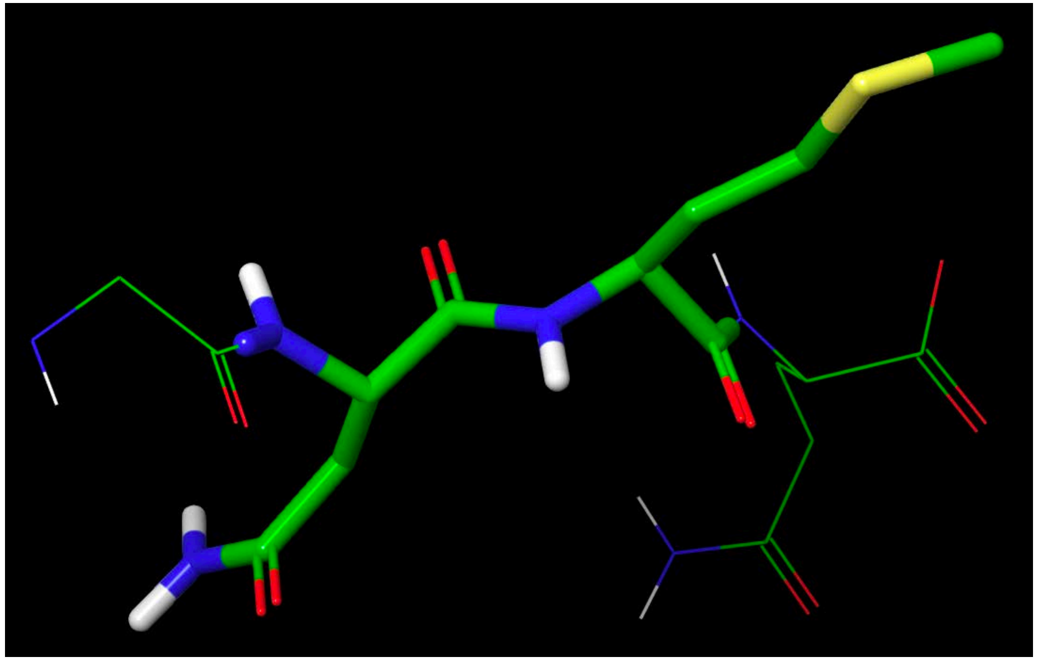

6N3A: -1778.6

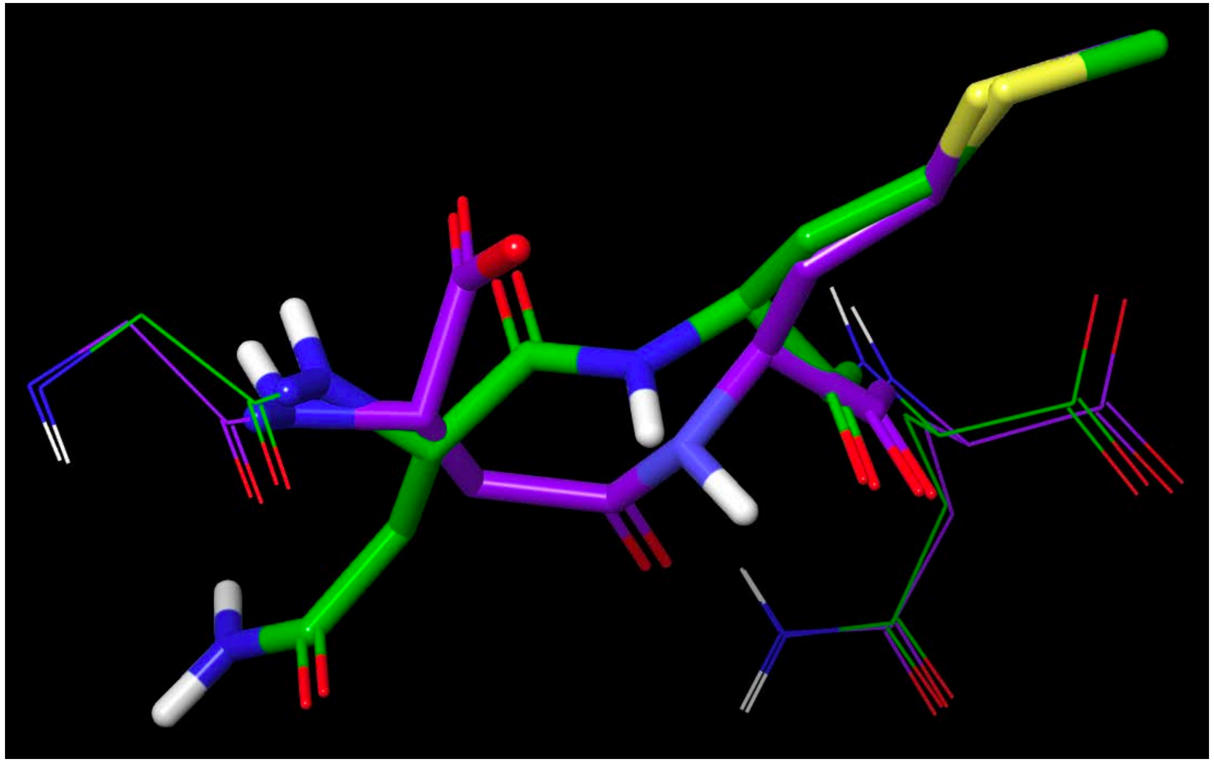

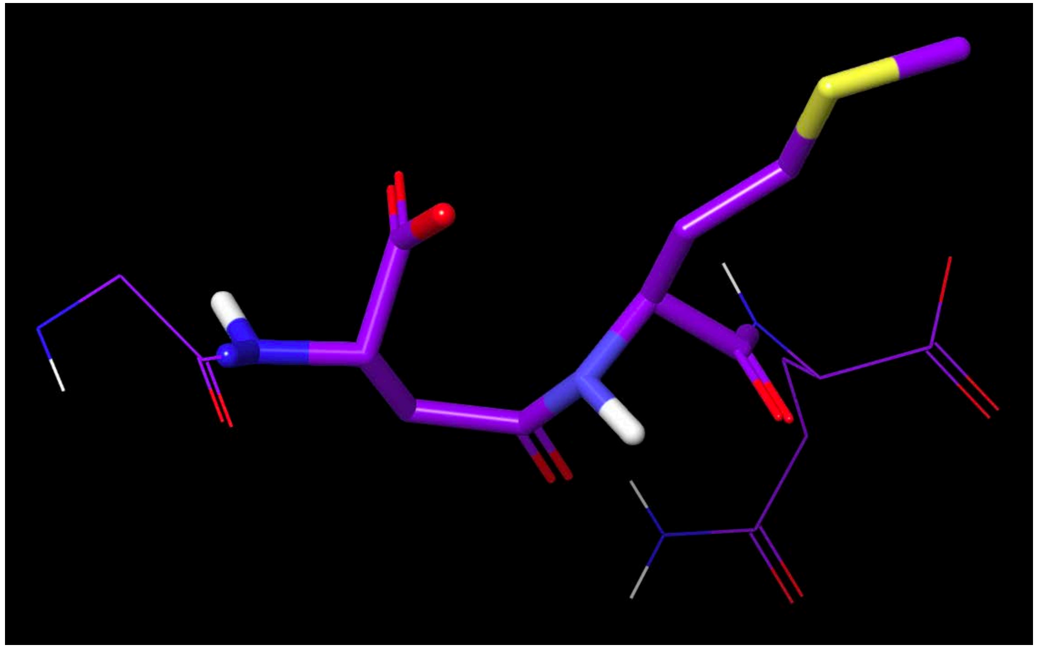

6N3A with isoaspartate 358 (N): -1693.4

6N3A: -1778.6

6N3A all sites MNFGAFSINPAMMAAAQAALQSSWGMMGMLASQQ**NQ**SGPSG**NNQNQ**G**NM**Q

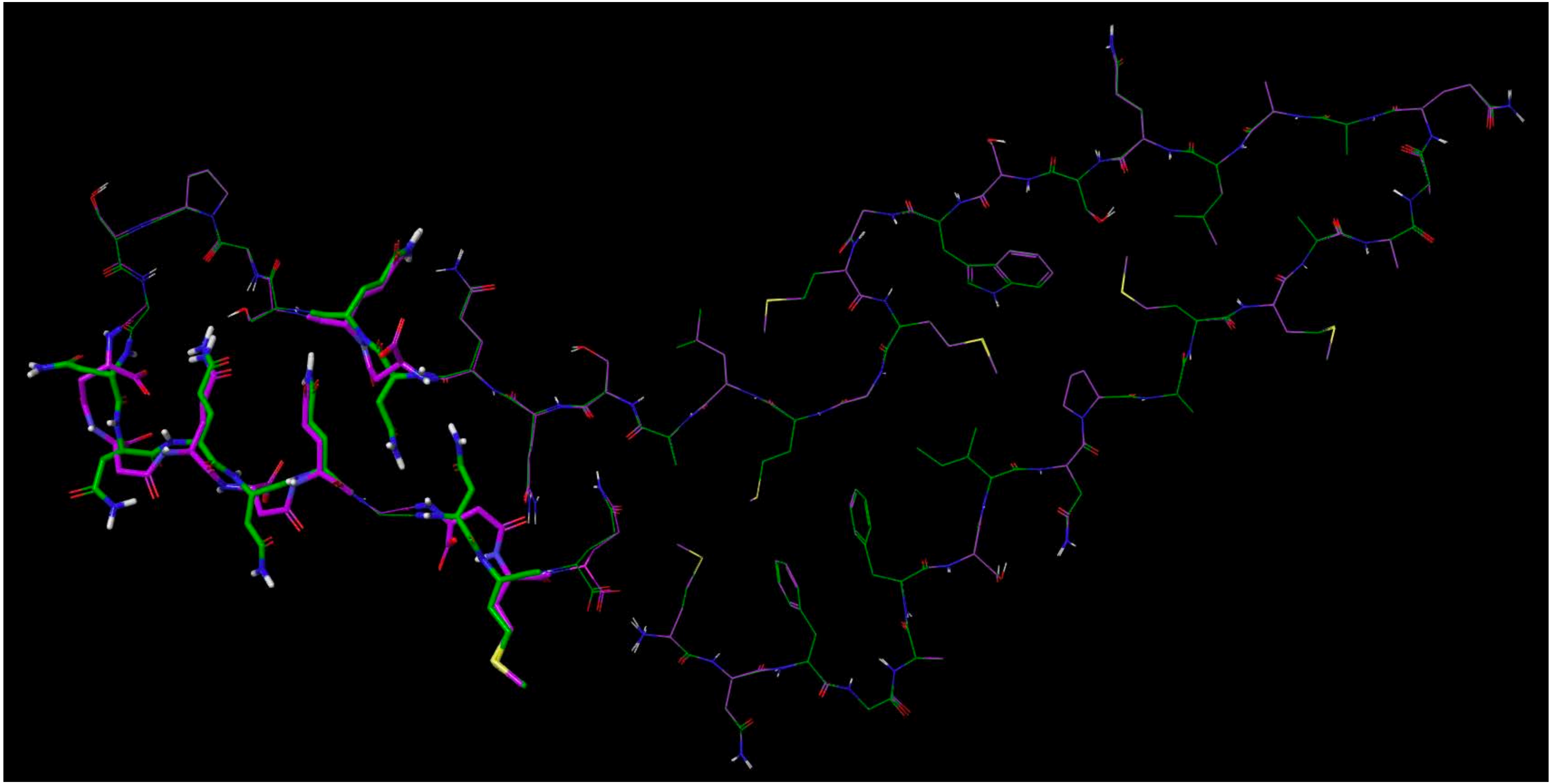

6N3A with all isoaspartate: -1360.0

6N3A all sites

MNFGAFSINPAMMAAAQAALQSSWGMMGMLASQQNQSGPSGNNQNQGNMQ

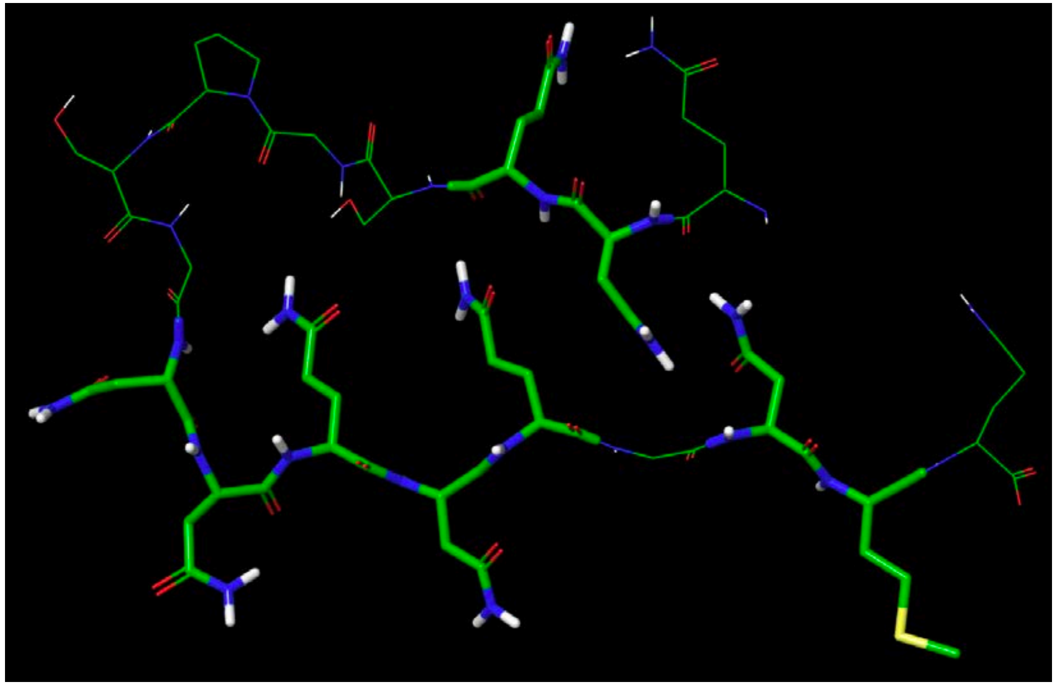

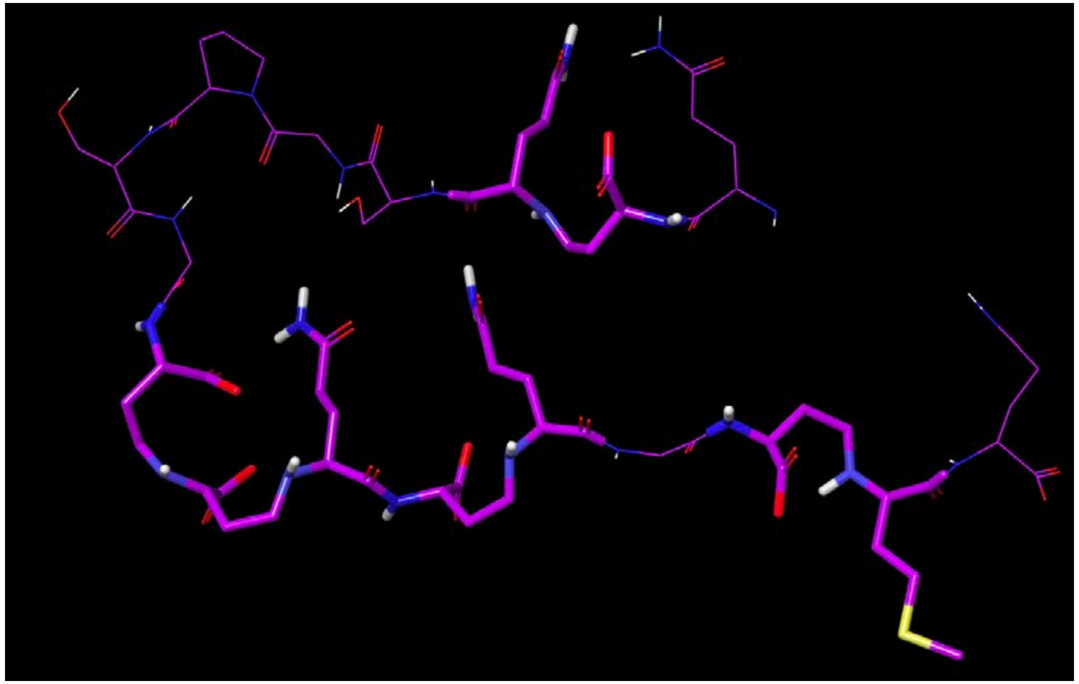

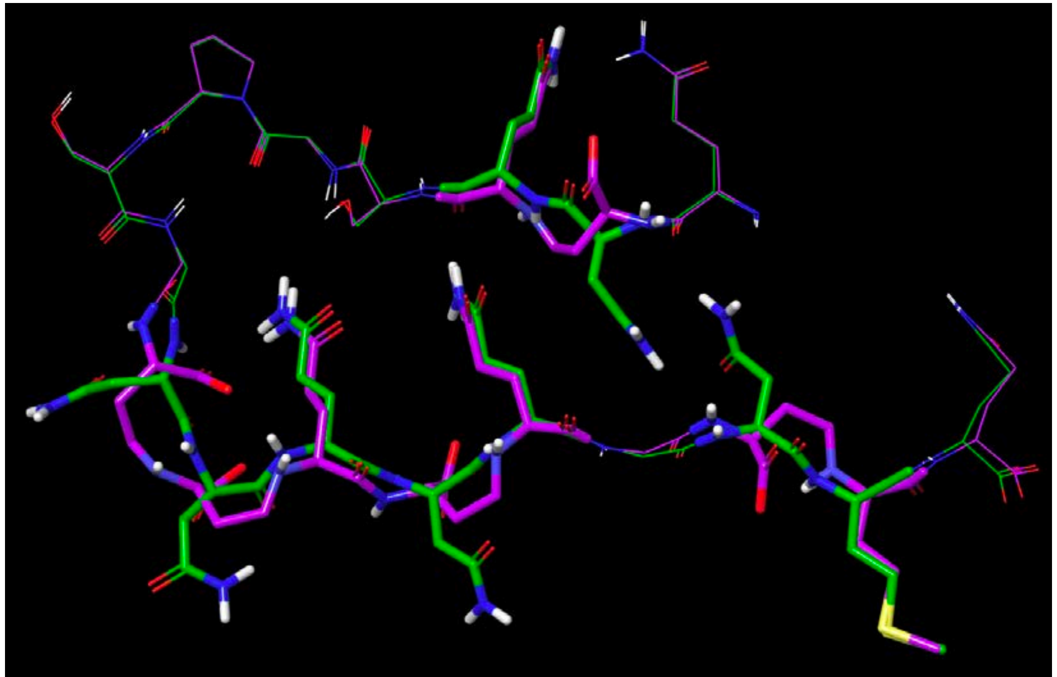

6N3A: -1778.6

6N3A with all isoaspartate: -1360.0

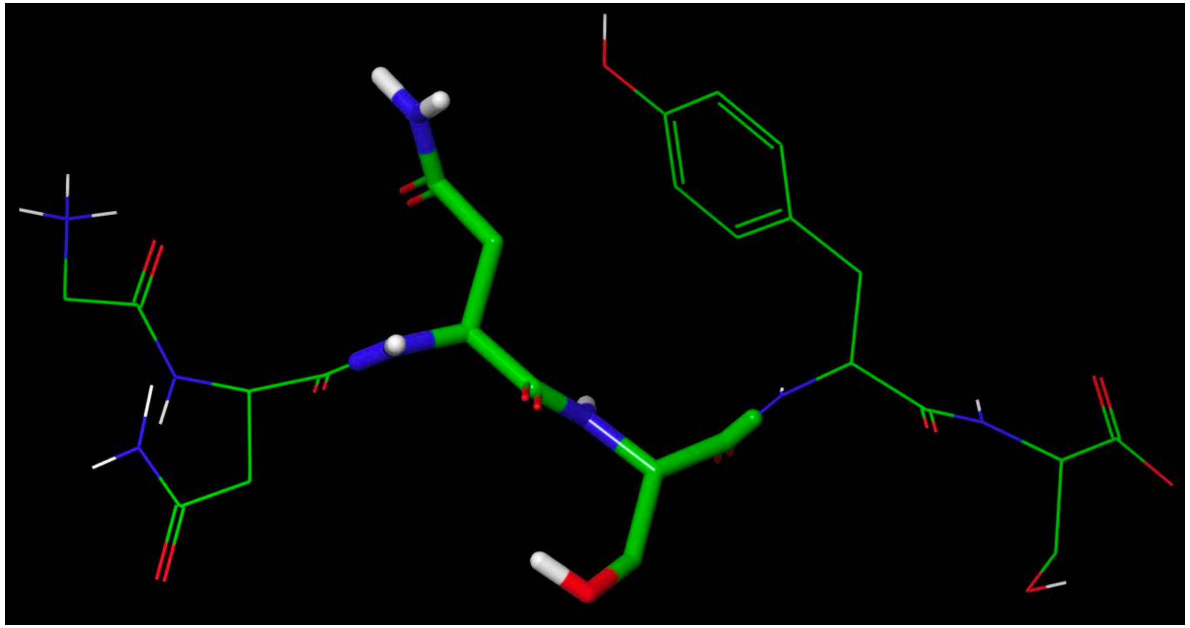

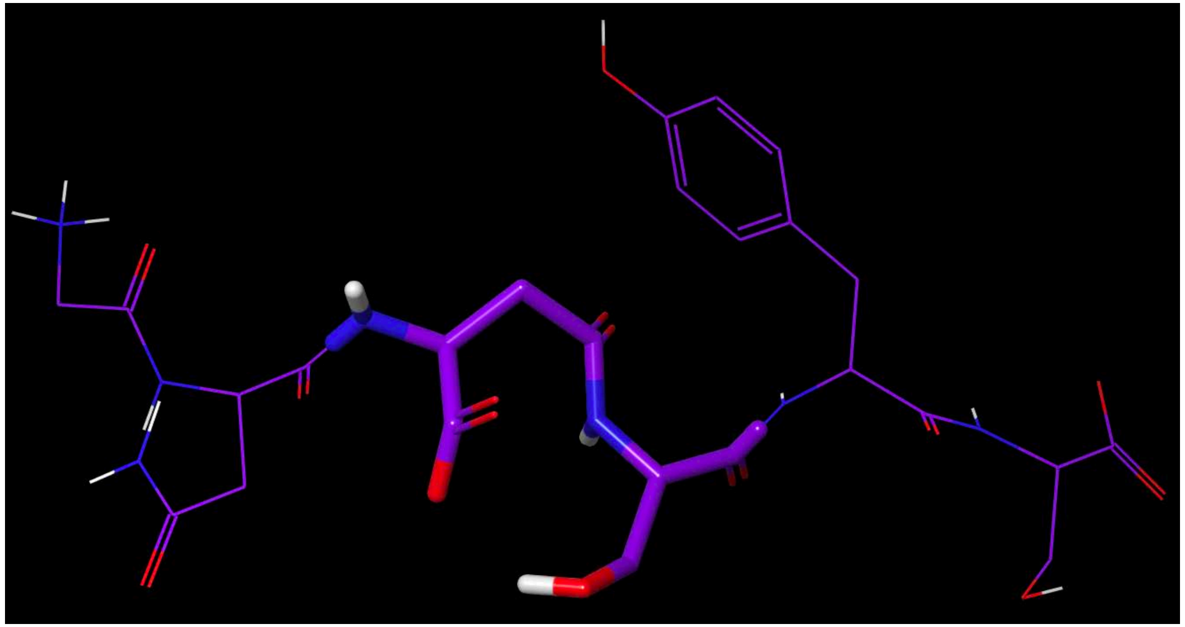

GN**NS**YS

5WIA: -251.4

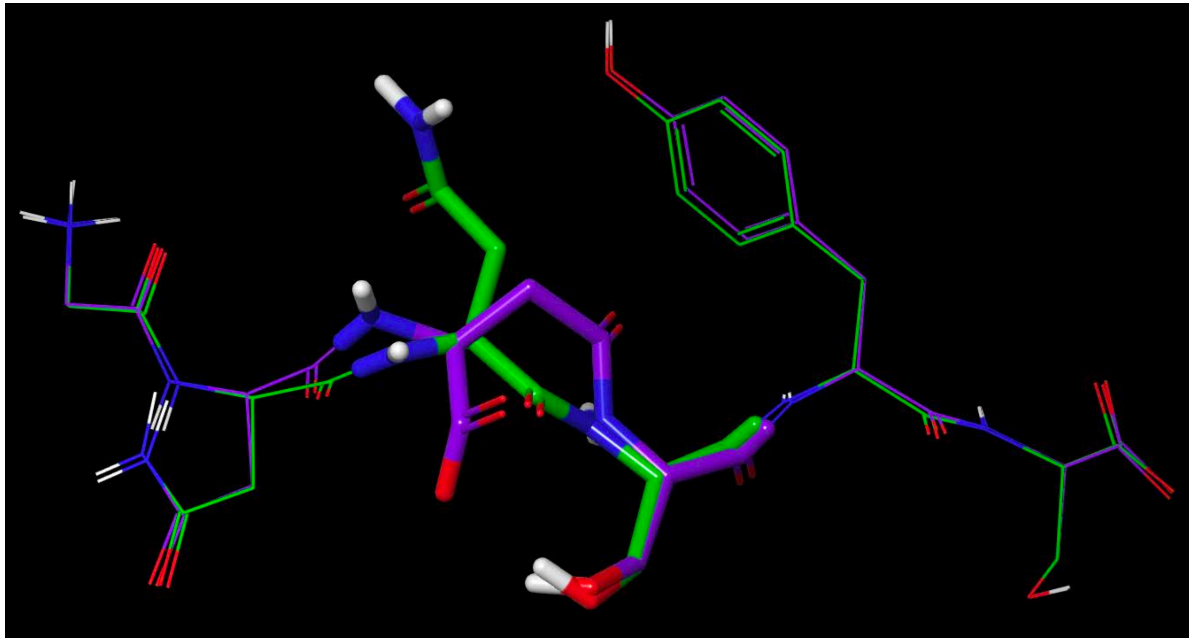

5WIA with isoaspartate: -59.5

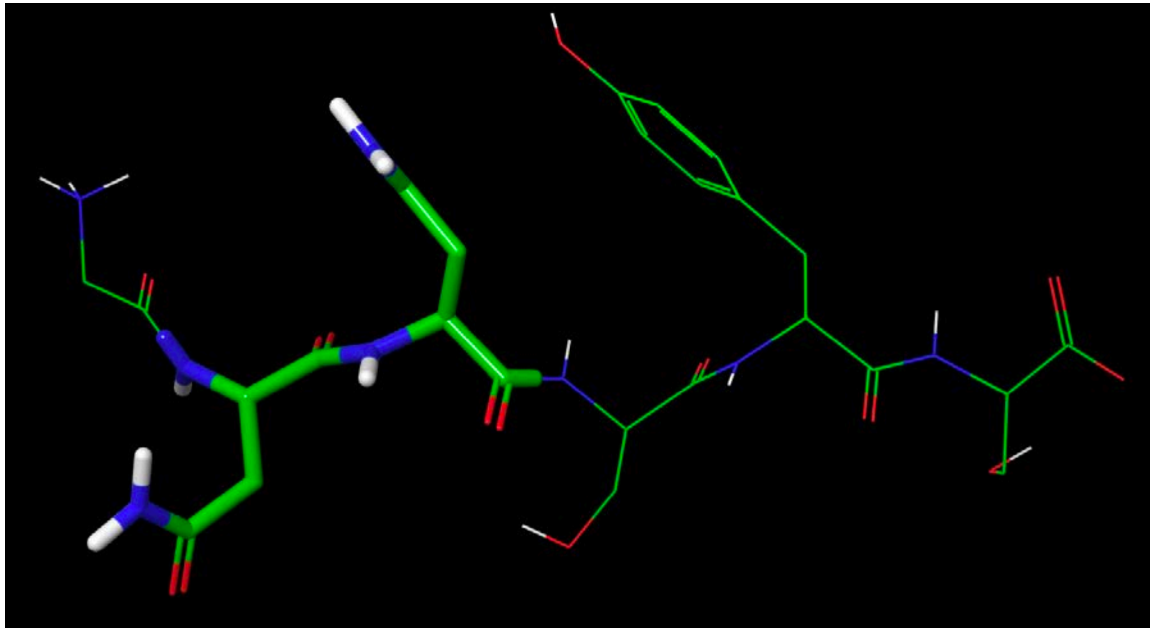

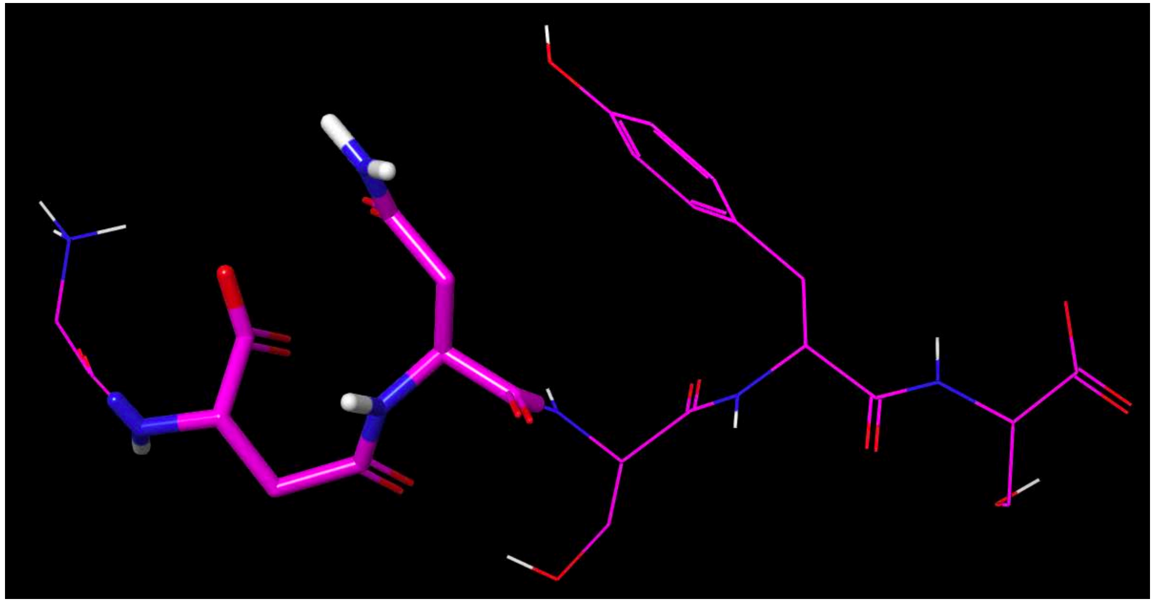

GNNSYS

5WIA: -251.4

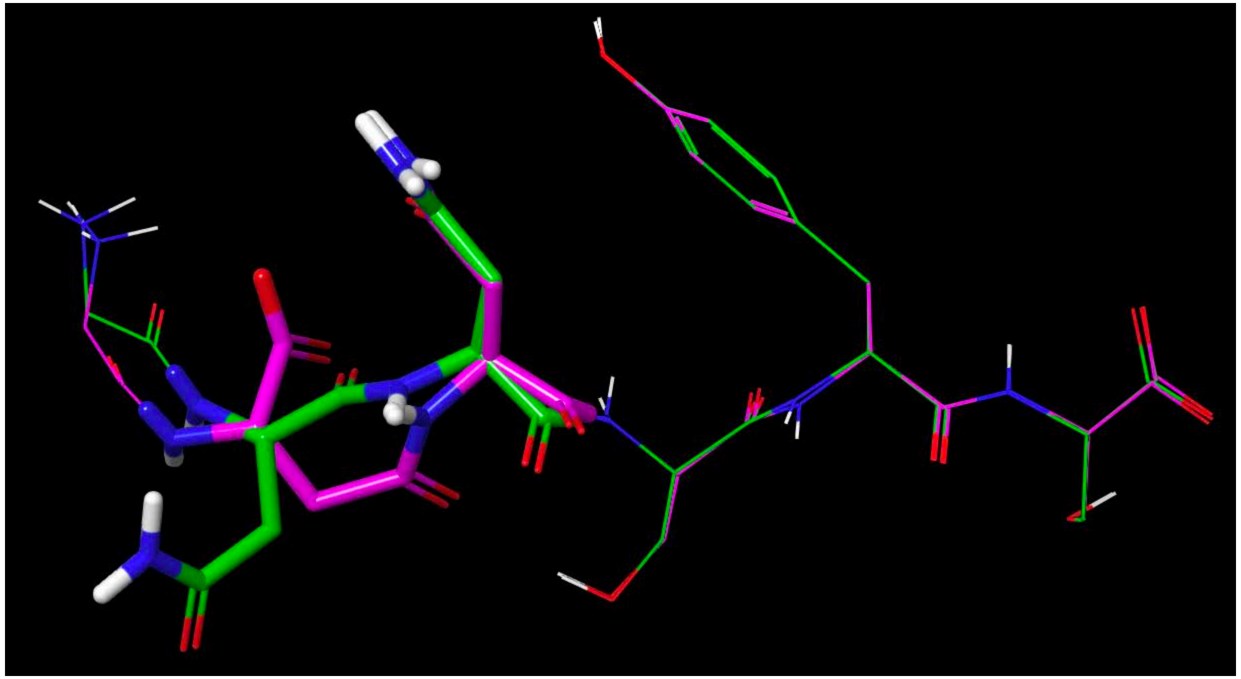

5WIA with isoaspartate 371: -130.0

GNNSYS

5WIA: -251.4

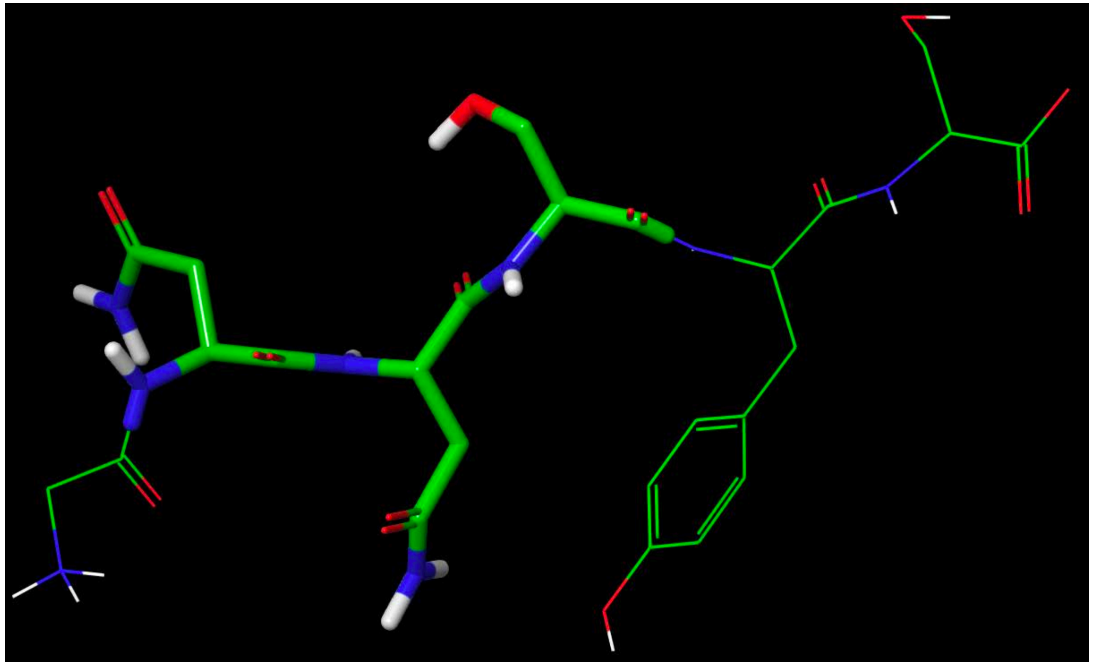

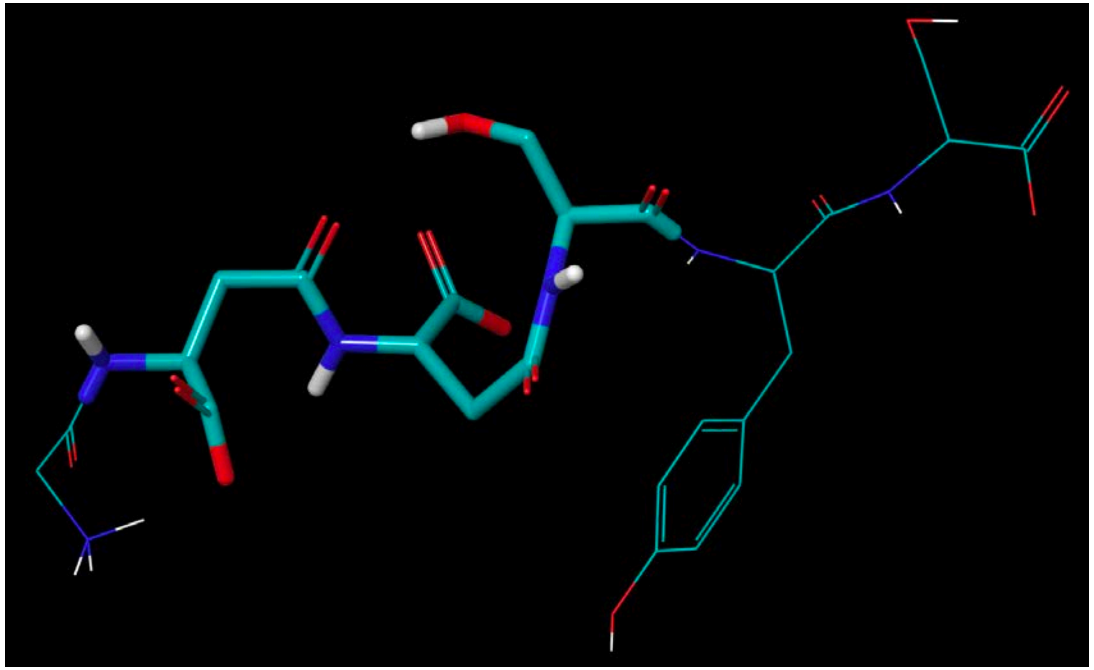

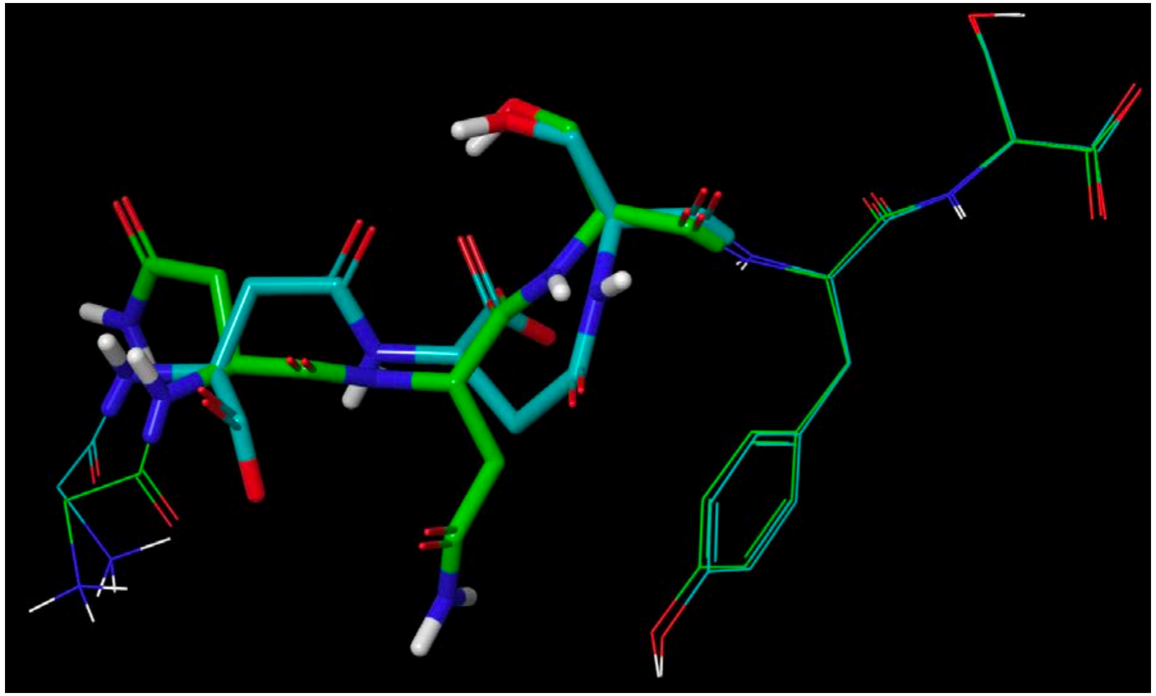

5WIA with isoaspartate 370 and 371: +141.3

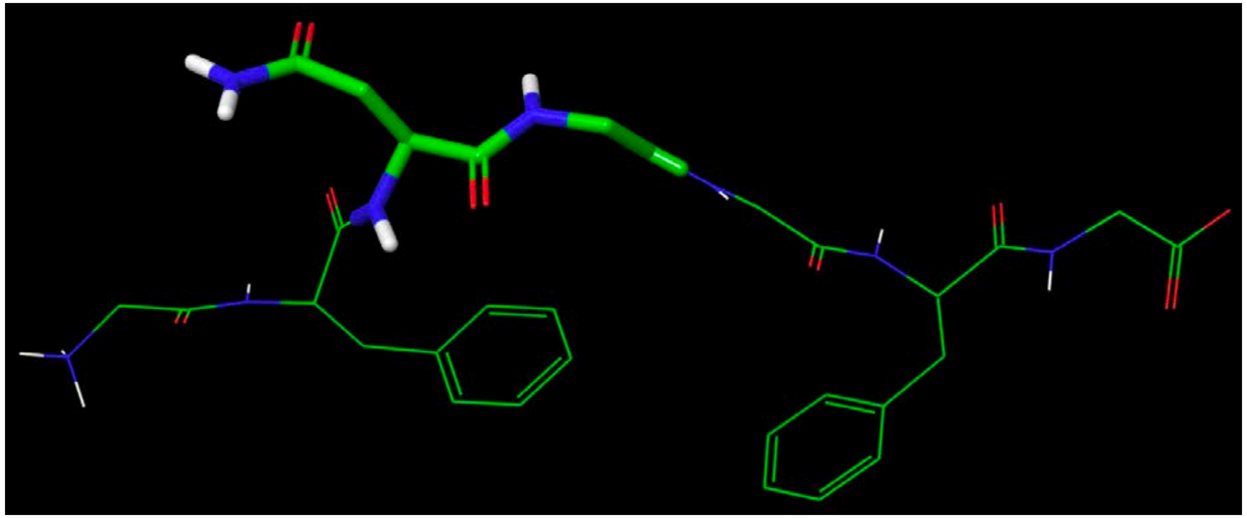

GF**N**GGFG

5WIQ: -227.3

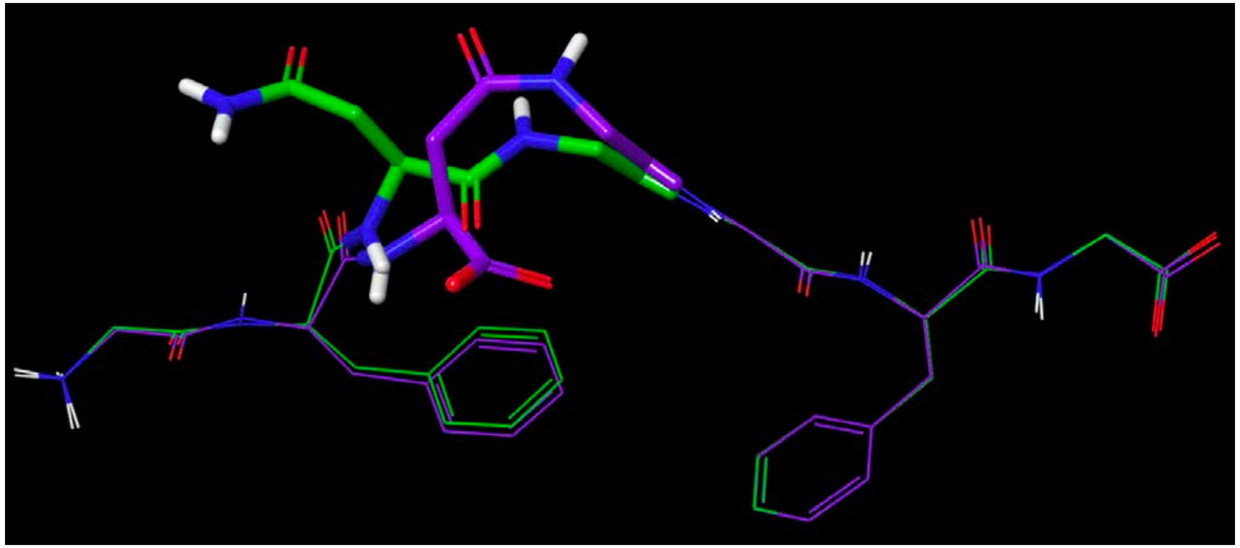

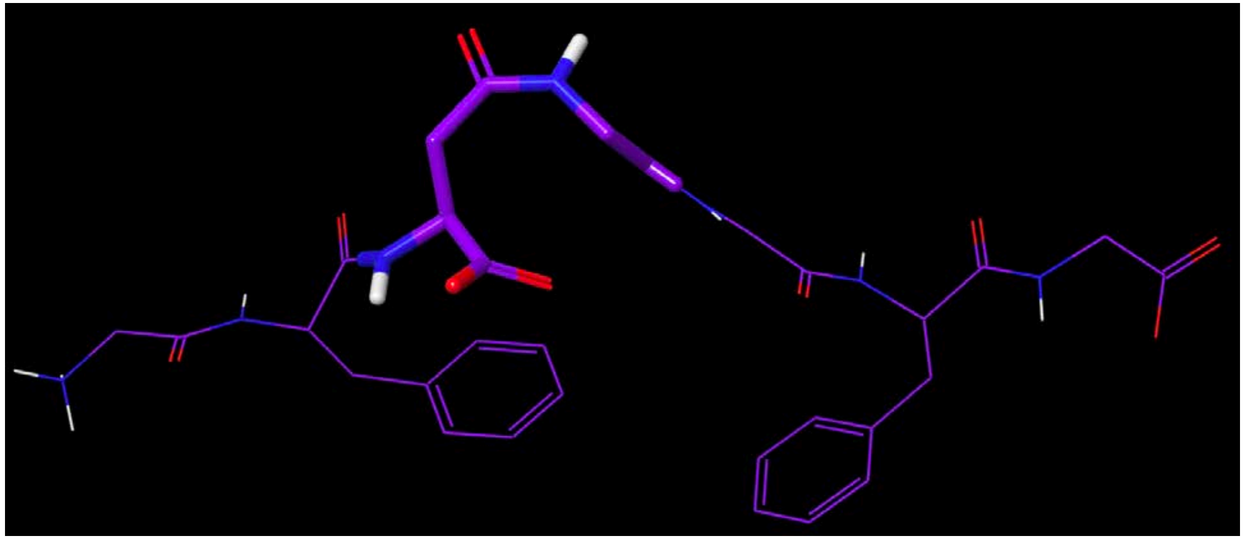

5WIQ with isoaspartate: -138.8
