# Supplementary material for: TDP-43 proteinopathy in ALS is triggered by loss of ASRGL1 and associated with HML-2 expression
Source: Nat Commun. 2024 May 16;15:4163. doi: 10.1038/s41467-024-48488-7 (PMC11099023; doi:10.1038/s41467-024-48488-7)
Supplement: Supplementary file 6 — Reporting Summary [file 41467_2024_48488_MOESM6_ESM.pdf]

nature portfolio

Corresponding author(s): Avindra Nath

Last updated by author(s): Marta Garcia Montojo

Reporting Summary

Nature Portfolio wishes to improve the reproducibility of the work that we publish. This form provides structure for consistency and transparency in reporting. For further information on Nature Portfolio policies, see our Editorial Policies and the Editorial Policy Checklist.

Please do not complete any field with "not applicable" or n/a. Refer to the help text for what text to use if an item is not relevant to your study.
For final submission: please carefully check your responses for accuracy; you will not be able to make changes later.

Statistics

For all statistical analyses, confirm that the following items are present in the figure legend, table legend, main text, or Methods section.

n/a	Confirmed	
[ ]	[X]	The exact sample size (*n*) for each experimental group/condition, given as a discrete number and unit of measurement
[ ]	[X]	A statement on whether measurements were taken from distinct samples or whether the same sample was measured repeatedly
[ ]	[X]	The statistical test(s) used AND whether they are one- or two-sided<br>*Only common tests should be described solely by name; describe more complex techniques in the Methods section.*
[ ]	[X]	A description of all covariates tested
[ ]	[X]	A description of any assumptions or corrections, such as tests of normality and adjustment for multiple comparisons
[ ]	[X]	A full description of the statistical parameters including central tendency (e.g. means) or other basic estimates (e.g. regression coefficient) AND variation (e.g. standard deviation) or associated estimates of uncertainty (e.g. confidence intervals)
[ ]	[X]	For null hypothesis testing, the test statistic (e.g. *F*, *t*, *r*) with confidence intervals, effect sizes, degrees of freedom and *P* value noted<br>*Give P values as exact values whenever suitable.*
[X]	[ ]	For Bayesian analysis, information on the choice of priors and Markov chain Monte Carlo settings
[ ]	[X]	For hierarchical and complex designs, identification of the appropriate level for tests and full reporting of outcomes
[ ]	[X]	Estimates of effect sizes (e.g. Cohen's *d*, Pearson's *r*), indicating how they were calculated

Our web collection on statistics for biologists contains articles on many of the points above.

Software and code

Policy information about availability of computer code

Data collection	Illumina HiSeq 2500, Cell Ranger v6.0, Leica Bond-MAX, Axio Imager.Z2, Thermo Scientific Orbitrap Lumos, Applied Biosystems QuantStudio 6 Flex, Cytation 5, Via7.
Data analysis	Trimmomatic, Spliced Transcripts Alignment to a Reference (STAR), Cell Ranger v6.0, ACTIONet R package, batchelor R package, limma R package, Aperio, Gen5 Image Prime, ZEN 2, NGOME-Lite, JPred, Basic Local Alignment Search Tool (BLAST), Schrodinger Suites, ClusPro 2.0, Mascot Distiller, Mascot Daemon (2.6.0), GraphPad Prism, Image J, Molecular Devices MetaXpress Neurite Outgrowth.

For manuscripts utilizing custom algorithms or software that are central to the research but not yet described in published literature, software must be made available to editors and reviewers. We strongly encourage code deposition in a community repository (e.g. GitHub). See the Nature Portfolio guidelines for submitting code & software for further information.

Data

Policy information about availability of data

All manuscripts must include a data availability statement. This statement should provide the following information, where applicable:

- Accession codes, unique identifiers, or web links for publicly available datasets
- A description of any restrictions on data availability
- For clinical datasets or third party data, please ensure that the statement adheres to our policy

The datasets generated during current study have been deposited in the Figshare database under accession “TDP-43 proteinopathy in ALS is triggered by loss of ASRGL1 and associated with HML-2 expression”. Public databases have been used for sequence alignment of proteins (PDB, Sprot Human database, Uniprot) and nucleic acids (GRCh38), protein interactions (BioGRID), and gene ontology (Gene Ontology (GO) Consortium). Source data are provided with this paper as Supplementary Data 1. RNA sequencing data was obtained from the ALS Consortium of the New York Genome Center and would available upon request: https://www.nygenome.org/contact/.

Research involving human participants, their data, or biological material

Policy information about studies with human participants or human data. See also policy information about sex, gender (identity/presentation), and sexual orientation and race, ethnicity and racism.

Reporting on sex and gender	Sex of donors of samples analyzed was always reported (sex assigned at birth). Comparison between ALS samples and controls was always sex matched. See Supplementary tables 1, 2 and 3.
Reporting on race, ethnicity, or other socially relevant groupings	Race and ethnicity have not been reported, since it was not available for most samples.
Population characteristics	Demographic characteristics of donors of samples analyzed are described in Supplementary tables 1, 2 and 3.
Recruitment	Brain samples: kindly provided by the institutions described in Supplementary Table 2 following institutions' approvals and Material Transfer Agreements. Patient characteristics and sources of the samples are described in Supplementary Table 2.<br>Induced pluripotent Stem Cells: Induced pluripotent stem cells (iPSCs) lines were generated from deidentified PBMCs from healthy human donors (Supplementary Table 3) and sporadic ALS patients using a Sendai virus method, as previously described. The lines were generated by the National Heart, Lung, and Blood Institute iPSCs core faculty at the National Institutes of Health (NIH). Samples were obtained under protocols 15-N-0125 (ALS patients) and 03-AG-0325 (Normal controls) following approval by the Institutional Review Board of NIH.<br>RNA sequencing data: RNA sequencing data was acquired from the New York Genome Center (NYGC) Consortium Database (https://www.nygenome.org/contact/) following institution's approval and Material Transfer Agreement. Demographic characteristics of the ALS patients and controls are described in Supplementary Table 1.
Ethics oversight	The lines were generated by the National Heart, Lung, and Blood Institute iPSCs core faculty at the National Institutes of Health (NIH). Samples for the generation of Induced pluripotent stem cell lines were obtained under protocols 15-N-0125 (ALS patients) and 03-AG-0325 (Normal controls) following approval by the Institutional Review Board of NIH.<br>RNA sequencing data: RNA sequencing data was acquired from the New York Genome Center (NYGC) Consortium Database (https://www.nygenome.org/contact/) following institution's approval and Material Transfer Agreement.

Note that full information on the approval of the study protocol must also be provided in the manuscript

Field-specific reporting

Please select the one below that is the best fit for your research. If you are not sure, read the appropriate sections before making your selection.

[X] Life sciences   [ ] Behavioural & social sciences   [ ] Ecological, evolutionary & environmental sciences

For a reference copy of the document with all sections, see nature.com/documents/nr-reporting-summary-flat.pdf

Life sciences study design

All studies must disclose on these points even when the disclosure is negative.

Sample size	For all in vitro experiments a minimum of 3 independent experimental replicates was performed, which is standard in the field.<br>Sample size for RNA sequencing analysis was determined by the data availability the the NY Genome Center but it meets or exceeds standards in the field (391 samples: 218 males and 173 females).<br>Sample size for immunohistochemistry (IH), western blot (WB) and PCR experiments with brain samples from ALS and controls was chosen according to or exceeding standards in the field: 16 samples for IH, 40 for WB and 39 for PCR.<br>For the experiments with motor neurons derived from induced pluripotent stem cells lines, we used 12 lines, which also meets and/or exceeds standards in the field.<br>For the in vivo experiments we used 10 animals (5 per group), which is also standard in the field. Only female mice were used due to limited sample size.
Data exclusions	For each in vitro experiment there was a setting up of the protocol. Several trials were performed during this phase whose results were not included in the analysis. Once a protocol was determined to be optimal, the experiment was replicated independently at least 3 times. No data were excluded in the analysis of those replicates.
Replication	For the in vitro experiments we always performed between 3 and 7 independent replicates. The results of all replicates were averaged for the final result.
Randomization	Randomization for in vitro experiments involving cell cultures was not needed. The same cell line was used for the different treatments that were compared in each experiment (transfection with plasmids or peptides or treatment with reagents). For analysis of clinical samples, ALS samples and controls were not randomized; they were paired by sex and age. In microscopy random fields were chosen for counting cells. For in vivo experiments, mice were randomly assigned to receive either AAV vectors encoding ASRGL1 shRNAs or scrambled shRNAS.
Blinding	Investigators were not blinded for some of the experiments: RNA seq analysis, in vitro experiments involving western blotting, since the same investigator performed each experiment from group allocation to data collection. However, measurements were always confirmed by a second investigator. For experiments involving cellular or tissue staining of human clinical samples or mice, the staining was performed by a non-blinded investigator but the analysis was performed by two independent blinded investigators.

Behavioural & social sciences study design

All studies must disclose on these points even when the disclosure is negative.

Study description	Not applicable
Research sample	Not applicable
Sampling strategy	Not applicable
Data collection	Not applicable
Timing	Not applicable
Data exclusions	Not applicable
Non-participation	Not applicable
Randomization	Not applicable

Ecological, evolutionary & environmental sciences study design

All studies must disclose on these points even when the disclosure is negative.

Study description	Not applicable
Research sample	Not applicable
Sampling strategy	Not applicable
Data collection	Not applicable
Timing and spatial scale	Not applicable
Data exclusions	Not applicable
Reproducibility	Not applicable
Randomization	Not applicable
Blinding	Not applicable

Did the study involve field work? ☐ Yes ☒ No

Field work, collection and transport

Field conditions	Not applicable
Location	Not applicable
Access & import/export	Not applicable
Disturbance	Not applicable

Reporting for specific materials, systems and methods

We require information from authors about some types of materials, experimental systems and methods used in many studies. Here, indicate whether each material, system or method listed is relevant to your study. If you are not sure if a list item applies to your research, read the appropriate section before selecting a response.

Materials & experimental systems

n/a	Involved in the study	
☐	☒	Antibodies
☐	☒	Eukaryotic cell lines
☒	☐	Palaeontology and archaeology
☐	☒	Animals and other organisms
☒	☐	Clinical data
☒	☐	Dual use research of concern
☒	☐	Plants

Methods

n/a	Involved in the study	
☒	☐	ChIP-seq
☒	☐	Flow cytometry
☒	☐	MRI-based neuroimaging

Antibodies

Antibody	Vendor Source	Product #	Host Ig class	Clone	Assay
**ASRGL1**	Atlas	HPA055572	Rabbit IgG	Polyclonal	WB/ IHC/IF/PLA
**ASRGL1**	Atlas	HPA029725	Rabbit IgG	Polyclonal	WB
**TDP-43**	Encor	MCA-3H8	Mouse IgG1	MCA-3H8	IF mice
**TDP-43 C-terminal**	Cell Signaling	3448	Rabbit IgG	Polyclonal	WB/ IP
**TDP-43**	Proteintech	66734-1-Ig	Mouse IgG1	Polyclonal	PLA
**Phospho-TDP43**	Millipore	MABN14	Rat IgG2a	1D3	WB
**Ubiquitin**	Cell signaling	3933	Rabbit IgG	Polyclonal	WB
**UNC13A**	Proteintech	55053-1-AP	Rabbit IgG	Polyclonal	WB
**XBP1**	Abcam	Ab37152	Rabbit IgG	Polyclonal	WB
**NeuN**	Millipore Sigma	MAB377	Mouse IgG	A60	IF
**CTIP2**	Abcam	ab18465	Rat IgG	25B6	IF mice
**Anti-rat-HRP**	Thermofisher	31470	Goat IgG	Polyclonal	WB
**Anti-mouse-HRP secondary**	Cell Signaling	7076	Horse IgG	Polyclonal	WB
**Anti-rabbit-HRP secondary**	Cell Signaling	7074	Goat IgG	Polyclonal	WB
**Anti-mouse Alexa-fluor-488**	Thermofisher	A28175	Goat IgG	Polyclonal	IF
**Anti-mouse Alexa-fluor-594**	Thermofisher	A-11005	Goat IgG	Polyclonal	IF
**Anti-rabbit Alexa-fluor-488**	Thermofisher	A-11008	Goat IgG	Polyclonal	IF
**Anti-rabbit Alexa-fluor-594**	Thermofisher	A-11012	Goat IgG	Polyclonal	IF
**Anti-rat Alexa-fluor-633**	Thermofisher	A-21094	Goat IgG	Polyclonal	IF

Validation Antibodies were validated by our laboratory and the manufacturing companies. . Citations of the use of the antibodies can be found in the listed websites:

ASRGL1 (Atlas, HPA055572, HPA029725): Validation in human brains and cell lines by IF, IHC (Orthogonal validation of protein expression using IHC by comparison to RNA-seq data of corresponding target in high and low expression tissues. Validation of protein expression in IHC by comparing independent antibodies targeting different epitopes of the protein. Validated against independent antibody) and WB (Genetic validation in WB by siRNA knockdown. Recombinant expression validation in WB using target protein overexpression). Confirmed homology with mouse protein. https://www.atlasantibodies.com/products/primary-antibodies/triple-a-polyclonals/anti-asrgl1-antibody-hpa029725-100ul/?language=en.
They were validated by us by shRNA silencing and protein overexpression (Supplementary figure 10).
TDP-43 (Encor MCA-3H8): Validated by IF and WB in human and mouse. https://encorbio.com/product/mca-3h8/
TDP-43 C-terminal (Cell signaling 3448): Validated by IF and WB in human: https://www.cellsignal.com/products/primary-antibodies/tdp43-g400-antibody/3448
TDP-43 Proteintech (66734-1-Ig): Validated by IF and WB in human. https://www.ptglab.com/products/TDP-43-Antibody-66734-1-Ig.htm
Phospho-TDP43 (Millipore, MABN14): Validation by WB in human.
Ubiquitin (Cell Signaling, 3933): Validation by WB in human. https://www.cellsignal.com/products/primary-antibodies/ubiquitin-antibody/3933
UNC13A (Proteintech, 55053-1-AP): Validation by WB in human.
https://www.ptglab.com/products/UNC13A-Antibody-55053-1-AP.htm
XBP1 (Abcam, Ab37152): Validation by WB in human. https://www.abcam.com/products/primary-antibodies/xbp1-antibody-ab37152.html#lb
NeuN (Millipore, MAB377). Validation by IF in human. https://www.emdmillipore.com/US/en/product/Anti-NeuN-Antibody-clone-A60,MM_NF-MAB377?ReferrerURL=https%3A%2F%2Fwww.google.com%2F
CTIP2 (Abcam, ab18465). Validation by IF in human. https://www.abcam.com/products/primary-antibodies/ctip2-antibody-25b6-ab18465.html#lb

Eukaryotic cell lines

Policy information about cell lines and Sex and Gender in Research

Cell line source(s): Cell lines used in this study were obtained from ATCC (HEK293T), As One International (mouse neural stem cells modified to express GFP (Catalogue number: MUBNF-01101), or generated in house from Induced pluripotent stem cell lines : neural stem cells (from a male healthy donor), neural stem cells modified to express tdtomato (from a male healthy donor), motor neurons derived from IPSc lines from 4 male/ 2 female ALS patients and 4 male/ 2female controls.

Authentication: In house produced cell lines were authenticated (by IF, RNAseq, PCR), not the purchased ones.

Mycoplasma contamination: All cell lines were negative for mycoplasma contamination.

Commonly misidentified lines (See ICLAC register): No commonly misidentified cell lines were used in the study

Palaeontology and Archaeology

Specimen provenance: Not applicable

Specime deposition: Not applicable

Dating methods: Not applicable

☐ Tick this box to confirm that the raw and calibrated dates are available in the paper or in Supplementary Information.

Ethics oversight:

Note that full information on the approval of the study protocol must also be provided in the manuscript.

Animals and other research organisms

Policy information about studies involving animals; ARRIVE guidelines recommended for reporting animal research, and Sex and Gender in Research

Laboratory animals: 3-month-old adult female C57BL/6 mice were purchased from Jackson Laboratory (Bar Harbor, Maine) and maintained in our breeding colony. All experiments involving mice were performed according to the recommendations in the "Guide for the Care and Use of Laboratory Animals of the NIH". Mice were housed in a pathogen-free barrier facility with a 12-hour light/12-hour dark cycle and ad libitum access to food and water.

Wild animals: No wild animals were used.

Reporting on sex: All mice were female

Field-collected samples: The study did not involved samples collected from the filed

Ethics oversight: The study was approved by the NINDS/ NICD/ NCCIH Institutional Animal Care and Use Committee (Protocol number: 1331-20).

Note that full information on the approval of the study protocol must also be provided in the manuscript.

Clinical data

Policy information about clinical studies
All manuscripts should comply with the ICMJE guidelines for publication of clinical research and a completed CONSORT checklist must be included with all submissions.

Clinical trial registration: The study did not involve a clinical trial.

Study protocol: The study did not involve a clinical trial.

Data collection: Diagnostic and demographic data of the brain samples analyzed were provided by the institutions donating the samples (Supplementary table 2).

Outcomes: The study did not involve clinical outcomes.

Dual use research of concern

Policy information about dual use research of concern Not applicable

Hazards

Could the accidental, deliberate or reckless misuse of agents or technologies generated in the work, or the application of information presented in the manuscript, pose a threat to:

No | Yes

- [x] [ ] Public health
- [x] [ ] National security
- [x] [ ] Crops and/or livestock
- [x] [ ] Ecosystems
- [x] [ ] Any other significant area

Experiments of concern

Does the work involve any of these experiments of concern:

No | Yes

- [x] [ ] Demonstrate how to render a vaccine ineffective
- [x] [ ] Confer resistance to therapeutically useful antibiotics or antiviral agents
- [x] [ ] Enhance the virulence of a pathogen or render a nonpathogen virulent
- [x] [ ] Increase transmissibility of a pathogen
- [x] [ ] Alter the host range of a pathogen
- [x] [ ] Enable evasion of diagnostic/detection modalities
- [x] [ ] Enable the weaponization of a biological agent or toxin
- [x] [ ] Any other potentially harmful combination of experiments and agents

Plants

Seed stocks	Not applicable
Novel plant genotypes	Not applicable
Authentication	Not applicable

ChIP-seq

Data deposition

- [ ] Confirm that both raw and final processed data have been deposited in a public database such as GEO.
- [ ] Confirm that you have deposited or provided access to graph files (e.g. BED files) for the called peaks.

Data access links<br>*May remain private before publication.*	Not applicable
Files in database submission	Not applicable
Genome browser session<br>(e.g. UCSC)	Not applicable

Methodology

Replicates	Not applicable
Sequencing depth	Not applicable
Antibodies	Not applicable
Peak calling parameters	Not applicable
Data quality	Not applicable

Software

Flow Cytometry

Plots

Confirm that:

- [ ] The axis labels state the marker and fluorochrome used (e.g. CD4-FITC).
- [ ] The axis scales are clearly visible. Include numbers along axes only for bottom left plot of group (a 'group' is an analysis of identical markers).
- [ ] All plots are contour plots with outliers or pseudocolor plots.
- [ ] A numerical value for number of cells or percentage (with statistics) is provided.

Methodology

Sample preparation

Instrument

Software

Cell population abundance

Gating strategy

- [ ] Tick this box to confirm that a figure exemplifying the gating strategy is provided in the Supplementary Information.

Magnetic resonance imaging

Experimental design

Design type

Design specifications

Behavioral performance measures

Imaging type(s)

Field strength

Sequence & imaging parameters

Area of acquisition

Diffusion MRI [ ] Used [ ] Not used

Preprocessing

Preprocessing software

Normalization

Normalization template

Noise and artifact removal

Volume censoring

Statistical modeling & inference

Model type and settings

Effect(s) tested

Specify type of analysis: ☐ Whole brain ☐ ROI-based ☐ Both

Statistic type for inference

(See Eklund et al. 2016)

Correction

Models & analysis

n/a	Involved in the study
☐	☐ Functional and/or effective connectivity
☐	☐ Graph analysis
☐	☐ Multivariate modeling or predictive analysis

Functional and/or effective connectivity

Graph analysis

Multivariate modeling and predictive analysis

This checklist template is licensed under a Creative Commons Attribution 4.0 International License, which permits use, sharing, adaptation, distribution and reproduction in any medium or format, as long as you give appropriate credit to the original author(s) and the source, provide a link to the Creative Commons license, and indicate if changes were made. The images or other third party material in this article are included in the article's Creative Commons license, unless indicated otherwise in a credit line to the material. If material is not included in the article's Creative Commons license and your intended use is not permitted by statutory regulation or exceeds the permitted use, you will need to obtain permission directly from the copyright holder. To view a copy of this license, visit http://creativecommons.org/licenses/by/4.0/
